# H-bond cooperativity: polarisation effects on secondary amides†

**DOI:** 10.1039/d2sc04271a

**Published:** 2022-10-03

**Authors:** Daniil O. Soloviev, Fergal E. Hanna, Maria Cristina Misuraca, Christopher A. Hunter

**Affiliations:** Yusuf Hamied Department of Chemistry, University of Cambridge Lensfield Road Cambridge CB2 1EW UK herchelsmith.orgchem@ch.cam.ac.uk

## Abstract

Formation of a H-bond with an amide carbonyl oxygen atom increases the strength of subsequent H-bonds formed by the amide NH, due to polarisation of the bond. The magnitude of this effect has been quantified by measuring association constants for the formation of 1 : 1 complexes of 2-hydroxylbenzamides with tri-*n*-butyl phosphine oxide. In 2-hydroxybenzamides, there is an intramolecular H-bond between the phenol OH group and the carbonyl oxygen atom. Comparison of the association constants measured for compounds with and without the 2-hydroxy group allows direct quantification of the effect of the intramolecular H-bond on the H-bond donor properties of the amide NH group. Substituents were used to modulate the strength of the intramolecular and intermolecular H-bonds. The presence of an intramolecular H-bond increases the strength of the intermolecular H-bond by more than one order of magnitude in *n*-octane solution. The increase in the H-bond donor parameter used to describe the amide NH group is directly proportional to the H-bond donor parameter of the phenol OH group that makes the intramolecular H-bond. These polarisation effects will lead to substantial cooperativity in complex systems that feature networks of non-covalent interactions, and the measurements described here provide a quantitative basis for understanding such phenomena.

## Abstract 2

Formation of an intramolecular phenol-amide H-bond leads to a dramatic increase in the H-bond donor strength of the amide NH group. Polarisation of the amide group is directly proportional to the polarity of the phenol H-bond donor.

## Graphical abstract

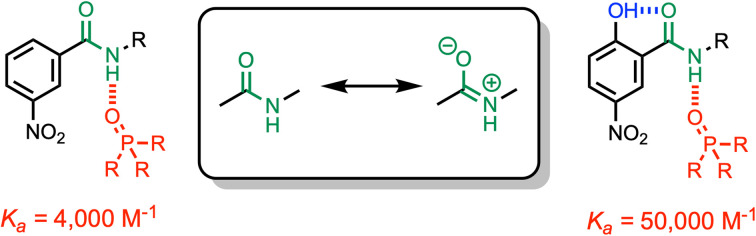

## Introduction

**Fig. 1:**
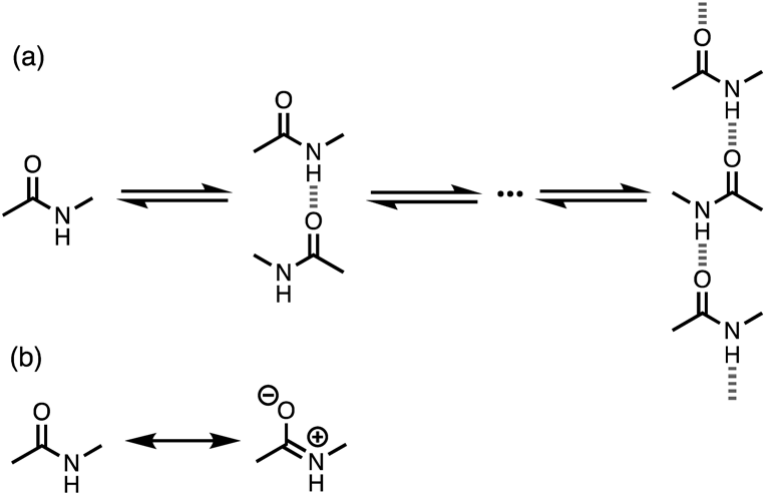
(a) Supramolecular polymerisation of secondary amides. (b) Neutral and zwitterionic amide resonance structures.

Non-covalent interactions govern a wide range of different processes in chemistry, biology and materials science. As our understanding of the fundamental nature of these interactions has developed, new supramolecular approaches have emerged to exploit these interactions in catalysis, drug design and smart materials. To a first approximation, the strength of a non-covalent interaction can be estimated based on the properties of the isolated molecules.^1^ However, for some functional groups, polar interactions like H-bonding can significantly change the molecular charge distribution, leading to cooperative effects. For example, cooperativity has been detected in the supramolecular polymerisation of secondary amides. *N*-Methyl acetamide forms chains where each molecule forms two intermolecular H-bonds, acting as both a donor through the NH group and an acceptor through the carbonyl oxygen atom (Fig. 1a).^2–4^ The enthalpy and free energy changes associated with formation of the first H-bond between two molecules of *N*-methyl acetamide are both less favourable than subsequent interactions leading to the polymer.

Computational analysis at various levels of theory suggests that the reason for this observation is that the first H-bond polarises the amide towards the more polar zwitterionic resonance form shown in Fig. 1b, so that the NH group becomes a better H-bond donor and the carbonyl oxygen atom becomes a better H-bond acceptor.^5–13^ Spectroscopic analysis of gas phase complexes confirms these predictions.^14^ These effects are likely to have important implications for understanding the properties of a wide range of different systems, because chains of H-bonded amides are a common motif in protein structure and polyamide materials.

**Fig. 2:**
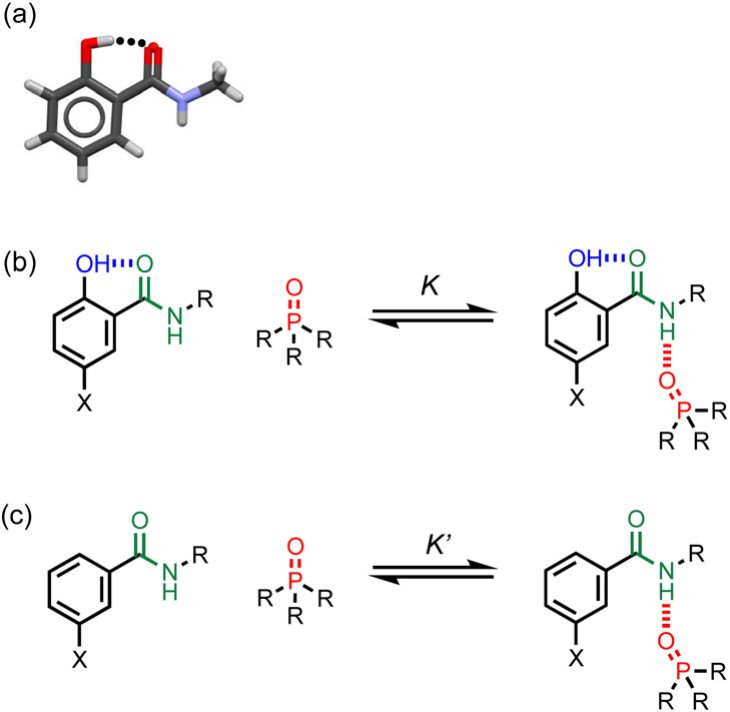
(a) X-ray crystal structure showing the intramolecular phenol-amide H-bond when X = H and R = Me (CSD ref code KUSVUP). (b) Interaction of a H-bonded amide with a phosphine oxide. (c) Reference interaction of a non-H-bonded amide with a phosphine oxide. X is a substituent that modulates the H-bond donor properties of the phenol, and R is a solubilising group.

Although the theoretical basis for H-bond cooperativity due to polarisation is well-established, experimental studies of the magnitude of the effects on non-covalent interactions in solution are limited. Spectroscopic and crystallographic studies show that formation of a H-bond to an amide group increases the length of the C–O bond and decreases the length of the C–N bond consistent with an increase in the population of the more polar structure shown in Fig. 2b.^15,16^ Measurements of intermolecular association constants indicate that this polarisation can lead to an increase in stability of an order of magnitude.^2–4,15–17^ However, a systematic investigation of the relationship between the properties of the polarising interaction and the change in H-bond properties of secondary amides has not been carried out. Here we describe an experiment to quantify these effects. We show that a H-bond formed with the carbonyl oxygen of an amide group significantly increases the stability of a H-bond formed with the amide NH. The magnitude of this polarisation effect is directly proportional to the polarity of the group that makes the interaction with the carbonyl oxygen. The experiments provide quantitative data that can be used to benchmark the development of computational treatments of non-covalent interactions in complex systems, where cooperative effects may play a significant role.

## Approach

The approach is illustrated in Fig. 2. A search of the Cambridge Structural Database (CSD) showed that 2-hydroxybenzamides have an intramolecular H-bond between the hydroxyl group and the carbonyl oxygen of the amide group (Fig. 2a). Fig. 2b and c illustrate an experiment that can be used to measure the effect of this interaction on the H-bond donor properties of the amide. In Fig. 2b, the association constant (*K*) measures the intermolecular H-bonding interaction of the amide with a phosphine oxide. Fig. 2c shows the corresponding equilibrium for the interaction of a phosphine oxide with a reference amide which does not have an intramolecular H-bond (*K*′). The effect of the intramolecular H-bond on the H-bond donor properties of the amide NH group can be quantified by measuring the ratio of the two association constants (*K*/*K*′). The X substituent provides a handle that can be used to tune the H-bond donor properties of the phenol OH group, in order to investigate whether there is a relationship between the strengths of the intramolecular and intermolecular H-bonds.

## Results

### Intramolecular H-bonding interactions

**Fig. 3:**
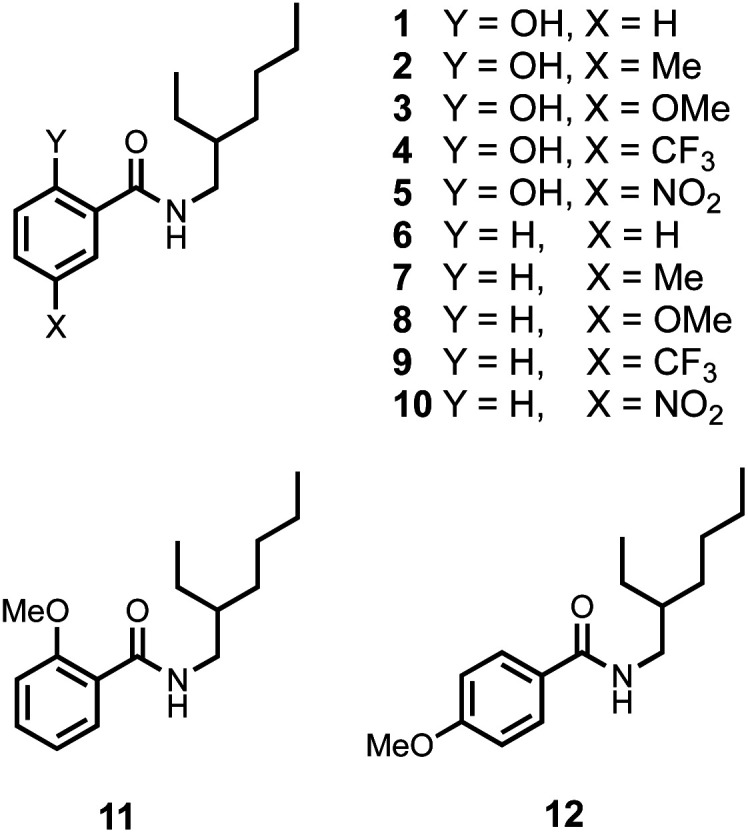
Chemical structures of compounds 1–12.

The compounds shown in Fig. 3 were synthesised from the corresponding carboxylic acids in one step using racemic 2-ethylhexylamine (see ESI†). The use of a racemic branched alkyl chain ensures good solubility in non-polar solvents. In order to establish that the intramolecular H-bonding interactions observed in the CSD persist in solution, ^1^H NMR spectra were recorded in CDCl_3_. Table 1 compares the chemical shift of the signal due to the OH proton in compounds 1–5 with the chemical shift of the signal due to the OH proton in the corresponding para-substituted phenols (4-X-phenol in Table 1).^18,19^ In all cases, the presence of the amide group leads to an increase of about 7 ppm in chemical shift compared with the simple phenol, which indicates that the OH proton is involved in an intramolecular H-bond in compounds 1–5.

**Table 1:** ^1^H NMR chemical shifts (ppm) measured in CDCl_3_ solution at 298 K

X	*δ*(NH)		*δ*(OH)	
	Y = OH	Y = H	Y = OH	4-X-phenol^a^
H	6.24	6.06	12.38	5.10
Me	6.24	6.06	12.16	4.79
OMe	6.19	6.11	11.80	4.82
CF_3_	6.31	6.12	12.85	5.60
NO_2_	6.43	6.19	13.45	6.20

a Ref. 18 and 19.

**Fig. 4:**
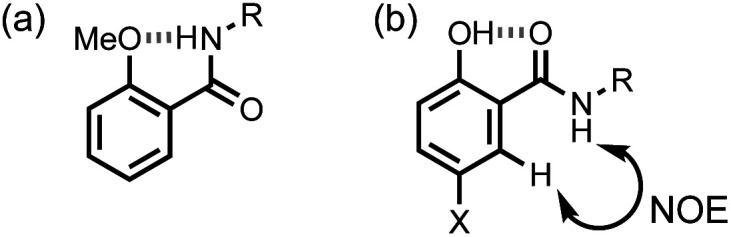
(a) Intramolecular H-bond in compound 11. (b) Cross peak observed in the NOESY spectrum of compound 5 in deuterochloroform at 298 K.

It is also possible that the amide NH proton could make an intramolecular H-bond with the phenol oxygen atom. However, the chemical shift of the signal due to the amide NH proton in compounds 1–5 is very similar to the chemical shift in the compounds 6–10, which do not have the phenol OH group (Y = H in Table 1). In compound 11, the Y hydroxyl group is replaced by a methoxy group, and the chemical shift of the signal due to the amide NH proton in this compound is 7.87 ppm. The large increase in chemical shift compared with the values in Table 1 indicates that there is an intramolecular H-bond between the amide NH proton and the methoxy oxygen atom in compound 11 (Fig. 4a) and confirms that this interaction is not present in compounds 1–5.

In summary, the chemical shift data in Table 1 indicate that in solution compounds 1-5 maintain the conformation shown in Fig. 2a: the amide carbonyl oxygen is H-bonded to the phenol OH, and the amide NH does not make an intramolecular interaction. NOE experiments carried out on compound 5 support this conclusion: an NOE was observed between the amide NH and *ortho* CH proton (Fig. 4b, see ESI†).

### Intermolecular H-bonding interactions

**Fig. 5:**
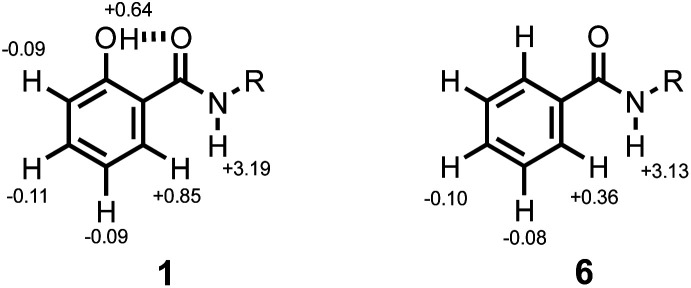
Limiting complexation-induced changes in ^1^H NMR chemical shift (ppm) for formation of 1 : 1 complexes with tri-*n*-butyl phosphine oxide in *n*-octane at 298 K.

^1^H NMR titrations were carried out to establish the mode of interaction with a phosphine oxide in *n*-octane solution. Addition of tri-*n*-butyl phosphine oxide to compound 1 led to a large increase in the chemical shift of the signal due to the amide NH proton. The titration data fit well to a 1 : 1 binding isotherm (see ESI†), and the limiting complexation-induced changes in chemical shift for the 1 : 1 complex are shown in Fig. 5. The corresponding data for titration of tri-*n*-butyl phosphine oxide into compound 6 are shown for comparison. In both cases, there is an increase of over 3 ppm in the chemical shift of the signal due to the amide NH proton, which is indicative of formation of a H-bond at this site. The second largest change in chemical shift was observed for the CH proton *ortho* to the amide group, which indicates close proximity to the phosphine oxide oxygen atom in the complex. The change in chemical shift for the signal due to the phenol OH proton is significantly smaller. These results indicate that the phenol does not compete with the amide-phosphine oxide H-bond and that the phenol-amide H-bond remains intact in the 1 : 1 complex as shown in Fig. 2b. The intramolecular NOE illustrated in Fig. 4b is also observed for compound 5 recorded in the presence of an excess of tri-*n*-butyl phosphine oxide, which confirms that there is no change in conformation on binding (see ESI†).

**Fig. 6:**
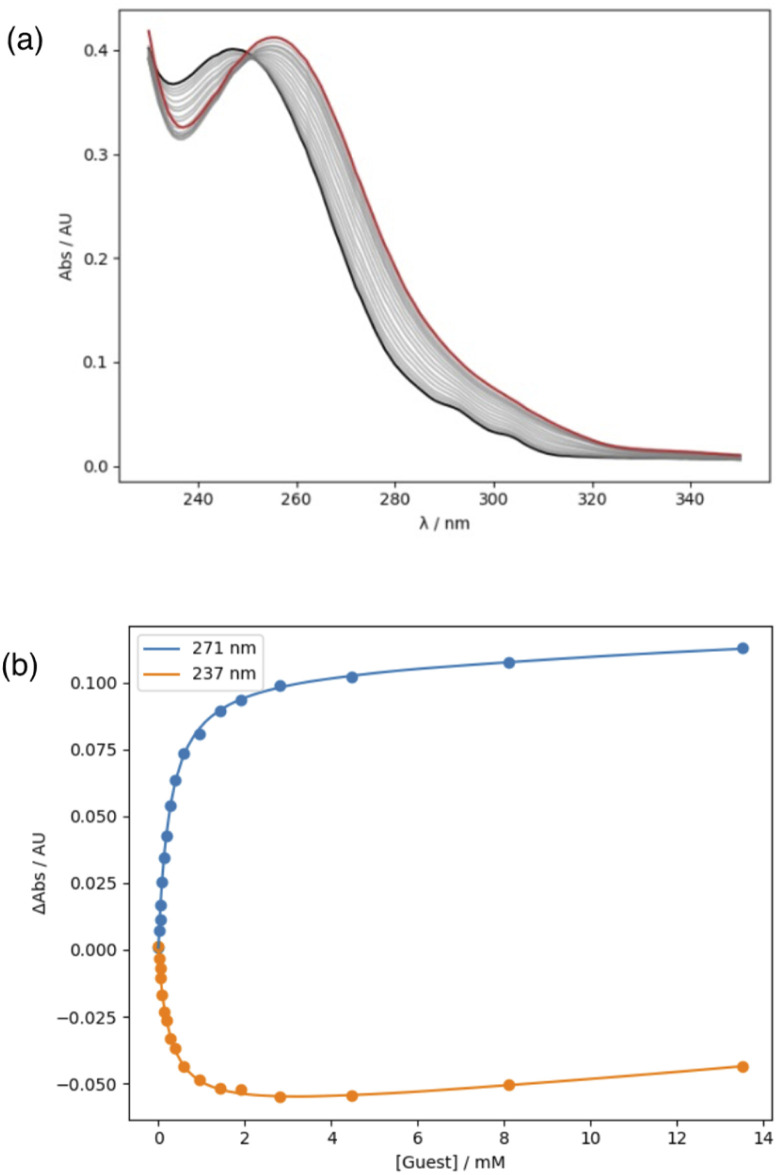
(a) UV/Vis absorption spectra for titration of tri-*n*-butyl phosphine oxide (guest) into 10 in *n*-octane at 298 K. The initial spectrum is shown in black, and the final spectrum in red. (b) Changes in absorbance at 271 and 237 nm are shown as points, and the lines are the best fit to a 1 : 1 binding isotherm.

Association constants for the interaction of compounds 1–10 with tri-*n*-butyl phosphine oxide were measured using UV/Vis absorption titrations in *n*-octane. Dilution experiments indicated that there is no self-association of the amides in *n*-octane at the concentrations used for titrations. A red-shift was observed in the wavelength of the absorption maximum on addition of the phosphine oxide, and the titration data fit well to a 1 : 1 binding isotherm in all cases (Fig. 6, see ESI†). The results are summarised in Table 2. Comparison of the association constants for compounds 1–5 with the corresponding values for compounds 6–10 shows that the presence of the phenol OH group leads to an increase in binding affinity. The ratios of association constants for compounds with the same X substituent (*K*/*K*′) are all much greater than one, which suggests that the intramolecular H-bond has a significant effect on the strength of the intermolecular H-bond between the amide NH and the phosphine oxide.

**Table 2:** Association constants (M^−1^) for formation of 1 : 1 complexes with tri-*n*-butyl phosphine oxide in *n*-octane at 298 K^a^

X	*K*(Y = OH)	*K*′(Y = H)	*K*/*K*′
H	940 ± 140	202 ± 17	4.7
Me	650 ± 140	120 ± 20	5.4
OMe	1800 ± 300	220 ± 140	8.2
CF_3_	8790 ± 60	1030 ± 50	8.5
NO_2_	46 000 ± 8000	3840 ± 90	12.0

a Average values from at least 3 experiments with errors at the 95% confidence limit.

In addition to the through space H-bonding interaction, the phenol oxygen atom is also directly conjugated to the amide group, and through bond effects could be responsible for the observed change in the H-bond donor properties of the amide NH. In order to obtain insight into the magnitude of this effect, titrations were carried out using compounds 11 and 12. No binding could be detected for compound 11, because the intramolecular H-bond shown in Fig. 4a competes effectively with intermolecular interactions. Although the methoxy group is para to the amide in compound 12, the electronic effect on the amide group should be qualitatively similar to an *ortho* substituent. The association constant for the 12·phosphine oxide complex is 170 ± 70 M^−1^ in *n*-octane, which is very similar to the value measured for the unsubstituted parent compound 6 (202 ± 17 M^−1^). In other words, through bond polarisation is unlikely to be the cause of the large changes in association constant associated with the presence of phenol OH group.

## Discussion

**Fig. 7:**
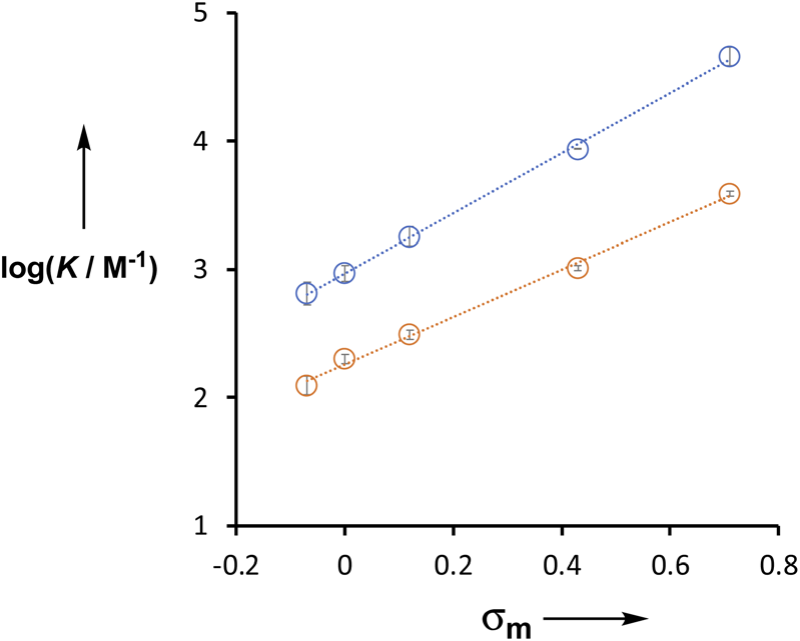
Relationship between the association constants for formation of 1 : 1 complexes with tri-*n*-butyl phosphine oxide in *n*-octane at 298 K (log *K*) and the Hammett parameter *σ*_m_ for substituent X. Compounds 1–5 are shown in blue (Y = OH), and the equation of the line is *y* = 2.35*x* + 2.97. Compounds 6–10 are shown in orange (Y = H), and the equation of the line is *y* = 1.89*x* + 2.21.

The X substituent has a large effect on the H-bond donor properties of the amide group. For compounds 6–10, which do not have the phenol OH group (Y = H in Table 2), the association constants span an order of magnitude. The X substituent is *meta* to the amide group, and the association constants for both series of compounds (Y = H and OH) correlate well with the Hammett substituent constant, *σ*_m_ (Fig. 7).^20^ For the Y = H series, the slope of the line of best fit (*i.e.* the Hammett *ρ* value) is +1.9, but for the Y = OH series, the slope is +2.4. This result indicates that the X substituent exerts an influence on the H-bonding properties of the amide by two different mechanisms. There is direct through bond polarisation of the amide group, which contributes +1.9 to the *ρ* value, and through space polarisation mediated by the H-bonding interaction with the phenol OH group, which increases the *ρ* value. The positive *ρ* value indicates that electron-withdrawing substituents increase the H-bond donor ability of the amide NH group. Electron-withdrawing groups also increase the polarity of the phenol OH, so the increased *ρ* value for Y = OH suggests that increasing the strength of the intramolecular H-bond leads to an increase in the polarity of the amide NH group and an increase in the strength of the intermolecular H-bond.

The H-bonding properties of a functional group can be quantified using the parameter *α* for H-bond donors or *β* for H-bond acceptors. If the corresponding solvent parameters, *α*_s_ and *β*_s_, are known, then the association constant for formation of a H-bonded complex between two solutes (*K*) can be estimated using eqn (1).^1^

1 Δ*G°*/kJ mol^−1^ = −*RT*ln *K* = −(*α* − *α*_s_)(*β* − *β*_s_) + 6

**Fig. 8:**
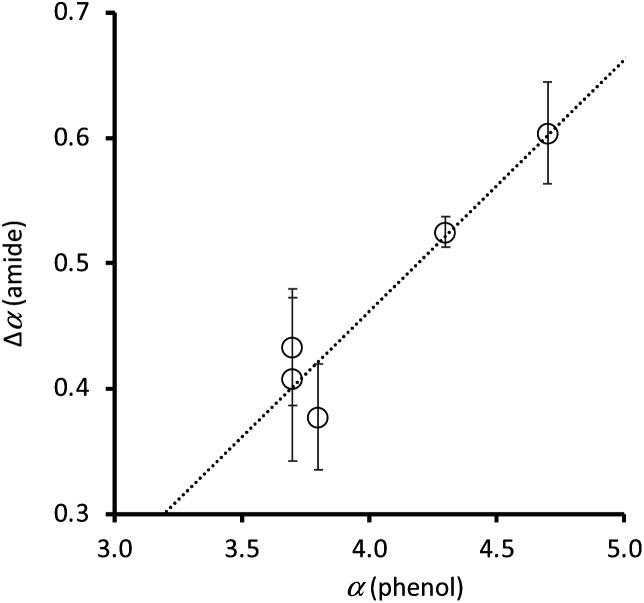
Relationship between the H-bond donor properties of 4-X-phenols, *α*(phenol), and the change in H-bond donor properties of the amide group in compounds 1–5 due to the intramolecular H-bond, Δ*α*(amide). The best fit line is *y* = 0.20*x* − 0.34.

The value of *β* for a phosphine oxide^21^ and the H-bond parameters for alkane solvents^22^ have been experimentally determined, so the association constants in Table 2 can be used in eqn (1) to determine the H-bond donor parameter *α* for compounds 1–10. Table 3 shows that the phenol OH group leads to a significant increase in the H-bond donor parameter of the amide NH group in compounds 1–5. The H-bond donor parameters for the corresponding para-substituted phenols (4-X-phenol) are also listed in Table 3.^23^Fig. 8 compares the increase in the value of *α* measured for the amide NH group due to the presence of the intramolecular H-bond (Δ*α*(amide) = *α*(Y = OH) − *α*(Y = H)), with the value of *α* measured for the corresponding para-substituted phenol (*α*(phenol)). There is a clear correlation, which indicates that through space polarisation mediated by the intramolecular H-bond increases the polarity of the amide NH group in proportion to the polarity of the phenol OH group.

**Table 3:** H-bond donor parameters (*α*)

X	Y = H^a^	Y = OH^a^	4-X-phenol^b^
H	3.1	3.5	3.7
Me	3.0	3.4	3.8
OMe	3.1	3.6	3.7
CF_3_	3.5	4.0	4.3
NO_2_	3.8	4.4	4.7

a Calculated using eqn (1) with *β* = 10.7, *α*_s_ = 1.2 and *β*_s_ = 0.6.

b Ref. 23.

**Fig. 9:**
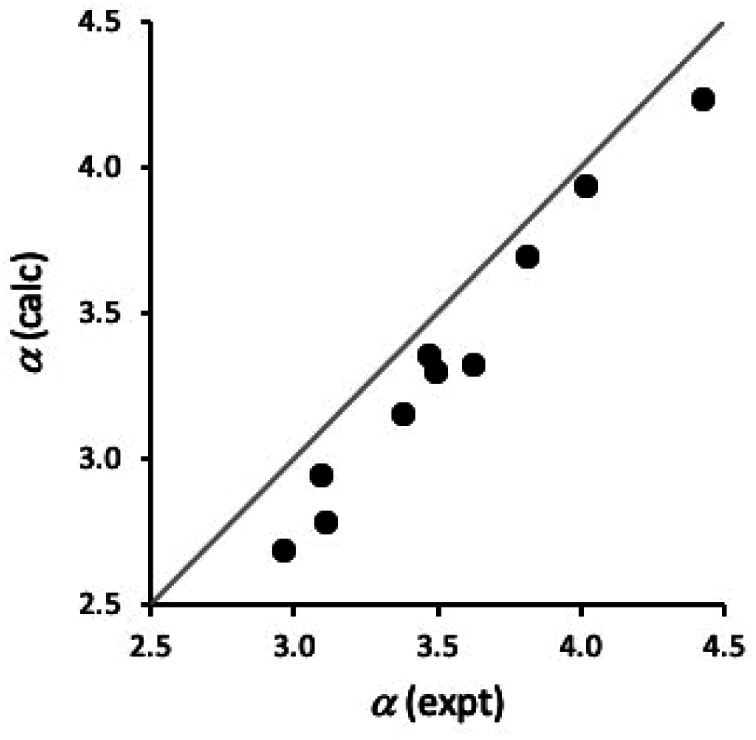
Comparison of calculated and experimental values of *α* for compounds 1–10. The line is *y* = *x*.

It is also possible to estimate H-bond donor parameters from the maximum in the molecular electrostatic potential calculated on the van der Waals surface using density functional theory for isolated molecules in the gas phase (see ESI†).^24^Fig. 9 compares calculated values of *α* for compounds 1–10 with the experimental values in Table 3. Although the calculated values are underestimated by about 0.2, there is a good correlation between the calculated and experimental values (*R*^2^ = 0.98). This result suggests that *ab initio* calculations could provide a good description of substituent effects on both through bond polarisation and through space polarisation due to the intramolecular H-bonding interactions in compounds 1–5. The underestimate in the calculated value of *α* is similar for compounds with and without intramolecular H-bonds, which suggests that the discrepancy is an intrinsic property of the benzamide group. The complexation-induced changes in the chemical shift shown in Fig. 5 suggest that there is a CH⋯O H-bond between the phosphine oxide oxygen and the CH group *ortho* to the amide, and this secondary interaction would stabilise the complex in *n*-octane, increasing the experimentally measured value of *α*.

## Conclusions

The systems describe here allow direct measurement of the effects of polarisation on the H-bond donor properties of secondary amides. In 2-hydroxylbenzamides, there is an intramolecular H-bond between the phenol OH and the amide carbonyl oxygen in the solid state, in chloroform solution and in *n*-octane solution. This H-bond is maintained when the amide NH group makes an intermolecular H-bond with a phosphine oxide. Comparison of the association constant measured for the phosphine oxide complex with the value measured for the corresponding benzamide, which lacks the phenol OH group, can be used to quantify the polarisation effect due to the intramolecular H-bond. Substituents attached to the aromatic ring modulate the polarity of the amide NH group, due polarisation *via* the bonding framework. These substituents also change the polarity of the phenol OH group, which modulates the through space polarisation of the amide NH group due to the intramolecular H-bond. Although it is difficult to completely separate the effects of through bond and through space polarisation on the amide, the experiments described here allow estimates of the relative magnitudes of the two effects. There is a direct correspondence between substituent effects on the H-bond donor properties of the phenol OH group and the concomitant effect of through space polarisation on the H-bond donor parameter of the amide group. In other words, the strength of the intramolecular H-bond directly modulates the strength of the intermolecular H-bond made by the amide group. This result suggests that there may be simple relationships that allow the prediction of polarisation effects on different functional groups and further studies are underway in our laboratory to investigate this phenomenon.

## Data availability

All supporting data is provided in the ESI.†

## Author contributions

DOS, FEH and MCM carried out the experiments, and all authors contributed to writing the manuscript.

## Conflicts of interest

There are no conflicts to declare.

## Supplementary Material

SC-013-D2SC04271A-s001
