# Supplementary material for: H-bond cooperativity: polarisation effects on secondary amides
Source: Chem Sci. 2022 Oct 3;13(40):11863–8. doi: 10.1039/d2sc04271a (PMC9580511; doi:10.1039/d2sc04271a)
Supplement: SC-013-D2SC04271A-s001 [file SC-013-D2SC04271A-s001.pdf]

Electronic Supplementary Material (ESI) for Chemical Science.
This journal is © The Royal Society of Chemistry 2022

H-bond Cooperativity: Polarisation Effects on Secondary Amides

Daniil O. Soloviev, Fergal E. Hanna, M. Cristina Misuraca and Christopher A. Hunter *

Yusuf Hamied Department of Chemistry, University of Cambridge, Lensfield Road, Cambridge CB2 1EW (UK).

Email: herchelsmith.orgchem@ch.cam.ac.uk

Supplementary Information

Table of Contents

Materials and methods 2

Synthesis and characterisation 3

UV/Vis absorption spectroscopy titrations 27

1D NOE difference spectra 39

^{1}H NMR titrations 40

Computational analysis 47

References 48

Materials and methods

All reagents were purchased from commercial sources (Sigma Aldrich UK, Acros, Tokyo Chemical Industry, Alfa Aesar and FluoroChem) and were used as received without any further purification unless stated. Dry solvents were obtained by means of a Grubbs solvent purification system.

Flash chromatography was done with an automated system (Combiflash Companion) using pre-packed cartridges of silica (50 µm PuriFlash® column) or basic alumina (45 µm PuriFlash® column)

The LC-MS analysis of samples was performed using Waters Acquity H-class UPLC coupled with a single quadrupole Waters SQD2. ACQUITY UPLC CSH C18 Column, 130 Å, 1.7 µm, 2.1 mm X 50 mm was used as the UPLC column for all samples. The conditions of the UPLC method are as follows: Solvent A: Water +0.1% Formic acid; Solvent B: Acetonitrile +0.1% Formic acid; Gradient of 0-2 minutes 5% - 100%B + 1 minute 100% B with re-equilibration time of 2 minutes. Flow rate: 0.6 ml/min; column temperature of 40°C; injection volume of 2 µL. The signal was monitored with MS-ES+, MS-ES-, and UV/Vis absorption at 254 nm or at 290 nm.

^{1}H-NMR and ^{13}C-NMR were recorded on a 400 MHz Bruker spectrometer. $CDCl_3$ was dried over 4 Å molecular sieves to minimise water content. The reference values used for the chemical shifts of the various spectra are reported in the literature[54]. The splitting pattern is indicated with the following abbreviations: s for singlet, d for doublet, t for triplet, q for quartet, p for pentet and m for multiplet.

FT-IR spectra were collected with a Bruker ALPHA FT-IR Spectrometer.

UV/Vis spectra were recorded with an Agilent UV/Vis Cary 60 spectrophotometer.

Melting points were measured in a Mettler Toledo MP50 Melting Point System.

Synthesis and characterisation

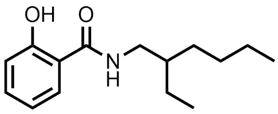

N-(2-ethylhexyl)-2-hydroxybenzamide, **1**. Salicylic acid (0.3829 g, 2.772 mmol), *N*-(3-dimethylaminopropyl)-*N*'-ethylcarbodiimide hydrochloride (0.6853 g, 3.929 mmol), and 4-dimethylamino pyridine (0.3768 g, 3.084 mmol) were combined in a flask. The flask was flushed with nitrogen, and 30 ml dry THF was added, followed by 2-ethylhexylamine (0.46 ml, 2.8 mmol). The reaction mixture was stirred for 48 hours at room temperature under a slight positive pressure of nitrogen.

The resulting mixture was filtered under gravity. DCM (30 ml) was added, and the combined organic layer was washed with brine (3x50 ml), dried with MgSO4, and filtered under gravity again. The crude product (1.20 g) was purified by flash chromatography (DCM/PE on SiO_2) to yield the pure product **1** as a faintly yellow oil (0.457 g, 1.834 mmol, 65.3%).

FT-IR: ν_{max} (cm^{-1}) 3365 (N-H), 2958 (C-H), 2927 (C-H), 2858 (C-H), 1637 (C=O), 1591, 1541, 1491, 1454, 1362, 1303, 1252, 1233

^1H NMR (400 MHz, Chloroform-*d*) δ 12.38 (s, 1H), 7.39 (t, *J* = 7.8 Hz, 1H), 7.32 (d, *J* = 8.0 Hz, 1H), 6.99 (d, *J* = 8.3 Hz, 1H), 6.85 (t, *J* = 7.6 Hz, 1H), 6.24 (s, 1H), 3.51 – 3.33 (m, 2H), 1.59 (p, *J* = 6.6 Hz, 1H), 1.39 (p, *J* = 8.2, 7.6 Hz, 2H), 1.33 (d, *J* = 4.1 Hz, 6H), 1.00 – 0.82 (m, 6H).

^{13}C NMR (101 MHz, $CDCl_3$) δ 169.93, 161.55, 134.07, 125.03, 118.67, 118.54, 114.41, 77.32, 77.00, 76.68, 42.48, 39.38, 31.06, 28.86, 24.32, 22.99, 14.04, 10.88.

HRMS (ES+): Calculated for $C_{15}H_{24}NO_2^+$ 250.1807, found 250.1800

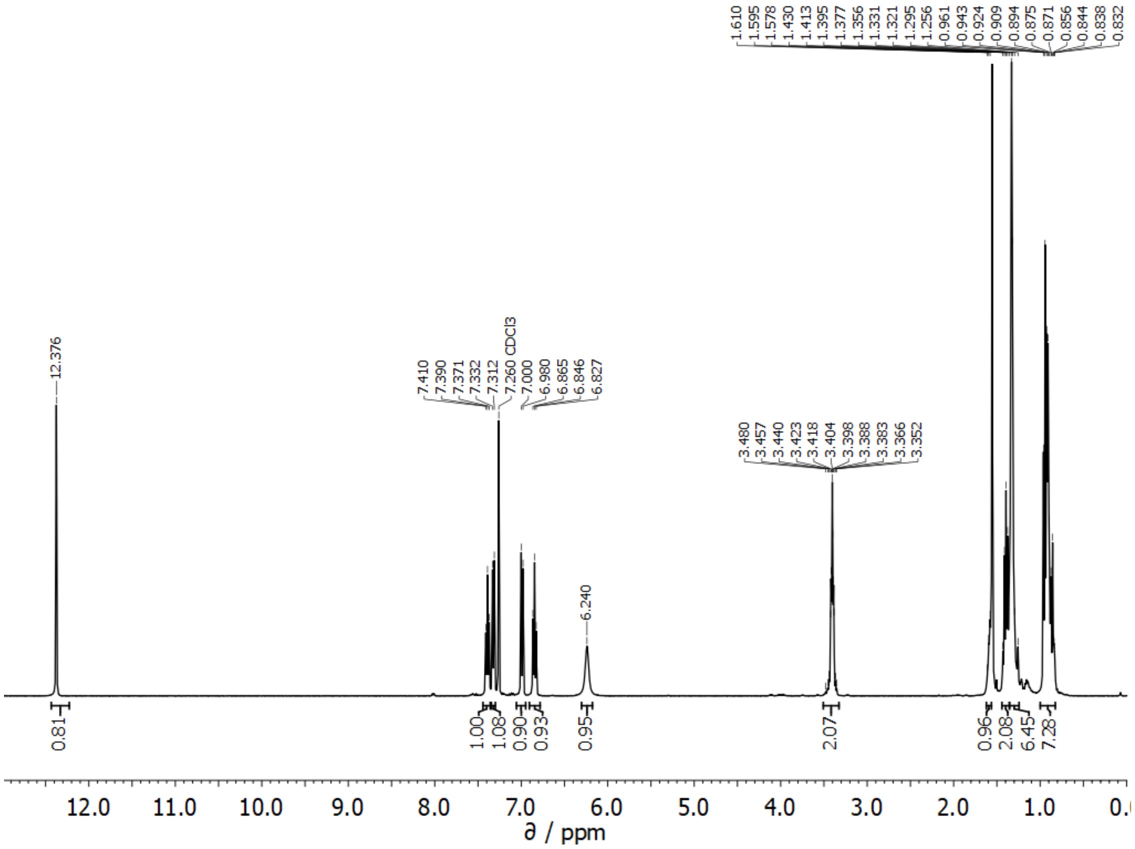

Figure S.1 – ^{1}H NMR spectrum of **1** in $CDCl_3$ (δ 0 – 13 ppm).

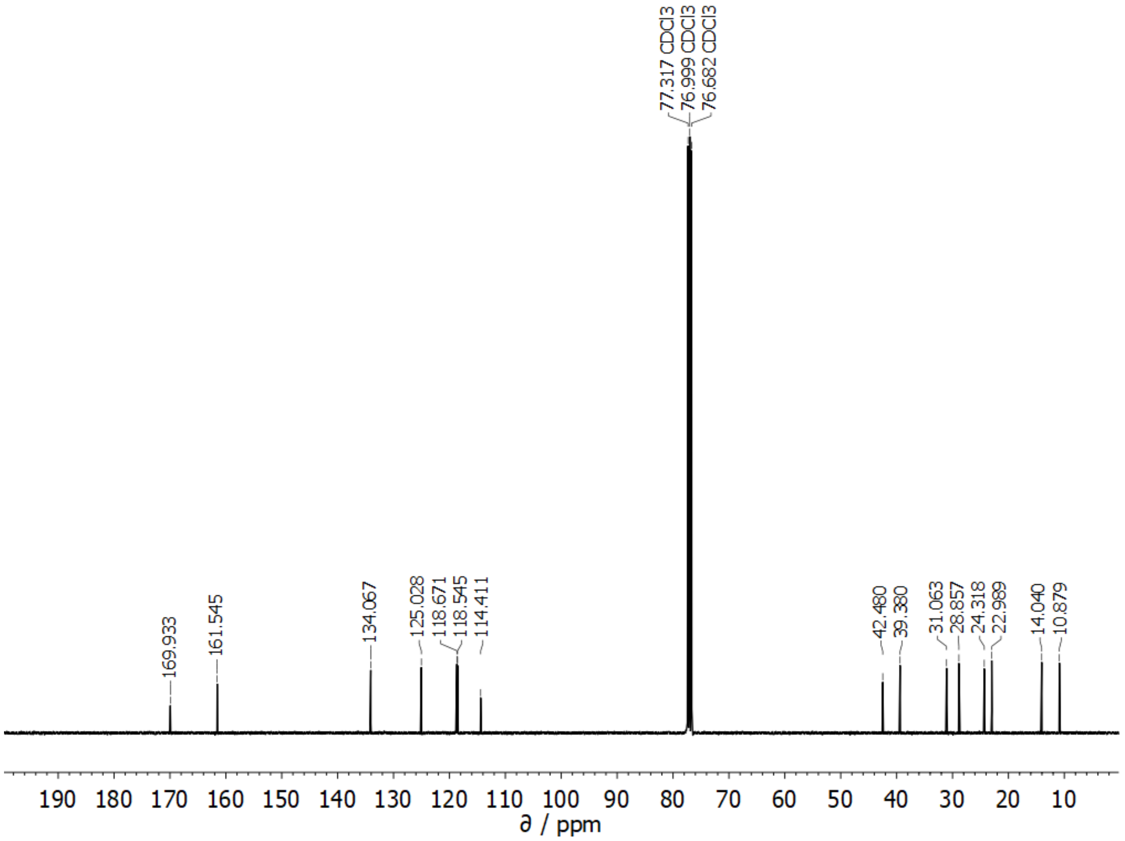

Figure S.2 – ^{13}C NMR spectrum of **1** in $CDCl_3$ (δ 0 – 200 ppm).

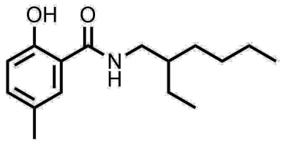

N-(2-ethylhexyl)-2-hydroxy-5-methylbenzamide, **2**. 5-methylsalicylic acid (0.497 g, 3.28 mmol), EDC hydrochloride (0.661, 3.45 mmol) and DMAP (0.365 g, 3.25 mmol) were placed under a N_2 atmosphere and dissolved in dry THF (30 mL). 2-ethylhexylamine (0.58 mL, 3.5 mmol) was added and the mixture stirred at room temperature for 24 hours, then heated to 50 ^{O}C for 3 hours. The reaction mixture was diluted in DCM (30 mL), washed with HCl (1 M, 25 mL) and saturated $NaHCO_3$ (25 mL). The organic layer was dried with $MgSO_4$ and the solvent removed in vacuo. The crude was purified by flash chromatography (PE/EtOAc on SiO_2) to yield the desired product as a clear oil (0.437 g, 1.66 mmol, 51%).

ν_{max} (film) cm^{-1} : 3362 (N-H), 2959 (C-H), 2926 (C-H), 2872 (C-H), 1642 (C=O), 1588 (C=C), 1544 (C=C), 1499, 1462, 1364, 1296, 1237, 821

^{1}H NMR (400 MHz, $CDCl_3$) δ_H ppm: 12.17 (s, 1H), 7.20 (d, J = 8.5 Hz, 1H), 7.09 (s, 1H), 6.89 (dd, J = 8.4, 1.9 Hz, 1H), 6.24 (s, 1H), 3.39 (t, J = 6.4 Hz, 2H), 2.29 (d, J = 1.9 Hz, 3H), 1.57 (q, J = 6.3, 5.7 Hz, 1H), 1.40 (q, J = 7.3, 6.8 Hz, 2H), 1.33 (d, J = 3.9 Hz, 8H), 0.97-0.88 (m, 7H).

^{13}C{^{1}H} NMR (101 MHz, $CDCl_3$) δ_C ppm: 170.13, 159.50, 135.11, 127.79, 125.16, 118.58, 114.18, 42.66, 39.57, 31.21, 29.03, 24.47, 23.15, 20.73, 14.21, 11.03.

HRMS: calc for $C_{16}H_{26}NO_2^+$ [M+H]$^+$: 264.1964, found: 264.1960

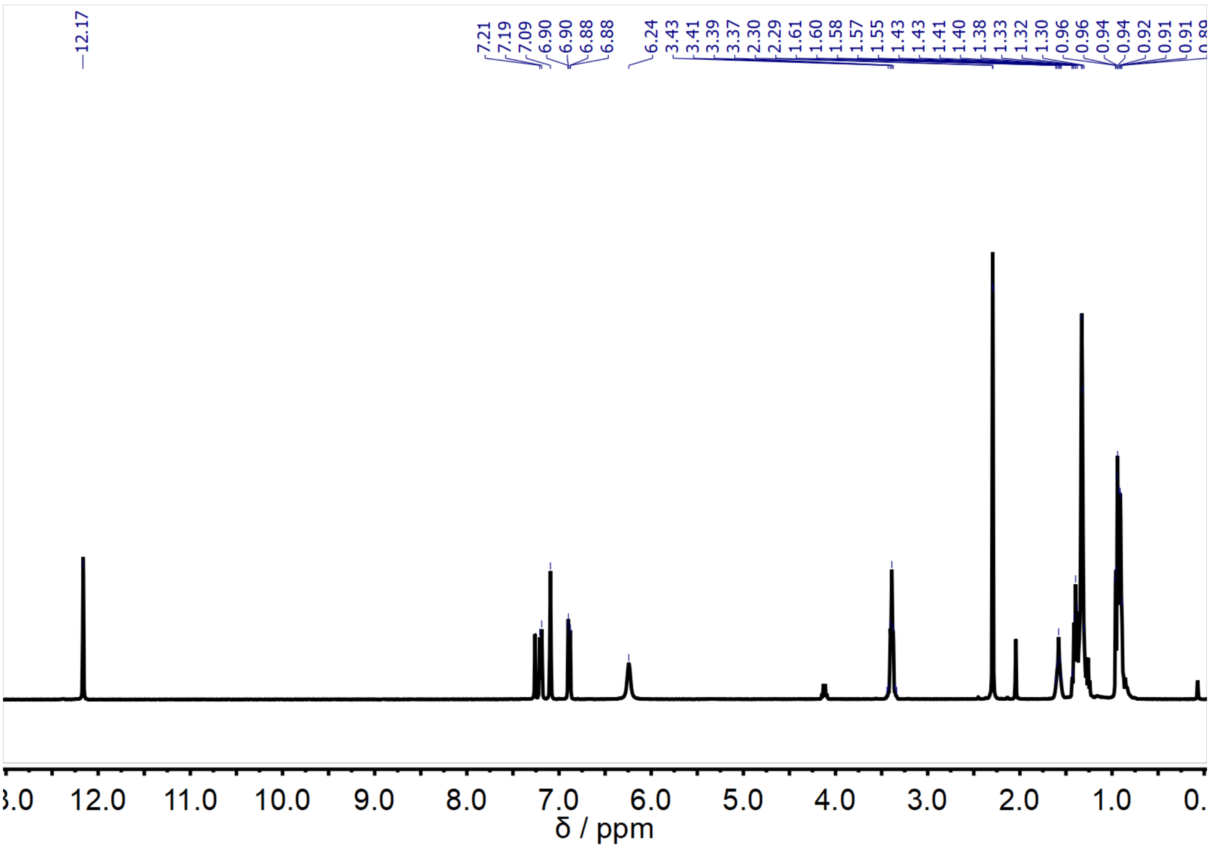

Figure S.3 – ^{1}H NMR spectrum of **2** in $CDCl_3$ (δ 0 – 13 ppm).

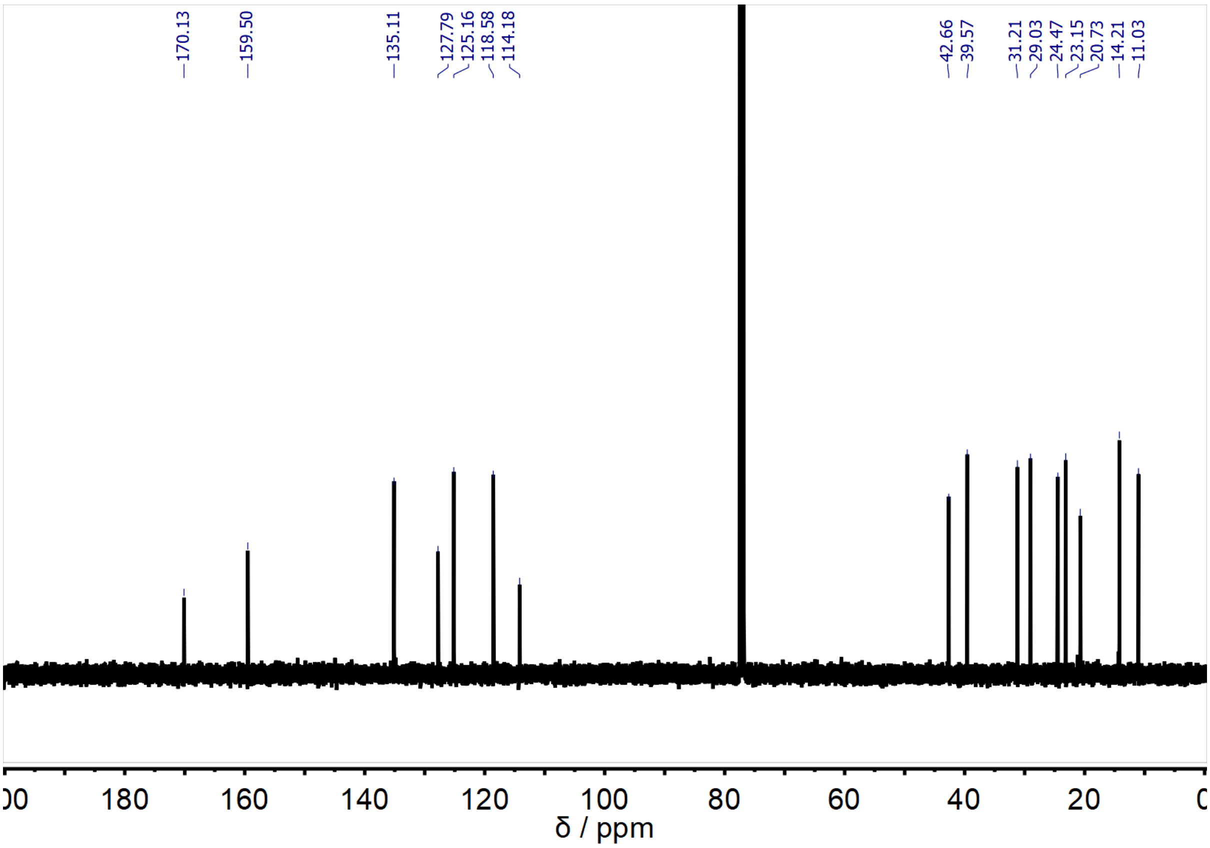

Figure S.4 – ^{13}C NMR spectrum of **2** in $CDCl_3$ (δ 0 – 200 ppm).

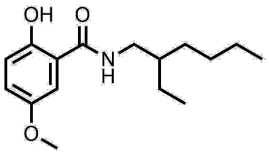

N-(2-ethylhexyl)-2-hydroxy-5-methoxybenzamide, **3**. 5-methoxysalicylic acid (0.516 g, 3.07 mmol), EDC hydrochloride (0.649 g, 3.33 mmol) and DMAP (0.380 g, 3.39 mmol) were placed under a N_2 atmosphere and dissolved in dry THF (30 mL). 2-ethylhexylamine (0.58 mL, 3.5 mmol) was added and the mixture stirred at room temperature for 24 hours, then heated to 50 ^{O}C for 3 hours. The reaction mixture was diluted in DCM (30 mL), washed with HCl (1 M, 25 mL) and saturated $NaHCO_3$ (25 mL). The organic layer was dried with $MgSO_4$ and the solvent removed in vacuo. The crude was purified by flash chromatography (PE/EtOAc on SiO_2) to yield the desired product as a clear oil (0.525 g, 1.88 mmol, 61%).

ν_{max} (film) cm^{-1} : 3353 (N-H), 2958 (C-H), 2927 (C-H), 2872 (C-H), 2859 (C-H), 1637 (C=O), 1589 (C=C), 1546 (C=C), 1494, 1464, 1424, 1364, 1288, 1237, 1204, 1041, 824

^{1}H NMR (400 MHz, $CDCl_3$) δ_H ppm: 1H NMR (400 MHz, Chloroform-d) δ 11.80 (s, 1H), 7.02 (d, J = 9.4 Hz, 1H), 6.93 (d, J = 9.0 Hz, 1H), 6.81 (d, J = 2.5 Hz, 1H), 3.79 (d, J = 2.1 Hz, 3H), 3.39 (t, J = 6.3 Hz, 2H), 1.57 (h, J = 6.2 Hz, 1H), 1.43 – 1.35 (m, 2H), 1.34 – 1.29 (m, 5H), 0.96 – 0.88 (m, 6H).

^{13}C{^{1}H} NMR (101 MHz, $CDCl_3$) δ_C ppm:.78, 155.67, 151.82, 120.38, 119.41, 114.58, 110.28, 56.27, 42.74, 39.54, 31.22, 29.03, 24.49, 23.15, 14.21, 11.04.

HRMS: calc for $C_{16}H_{26}NO_3^+$ [M+H]$^+$: 280.1913, found: 280.1916

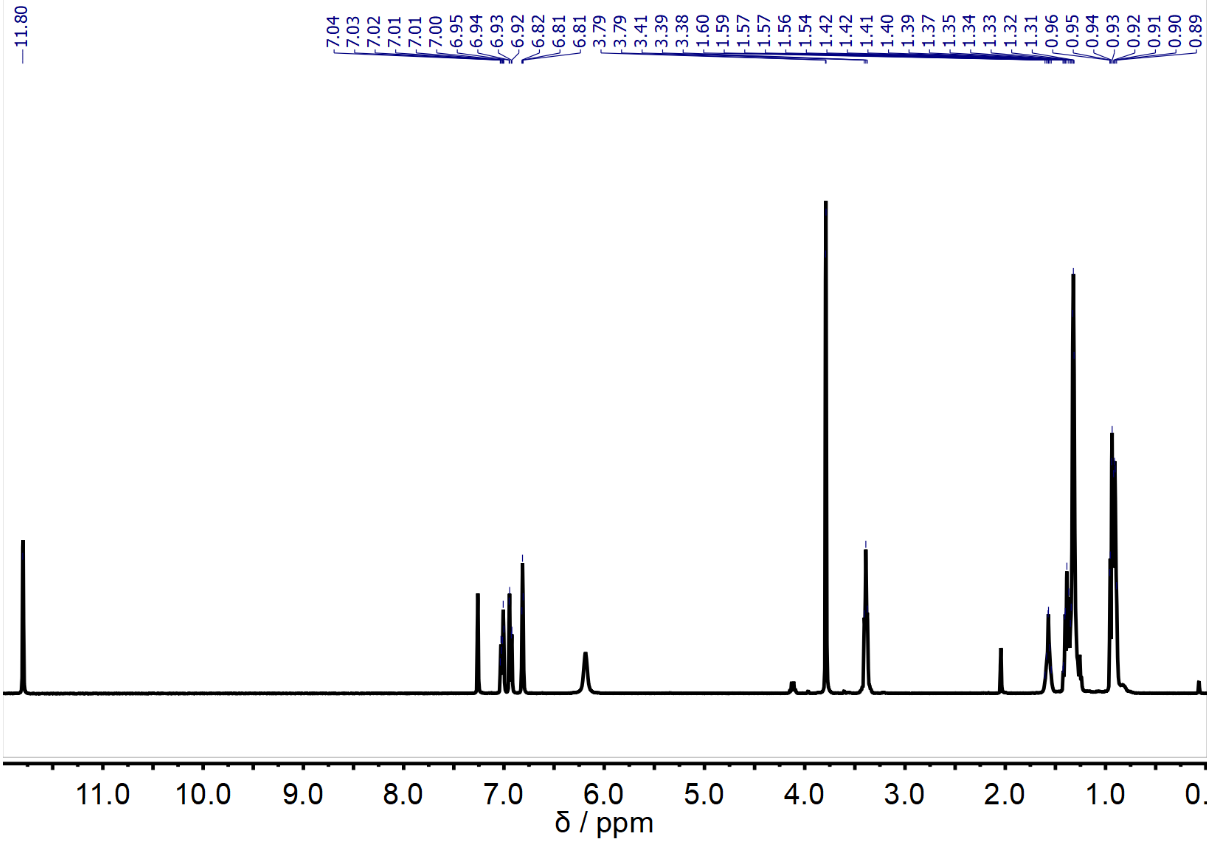

Figure S.5 – ^{1}H NMR spectrum of **3** in $CDCl_3$ (δ 0 – 12 ppm).

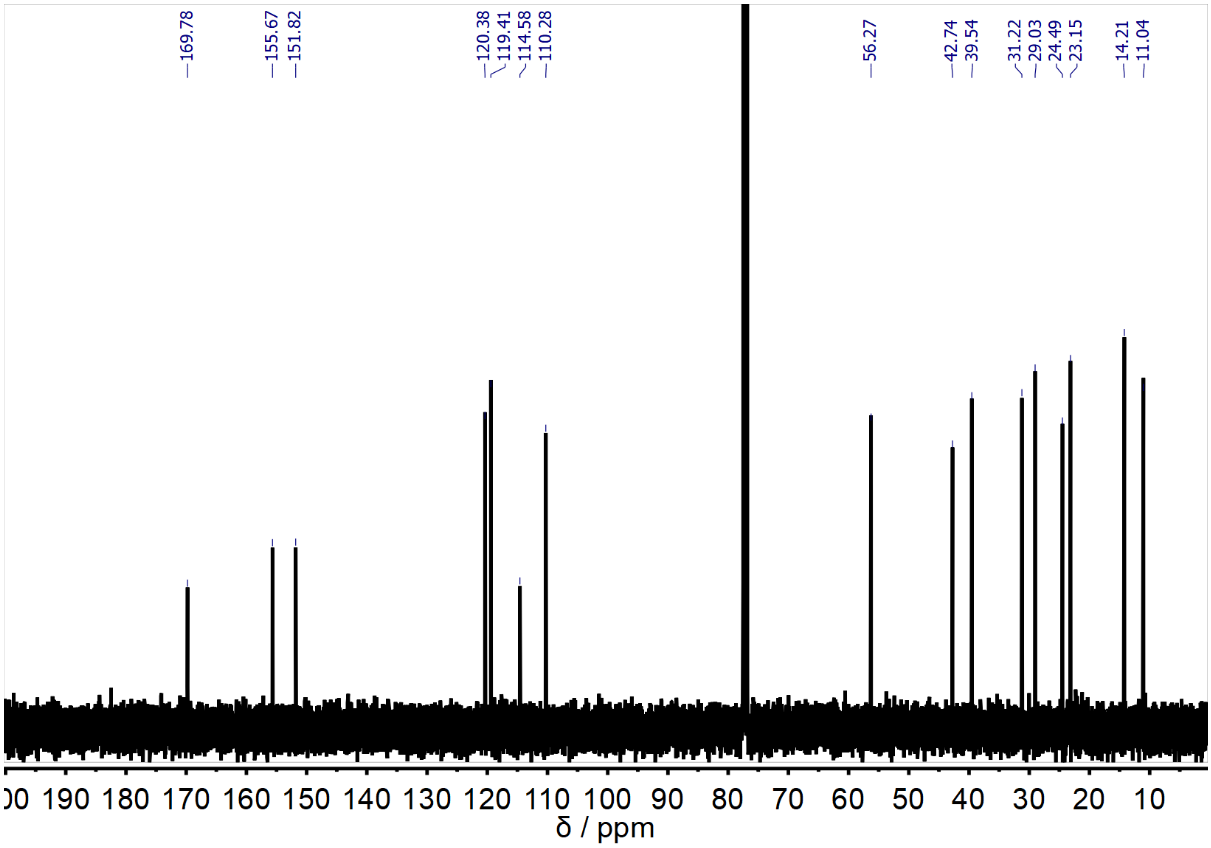

Figure S.6 – ^{13}C NMR spectrum of **3** in $CDCl_3$ (δ 0 – 200 ppm).

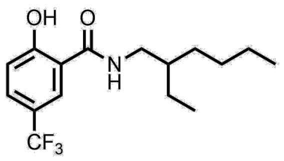

N-(2-ethylhexyl)-2-hydroxy-5-(trifluoromethyl)benzamide, **4**. 5-(trifluoromethyl)salicylic acid (0.111 g, 0.540 mmol), EDC hydrochloride (0.140 g, 0.728 mmol) and DMAP (0.0715 g, 0.637 mmol) were placed under a N_2 atmosphere and dissolved in dry DCM (5 mL). 2-ethylhexylamine (0.10 mL, 0.61 mmol) was added and the mixture stirred at room temperature for 48 hours. The reaction mixture was diluted in DCM (50 mL), washed with HCl (1 M, 2 x 50 mL) and water (3 x 50 mL). The organic layer was dried with $MgSO_4$ and the solvent removed in vacuo. The crude was purified by flash chromatography (PE/EtOAc on SiO_2) to yield the desired product as a clear oil (0.0469 g, 0.148 mmol, 27%).

ν_{max} (film) cm^{-1} : 3379 (N-H), 3084 (O-H), 2961 (C-H), 2930 (C-H), 2874 (C-H), 2861 (C-H), 1645 (C=O), 1592 (C=C), 1553 (C=C), 1317, 1294, 1237, 1155, 1120, 1079, 835, 626, 615

^1H NMR (400 MHz, $CDCl_3$) δ_H ppm: 12.88 (s, 1H), 7.65 (d, J = 8.9 Hz, 1H), 7.60 (s, 1H), 7.10 (d, J = 8.7 Hz, 1H), 6.34 (s, 1H), 3.45 (t, J = 6.3 Hz, 2H), 1.63 (q, J = 5.9 Hz, 1H), 1.44 (q, J = 7.3 Hz, 2H), 1.37 (s, 5H), 1.01 – 0.91 (m, 6H).

$^{13}C\{^1H\}$ NMR (101 MHz, $CDCl_3$) δ_C ppm: 169.18, 164.38, 130.87, 130.84, 125.45, 122.81, 122.77, 121.13, 120.80, 119.50, 114.34, 43.04, 39.53, 31.16, 29.00, 24.44, 23.13, 14.18, 10.97.

HRMS: calc for $C_{16}H_{23}F_3NO_2^+$ $[M+H]^+$: 318.1681, found: 318.1679

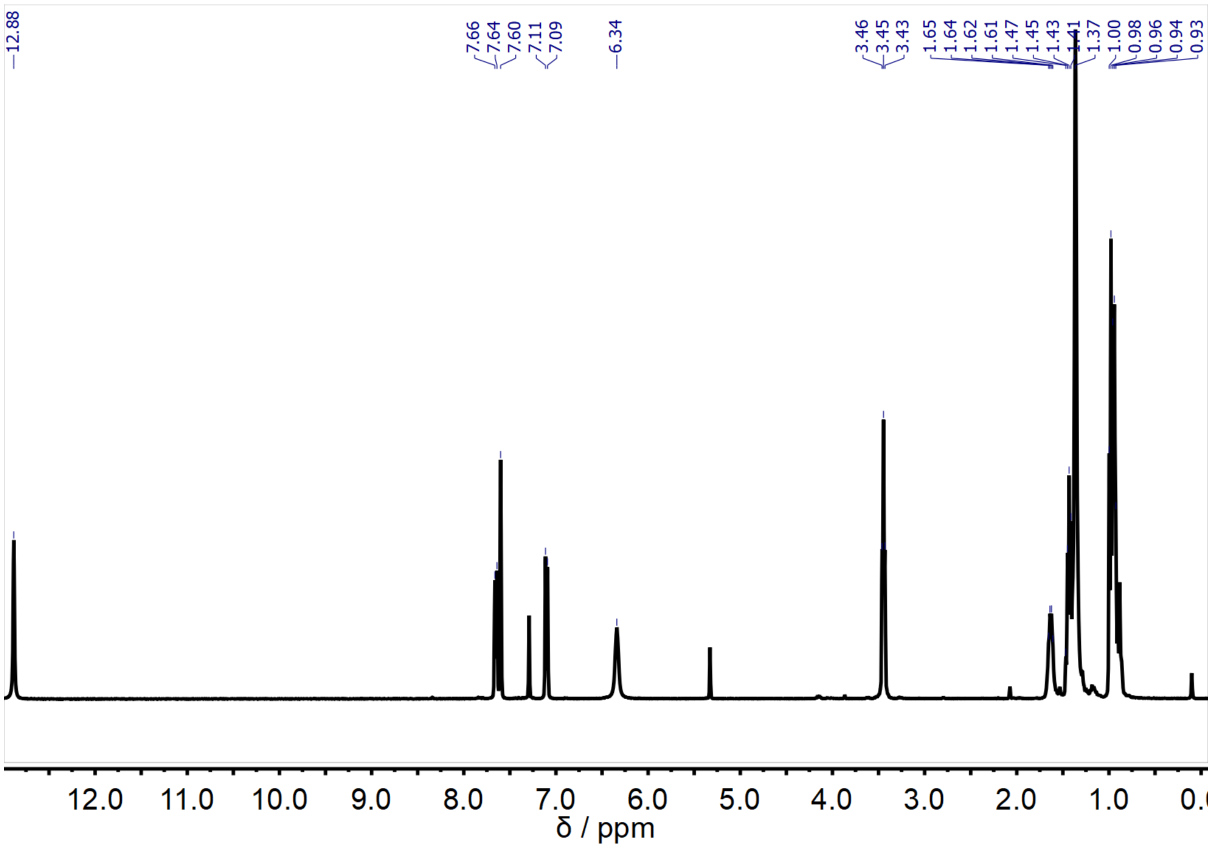

Figure S.7 – ^1H NMR spectrum of **4** in $CDCl_3$ (δ 0 – 10 ppm).

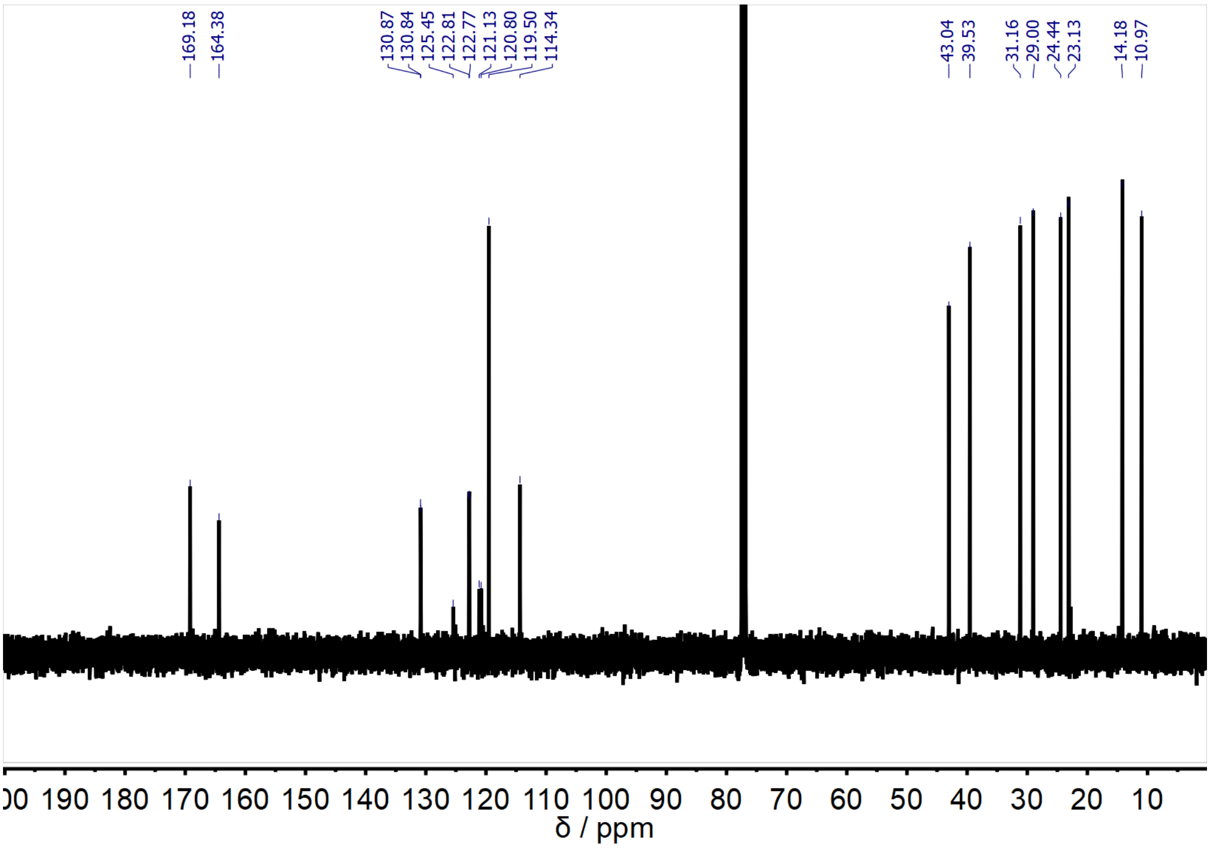

Figure S.8 – ^{13}C NMR spectrum of in $CDCl_3$ (δ 0 – 200 ppm).

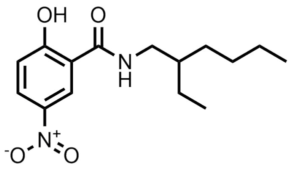

N-(2-ethylhexyl)-2-hydroxy-5-nitrobenzamide, **5**. 5-nitrosalicylic acid (0.4994 g, 2.727 mmol), *N*-(3-dimethylaminopropyl)-*N*'-ethylcarbodiimide hydrochloride (0.6853 g, 3.575 mmol), and 4-dimethylamino pyridine (0.3447 g, 2.822 mmol) were combined in a flask. The flask was flushed with nitrogen, and 30 ml dry THF was added, followed by 2-ethylhexylamine (0.46 ml, 2.8 mmol). The reaction mixture was stirred for 24 hours at room temperature under a slight positive pressure of nitrogen.

The resulting mixture was filtered under gravity. DCM (60 ml) was added, and the combined organic layer was washed with brine (3x50 ml), dried with MgSO4, and filtered under gravity again. The crude product (1.15 g) was purified by flash chromatography (PE/EtOAc on SiO_2) to yield the pure product as a yellow-orange powder (0.2084 g, 0.708 mmol, 26%).

FT-IR: ν_{max} (cm^{-1}) 3398 (N-H), 2959 (C-H), 2928 (C-H), 2872 (C-H), 2859 (C-H), 1651 (C=O), 1599, 1549, 1521, 1490, 1422, 1380, 1335, 1300, 1228, 1153, 1093, 911, 848, 746, 711, 639

^{1}H NMR (400 MHz, Chloroform-*d*) δ 13.45 (s, 1H), 8.35 (d, *J* = 2.6 Hz, 1H), 8.28 (dd, *J* = 9.2, 2.6 Hz, 1H), 7.07 (d, *J* = 9.2 Hz, 1H), 6.43 (s, 1H), 3.44 (td, *J* = 6.2, 1.4 Hz, 2H), 1.63 (p, *J* = 6.2 Hz, 1H), 1.41 (p, *J* = 6.4 Hz, 2H), 1.36 – 1.23 (m, 6H), 1.00 – 0.86 (m, 6H).

^{13}C NMR (101 MHz, $CDCl_3$) δ 168.65, 167.34, 148.95, 129.39, 122.08, 119.63, 113.85, 77.48, 77.16, 76.84, 43.21, 39.50, 31.14, 28.98, 24.40, 23.13, 14.21, 10.96.

HRMS (ES+): Calculated for $C_{15}H_{23}N_2O_4^+$ 295.1658, found 295.1658

Melting point: 35.0 °C

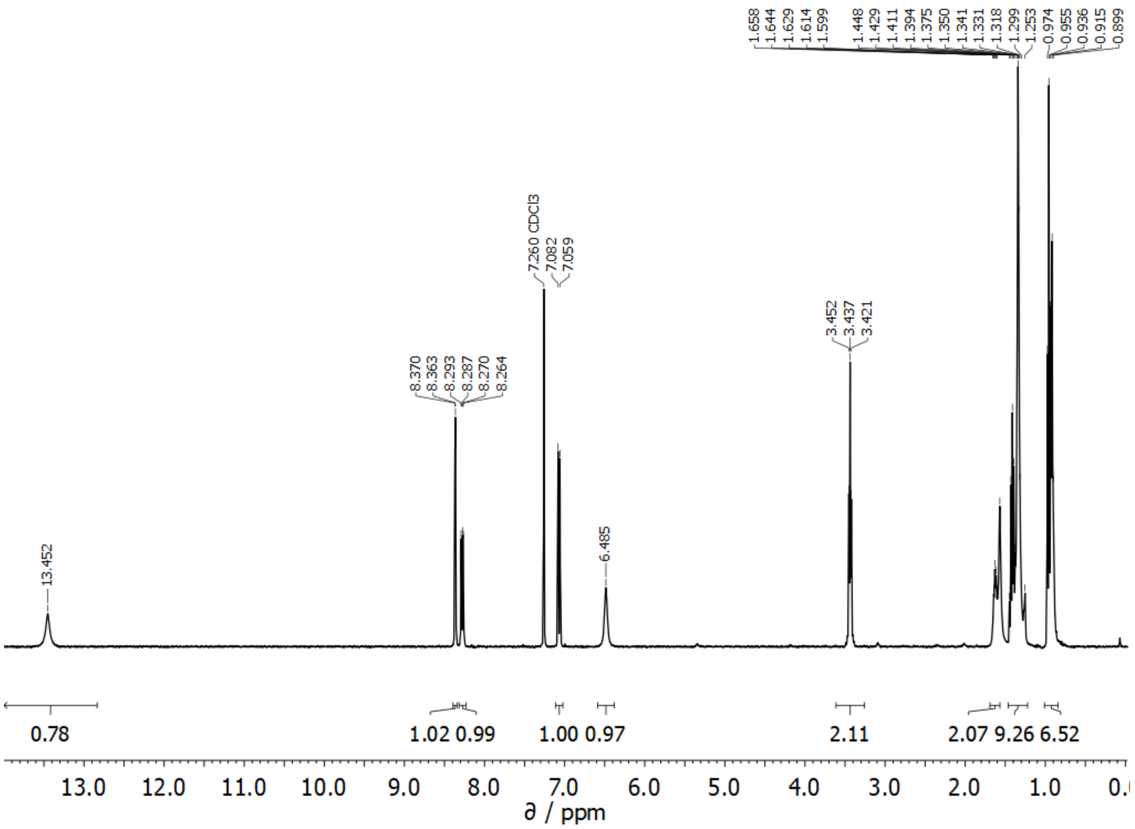

Figure S.9 – ^{1}H NMR spectrum of **5** in $CDCl_3$ (δ 0 – 14 ppm).

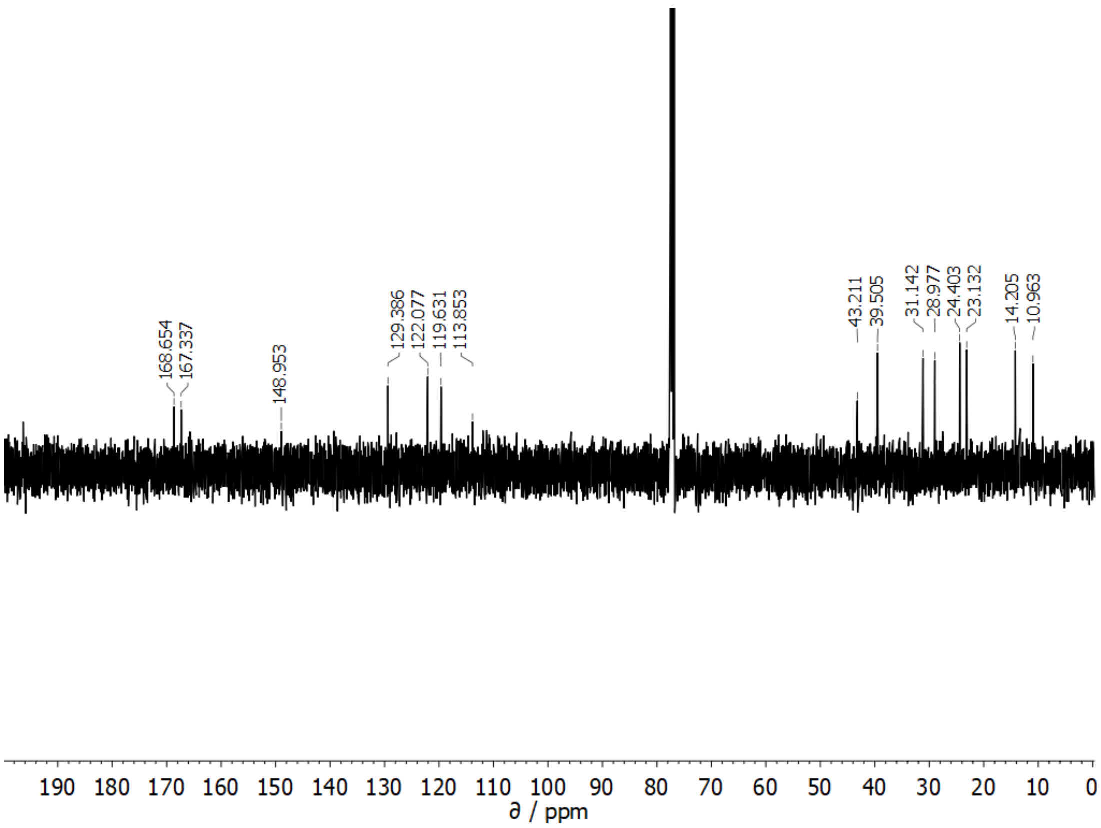

Figure S.10 – ^{13}C NMR spectrum of **5** in $CDCl_3$ (δ 0 – 200 ppm).

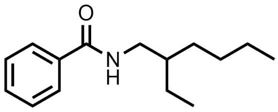

N-(2-ethylhexyl)benzamide, **6**. Benzoic acid (0.4025 g, 3.296 mmol), *N*-(3-dimethylaminopropyl)-*N'*-ethylcarbodiimide hydrochloride (0.6905 g, 3.602 mmol), and 4-dimethylamino pyridine (0.3385 g, 2.771 mmol) were combined in a flask. The flask was flushed with nitrogen, and 30 ml dry DCM was added, followed by 2-ethylhexylamine (0.46 ml, 2.8 mmol). The reaction mixture was stirred for 24 hours at room temperature under a slight positive pressure of nitrogen.

The resulting mixture was washed with sodium hydrogen carbonate (3x 50 ml), brine (2x 50 ml), 0.1 M HCl (2x 50 ml), and brine again (1x 50 ml) to yield the pure product as a faintly yellow oil (0.5730 g, 2.455 mmol, 89%).

FT-IR: ν_{max} (cm^{-1}) 3302 (N-H), 3063 (C-H), 2958 (C-H), 2926 (C-H), 2872 (C-H), 2858 (C-H), 1634 (C=O), 1603, 1578, 1540, 1490, 1459, 1436, 1378, 1293, 693, 670.

^{1}H NMR (400 MHz, Chloroform-*d*) δ 7.75 (d, *J* = 7.4 Hz, 2H), 7.53 – 7.46 (m, 1H), 7.43 (t, *J* = 7.4 Hz, 2H), 6.06 (s, 1H), 3.41 (td, *J* = 6.1, 3.2 Hz, 2H), 1.57 (p, *J* = 4.1 Hz, 1H), 1.39 (p, *J* = 7.3 Hz, 2H), 1.35 – 1.25 (m, 6H), 0.92 (dt, *J* = 13.4, 7.0 Hz, 6H).

^{13}C NMR (101 MHz, $CDCl_3$) δ 167.56, 135.00, 131.27, 128.55, 126.76, 77.31, 76.99, 76.68, 42.88, 39.51, 31.13, 28.91, 24.38, 23.01, 14.06, 10.94.

HRMS (ES+): Calculated for $C_{15}H_{24}NO^{+}$ 234.1858, found 234.1856

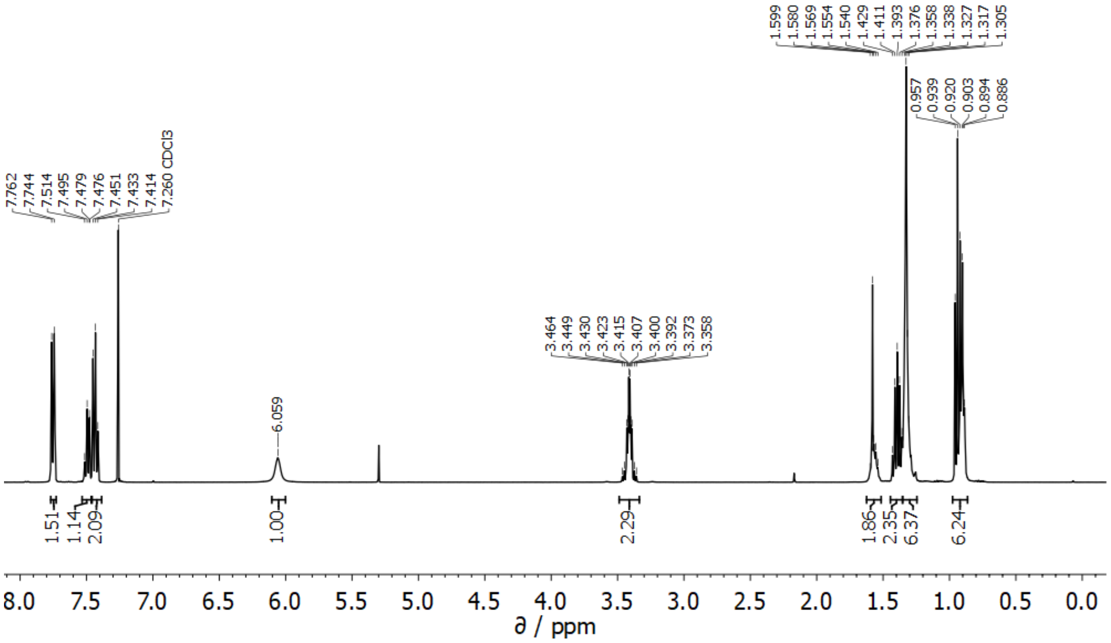

Figure S.11 – ^{1}H NMR spectrum of **6** in $CDCl_3$ (δ 0 – 10 ppm).

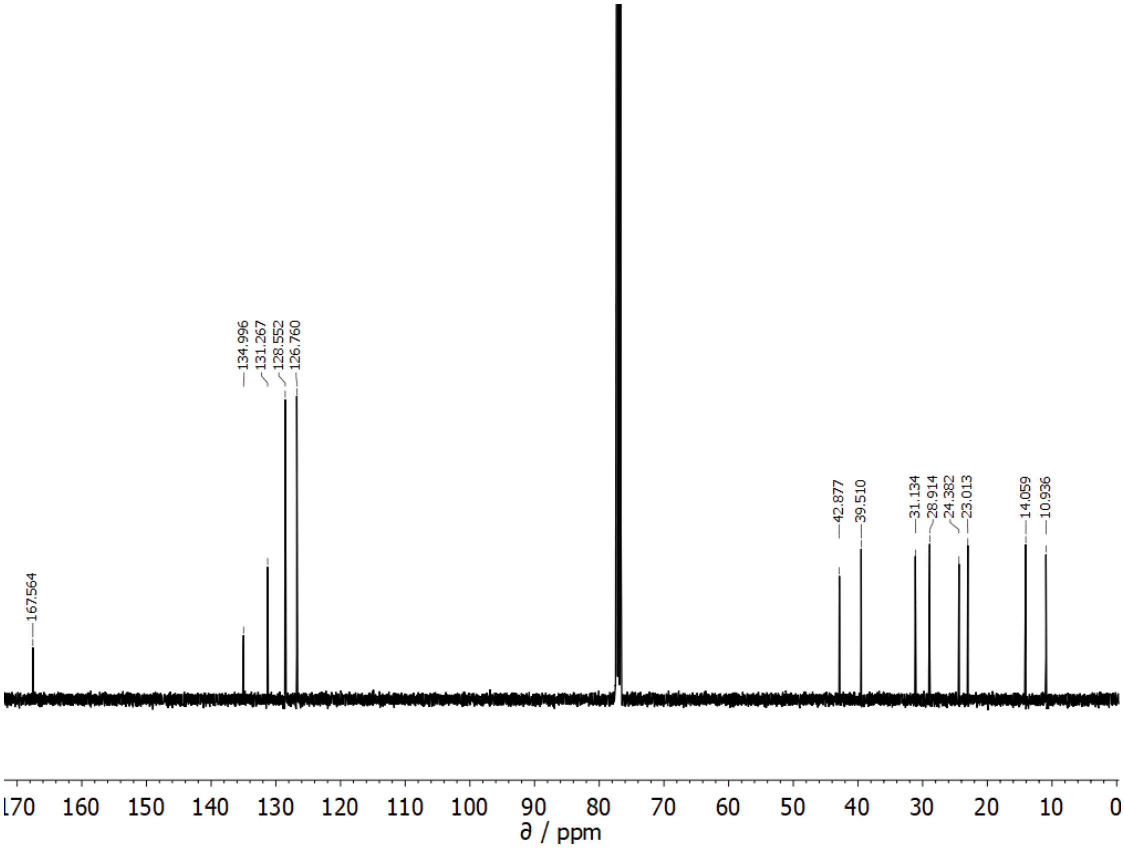

Figure S.12 – ^{13}C NMR spectrum of **6** in $CDCl_3$ (δ 0 – 200 ppm).

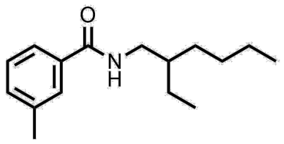

N-(2-ethylhexyl)-3-methylbenzamide, **7**. 3-methylbenzoic acid (0.495 g, 3.64 mmol), EDC hydrochloride (0.799 g, 4.17 mmol) and DMAP (0.434 g, 3.86 mmol) were placed under a N_2 atmosphere and dissolved in dry DCM (20 mL). 2-ethylhexylamine (0.65 mL, 4.0 mmol) was added and the mixture stirred at room temperature for 24 hours. The reaction mixture was diluted in DCM (30 mL), washed with saturated $NaHCO_3$ (2 x 50 mL), HCl (1 M, 2 x 50 mL) and water (50 mL). The organic layer was dried with $MgSO_4$ and the solvent removed in vacuo to yield the desired product **4** as a clear oil (0.559 g, 2.26 mmol, 62%).

ν_{max} (film) cm^{-1} : 3308 (N-H), 2958 (C-H), 2926 (C-H), 2872 (C-H), 2859 (C-H), 1636 (C=O) 1607 (C=C), 1586 (C=C), 1542 (C=C), 1486, 1461, 1302, 743, 693

^1H NMR (400 MHz, $CDCl_3$) δ_H ppm: 7.58 (s, 1H), 7.51 (d, J = 6.0 Hz, 1H), 7.30 (d, J = 5.0 Hz, 2H), 6.06 (s, 1H) 3.40 (td, J = 7.3, 6.5, 3.2 Hz, 2H), 2.40 (s, 3H), 1.56 (p, J = 5.6 Hz, 1H), 1.40 (q, J = 7.3 Hz, 2H), 1.32 (s, 7H), 0.97-0.86 (m, 6H).

$^{13}C\{^1H\}$ NMR (101 MHz, $CDCl_3$) δ_C ppm: 167.91, 138.56, 135.14, 132.13, 128.55, 127.76, 123.81, 43.02, 39.68, 31.28, 29.07, 24.53, 23.17, 21.52, 14.22, 11.08.

HRMS: calc for $C_{16}H_{26}NO^+$ $[M+H]^+$: 248.2014, found: 248.2009

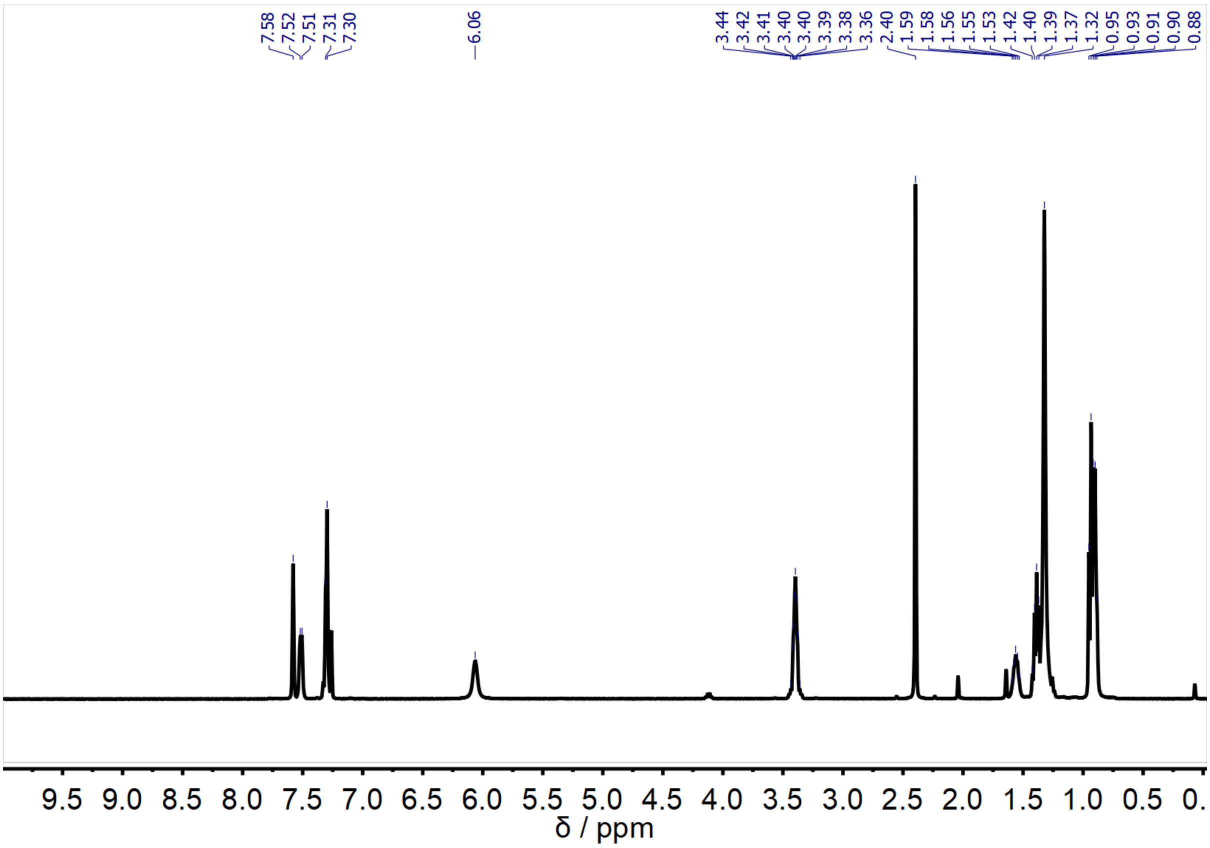

Figure S.13 – ^1H NMR spectrum of **7** in $CDCl_3$ (δ 0 – 10 ppm).

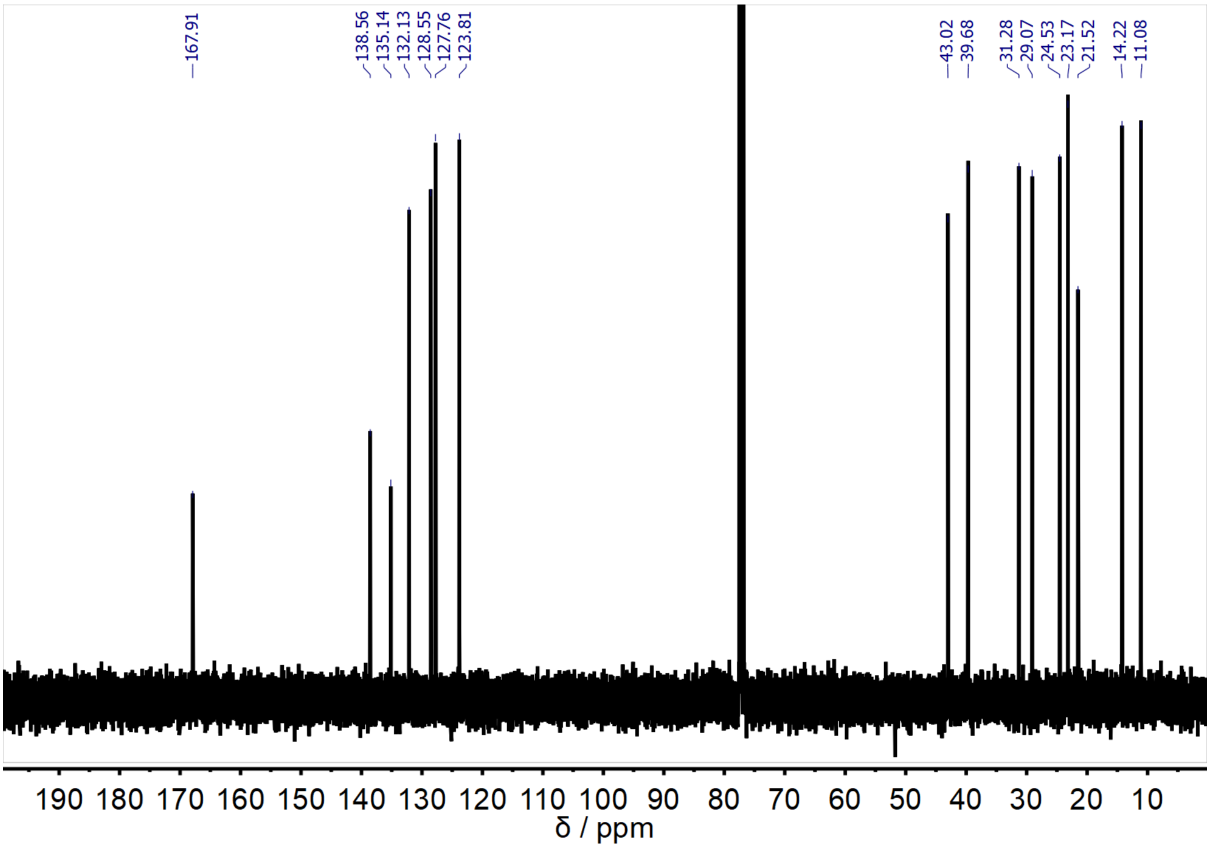

Figure S.14 – ^{13}C NMR spectrum of **7** in $CDCl_3$ (δ 0 – 200 ppm).

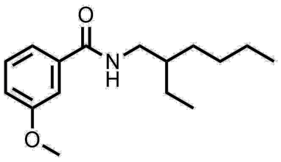

N-(2-ethylhexyl)-3-methoxybenzamide, **8**. 3-methoxybenzoic acid (0.537 g, 3.53 mmol), EDC hydrochloride (0.799 g, 4.17 mmol) and DMAP (0.434 g, 3.65 mmol) were placed under a N_2 atmosphere and dissolved in dry DCM (20 mL). 2-ethylhexylamine (0.65 mL, 4.0 mmol) was added and the mixture stirred at room temperature for 18 hours. The reaction mixture was diluted in DCM (30mL), washed with saturated $NaHCO_3$ (2 x 50 mL), HCl (1 M, 2 x 50 mL) and water (50 mL). The organic layer was dried with $MgSO_4$ and the solvent removed in vacuo to yield the desired product **5** as a clear oil (0.820 g, 3.12 mmol, 88%).

ν_{max} (film) cm^{-1} : 3308 (N-H), 2958 (C-H), 2927 (C-H) 2872 (C-H), 2858 (C-H), 1635 (C=O), 1602 (C=C), 1583 (C=C), 1542 (C=C), 1486, 1464, 1434, 1302, 1287, 1245, 1048, 753, 690

^{1}H NMR (400 MHz, $CDCl_3$) δ_H ppm: δ 7.37 (s, 1H), 7.33 (dd, J = 7.9, 2.0 Hz, 1H), 7.28 (s, 1H), 7.04 (d, J = 8.0 Hz, 1H), 6.11 (s, 1H), 3.87 (d, J = 2.3 Hz, 3H), 3.41 (tt, J = 5.8, 2.4 Hz, 2H), 1.57 (q, J = 5.9 Hz, 1H), 1.45 – 1.37 (m, 2H), 1.34 (s, 7H), 0.99 – 0.85 (m, 6H).

^{13}C{^{1}H} NMR (101 MHz, $CDCl_3$) δ_C ppm: 167.57, 159.97, 136.64, 129.66, 118.58, 117.57, 112.48, 55.58, 43.07, 39.66, 31.28, 29.07, 24.53, 23.16, 14.21, 11.09.

HRMS: calc for $C_{16}H_{26}NO_2^+$ $[M+H]^+$: 264.1964, found: 294.1959

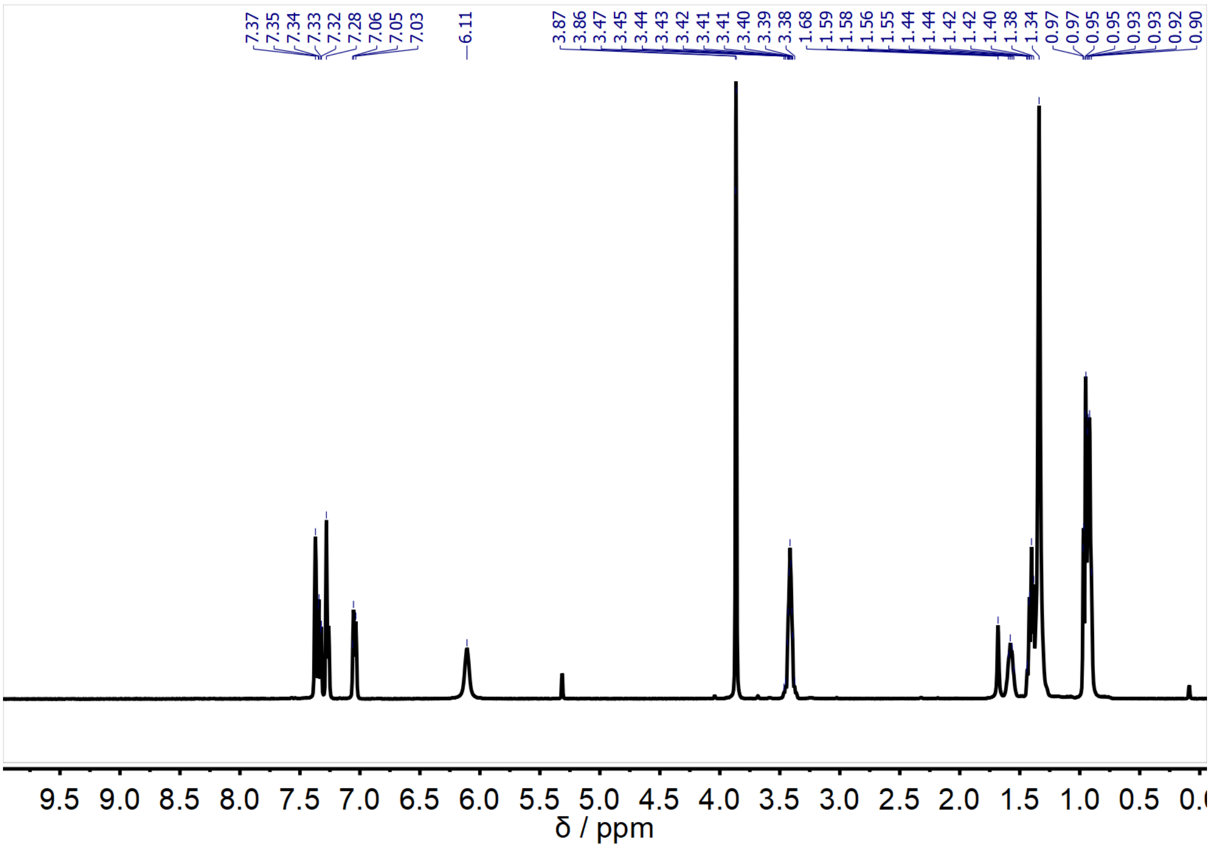

Figure S.15 – ^{1}H NMR spectrum of **8** in $CDCl_3$ (δ 0 – 10 ppm).

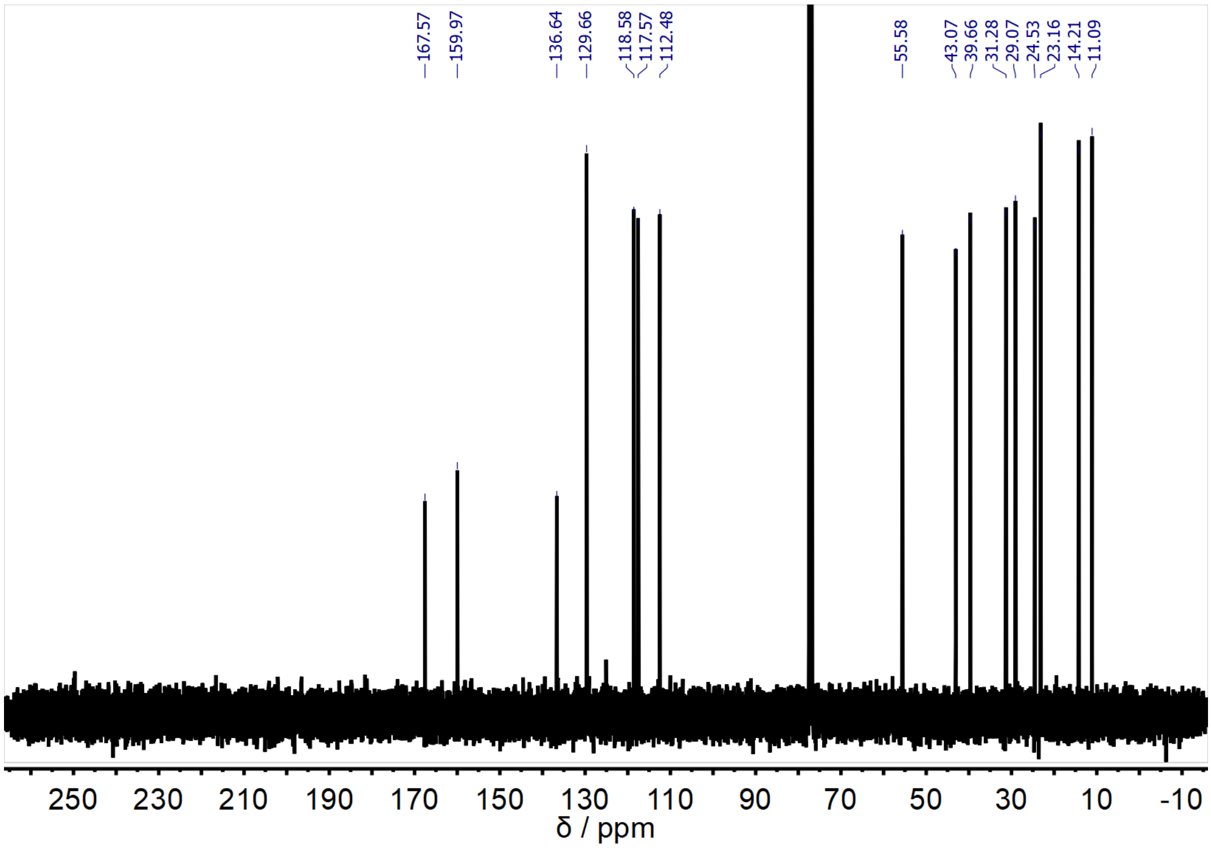

Figure S.16 – ^{13}C NMR spectrum of **8** in $CDCl_3$ (δ 0 – 200 ppm).

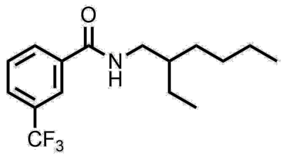

N-(2-ethylhexyl)-3-(trifluoromethyl)benzamide, **9**. 3-(trifluoromethyl)benzoic acid (0.613 g, 3.23 mmol), EDC hydrochloride (0.648 g, 3.38 mmol) and DMAP (0.0.372 g, 3.32 mmol) were placed under a N_2 atmosphere and dissolved in dry THF (30 mL). 2-ethylhexylamine (0.58 mL, 3.5 mmol) was added and the mixture stirred at room temperature for 28 hours. The reaction mixture was diluted in DCM (50 mL), and washed with brine (3 x30 mL) The organic layer was dried with $MgSO_4$ and the solvent removed in vacuo. The crude was purified by flash column chromatography (PE/EtOAc on SiO_2) to yield the desired product as a clear oil (0.771 g, 2.56 mmol, 79%).

ν_{max} (film) cm^{-1} : 3304 (N-H), 2960 (C-H), 2929 (C-H), 2874 (C-H), 2861 (C-H), 1640 (C=O), 1551 (C=C), 1332, 1302, 1278, 1170, 1130, 1075, 698

^1H NMR (400 MHz, $CDCl_3$) δ_H ppm: 8.01 (s, 1H), 7.93 (d, J = 7.8 Hz, 1H), 7.75 (d, J = 7.8 Hz, 1H), 7.57 (t, J = 7.8 Hz, 1H), 6.12 (s, 1H), 3.42 (td, J = 6.2, 2.3 Hz, 2H), 1.58 (d, J = 6.7 Hz, 1H), 1.39 (p, J = 7.4 Hz, 3H), 1.32 (d, J = 4.1 Hz, 5H), 0.97 – 0.88 (m, 6H).

$^{13}C\{^1H\}$ NMR (101 MHz, $CDCl_3$) δ_C ppm:.31, 135.93, 131.46, 131.14, 130.21, 129.36, 128.09, 128.05, 125.21, 124.02, 123.98, 43.33, 39.66, 31.26, 29.06, 24.52, 23.16, 14.20, 11.05.

HRMS: calc for $C_{16}H_{23}F_3NO^+$ $[M+H]^+$: 302.1732, found: 302.1731

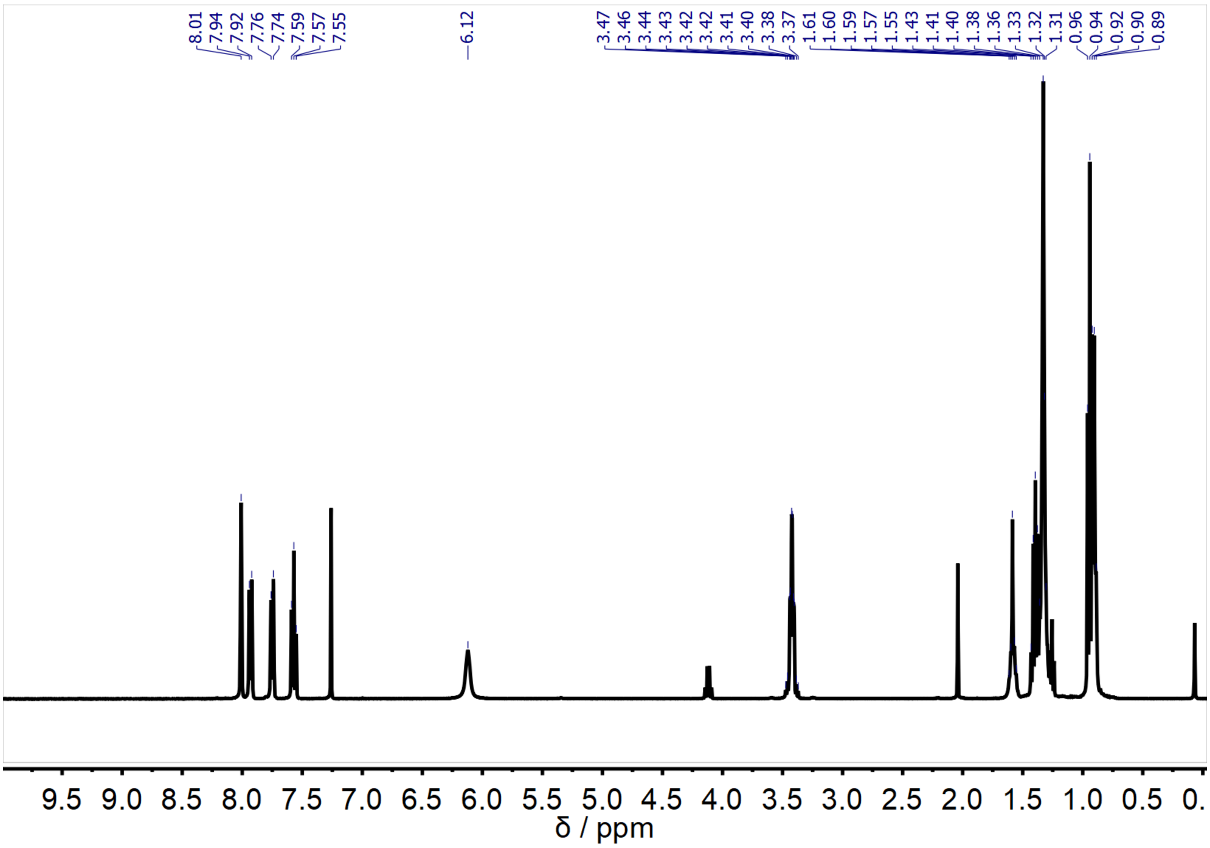

Figure S.17 – ^1H NMR spectrum of **9** in $CDCl_3$ (δ 0 – 10 ppm).

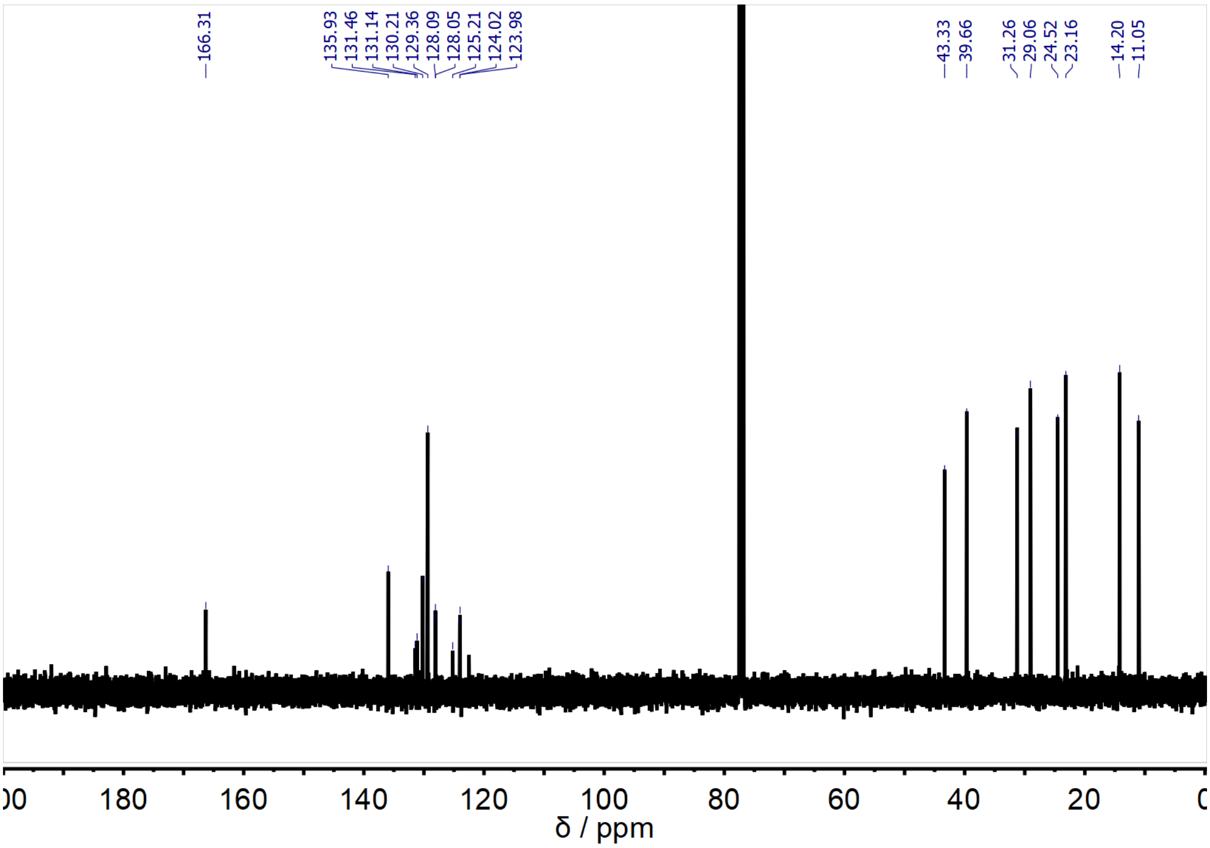

Figure S.18 – ^{13}C NMR spectrum of **9** in $CDCl_3$ (δ 0 – 200 ppm).

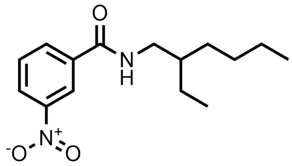

N-(2-ethylhexyl)-3-nitrobenzamide, **10**. 3-nitrobenzoic acid (0.4528 g, 2.709 mmol), *N*-(3-dimethylaminopropyl)-*N*'-ethylcarbodiimide hydrochloride (0.6542 g, 3.413 mmol), and 4-dimethylamino pyridine (0.3400 g, 2.783 mmol) were combined in a flask. The flask was flushed with nitrogen, and 30 ml dry THF was added, followed by 2-ethylhexylamine (0.46 ml, 2.8 mmol). The reaction mixture was stirred for 24 hours at room temperature under a slight positive pressure of nitrogen.

The resulting mixture was filtered under gravity. DCM (30 ml) was added, and the combined organic layer was washed with brine (3x50 ml), dried with MgSO4, and filtered under gravity again. The crude product was purified by flash chromatography (EtOAc:DCM on SiO_2) to yield the pure product as a yellow gel (0.5227 g, 1.878 mmol, 69%)

FT-IR: ν_{max} (cm^{-1}) 3313 (N-H), 3083 (C-H), 2928 (C-H), 2872 (C-H), 2859 (C-H), 1639 (C=O), 1618, 1528, 1463, 1437, 1348, 1161, 1080, 815, 717.

^{1}H NMR (400 MHz, Chloroform-*d*) δ 8.56 (s, 1H), 8.35 (dd, *J* = 8.3, 2.1 Hz, 1H), 8.14 (d, *J* = 7.7 Hz, 1H), 7.65 (t, *J* = 7.9 Hz, 1H), 6.19 (s, 1H), 3.44 (td, *J* = 6.2, 2.4 Hz, 2H), 1.61 (d, *J* = 5.8 Hz, 1H), 1.41 (p, *J* = 7.3 Hz, 2H), 1.37 – 1.29 (m, 5H), 1.26 (t, *J* = 7.1 Hz, 1H), 1.03 – 0.85 (m, 6H).

^{13}C NMR (101 MHz, $CDCl_3$) δ 165.13, 148.18, 136.51, 133.13, 129.86, 125.92, 121.57, 77.32, 77.00, 76.68, 43.32, 39.47, 31.08, 28.88, 24.33, 23.00, 14.06, 10.88.

HRMS (ES+): Calculated for $C_{15}H_{23}N_2O_3^+$ 279.1709, found 279.1696

Melting point: 94.9 °C

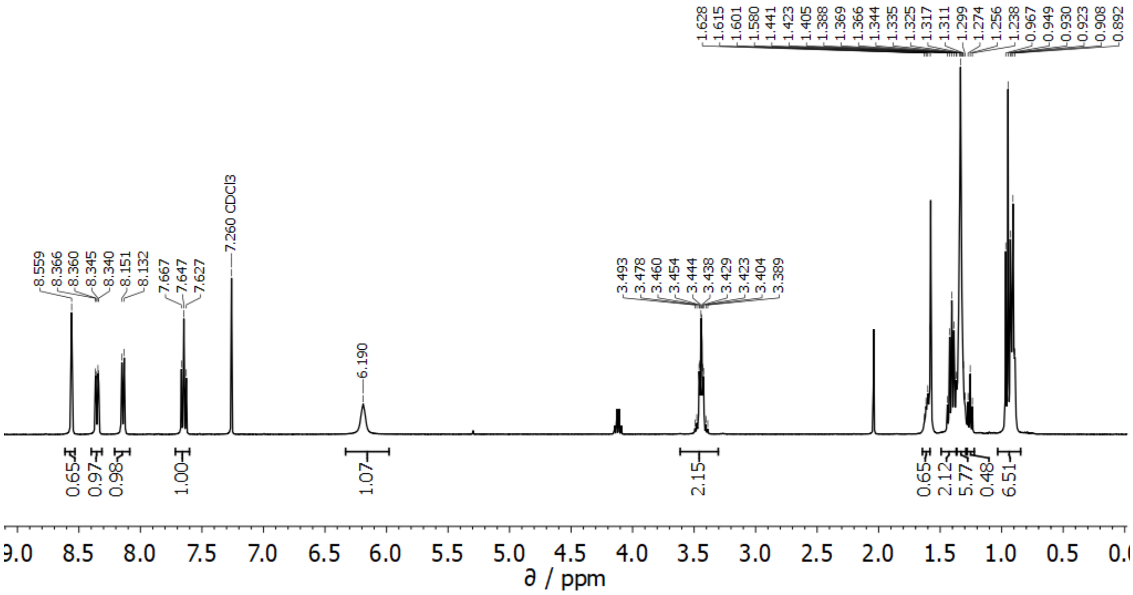

Figure S.19 – ^{1}H NMR spectrum of **10** in $CDCl_3$ (δ 0 – 10 ppm).

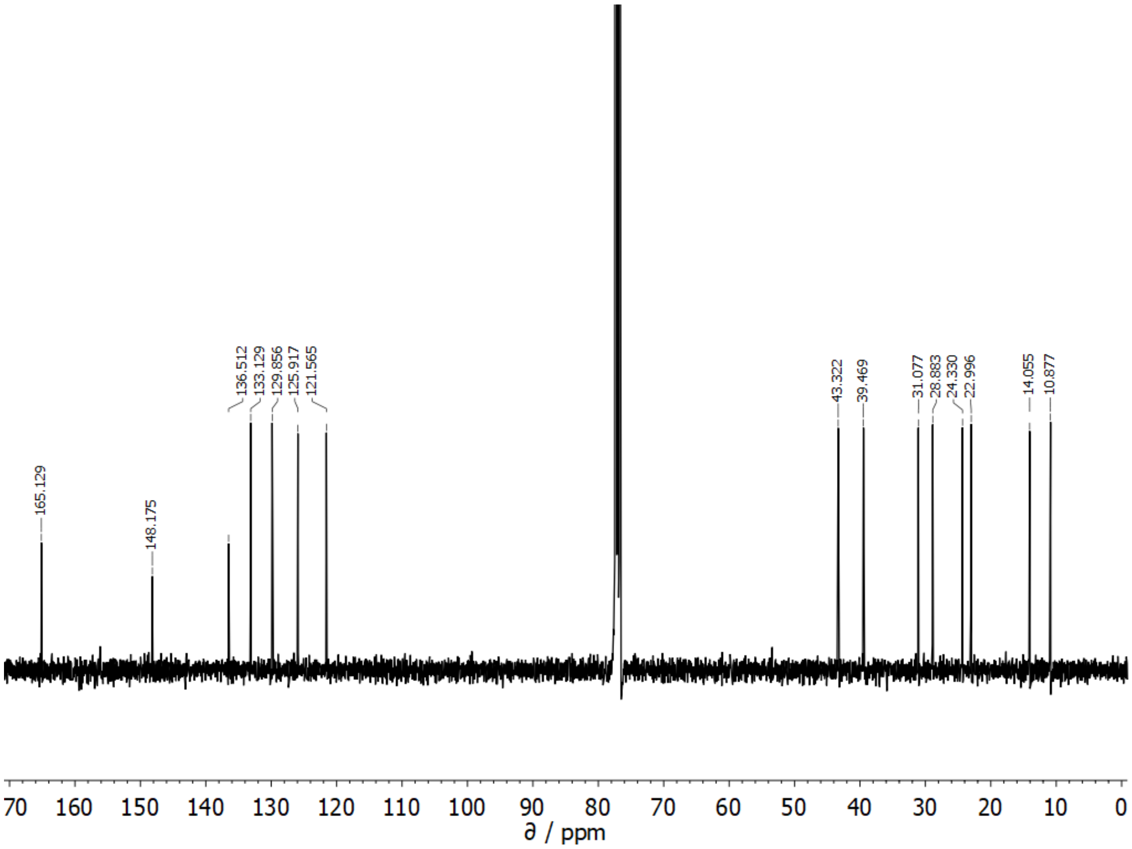

Figure S.20 – ^{13}C NMR spectrum of **10** in $CDCl_3$ (δ 0 – 200 ppm).

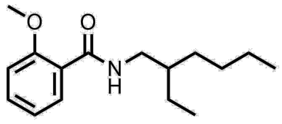

N-(2-ethylhexyl)-2-methoxybenzamide, **11**. 2-methoxybenzoic acid (0.483 g, 3.17 mmol), EDC hydrochloride (0.666 g, 3.47 mmol) and DMAP (0.387 g, 3.45 mmol) were placed under a N_2 atmosphere and dissolved in dry DCM (20 mL). 2-ethylhexylamine (0.60 mL, 3.7 mmol) was added and the mixture stirred at room temperature for 24 hours. The reaction mixture was diluted in DCM (30mL), washed with saturated $NaHCO_3$ (2 x 50 mL), HCl (1 M, 2 x 50 mL) and water (50 mL). The organic layer was dried with $MgSO_4$ and the solvent removed in vacuo to yield the desired product as a clear oil (0.698 g, 2.65 mmol, 84%).

ν_{max} (film) cm^{-1} : 3410 (N-H), 2957 (C-H), 2926 (C-H), 2872 (C-H), 2858 (C-H), 1651 (C=O), 1600 (C=C), 1535 (C=C), 1483, 1464, 1296, 1237, 1021, 755

^1H NMR (400 MHz, $CDCl_3$) δ_H ppm: 8.22 (d, J = 7.7 Hz, 1H), 7.87 (s, 1H), 7.43 (t, J = 7.9 Hz, 1H), 7.08 (t, J = 7.6 Hz, 1H), 6.97 (d, J = 8.3 Hz, 1H), 3.96 (d, J = 1.8 Hz, 3H), 3.43 (p, J = 6.6, 5.6 Hz, 2H), 1.57 (p, J = 5.8 Hz, 1H), 1.40 (q, J = 7.3 Hz, 2H), 1.33 (s, 7H), 1.00 – 0.88 (m, 7H).

$^{13}C\{^1H\}$ NMR (101 MHz, $CDCl_3$) δ_C ppm: 165.35, 157.51, 132.60, 132.44, 122.04, 121.49, 111.41, 56.09, 42.56, 39.42, 31.53, 29.10, 24.79, 23.22, 14.23, 11.19.

HRMS: calc for $C_{16}H_{26}NO_2^+$ $[M+H]^+$: 264.1964, found: 264.1958

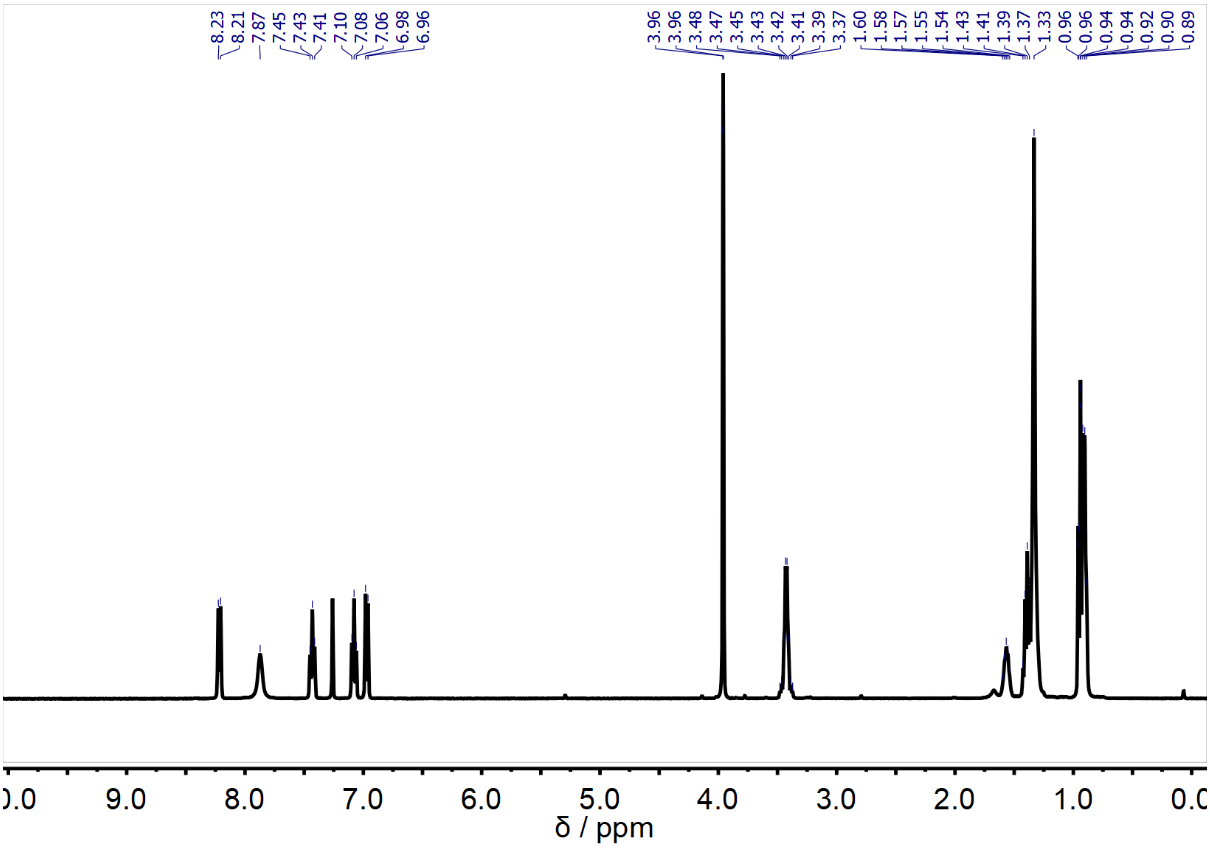

Figure S.21 – ^1H NMR spectrum of **11** in $CDCl_3$ (δ 0 – 10 ppm).

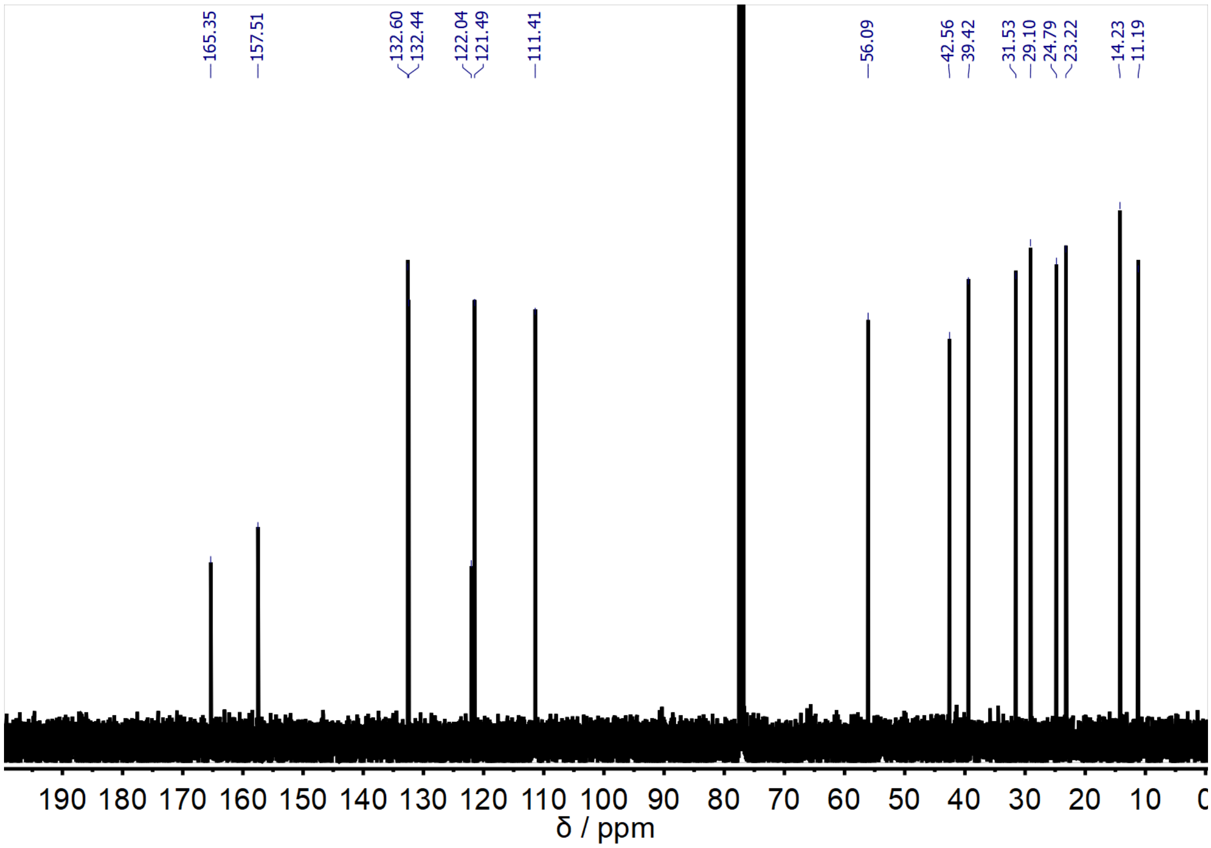

Figure S.22 – ^{13}C NMR spectrum of **11** in $CDCl_3$ (δ 0 – 200 ppm).

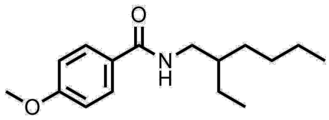

N-(2-ethylhexyl)-4-methoxybenzamide, **12**. 4-methoxybenzoic acid (0.494 g, 3.25 mmol), EDC hydrochloride (0.671 g, 3.50 mmol) and DMAP (0.378 g, 3.37 mmol) were placed under a N_2 atmosphere and dissolved in dry DCM (30 mL). 2-ethylhexylamine (0.60 mL, 3.7 mmol) was added and the mixture stirred at room temperature for 24 hours. The reaction mixture was diluted in DCM (20mL), washed with saturated $NaHCO_3$ (2 x 50 mL), HCl (1 M, 2 x 50 mL) and water (50 mL). The organic layer was dried with $MgSO_4$ and the solvent removed in vacuo to yield the desired product **5** as a white solid (0.8072 g, 3.07 mmol, 94%).

m.p. 50–55 °C

ν_{max} (film) cm^{-1} : 3314 (N-H), 2957 (C-H), 2927 (C-H), 2858 (C-H), 1631 (C=O), 1607 (C=C), 1574 (C=C), 1546 (C=C), 1504 (C=C), 1462, 1441, 1296, 1253, 1178, 1033, 844, 768

^{1}H NMR (400 MHz, $CDCl_3$) δ_H ppm: 7.72 (d, J = 7.9 Hz, 2H), 6.92 (d, J = 7.8 Hz, 2H), 6.00 (s, 1H), 3.84 (s, 3H), 3.38 (tt, J = 5.7, 2.2 Hz, 2H), 1.54 (q, J = 6.0 Hz, 1H), 1.45 – 1.35 (m, 2H), 1.32 (s, 7H), 0.98 – 0.82 (m, 6H).

$^{13}C\{^1H\}$ NMR (101 MHz, $CDCl_3$) δ_C ppm: 167.21, 162.15, 128.69, 127.42, 113.87, 55.54, 43.00, 39.70, 31.30, 29.08, 24.55, 23.18, 14.22, 11.10.

HRMS: calc for $C_{16}H_{26}NO_2^+$ $[M+H]^+$: 264.1964, found: 264.1958

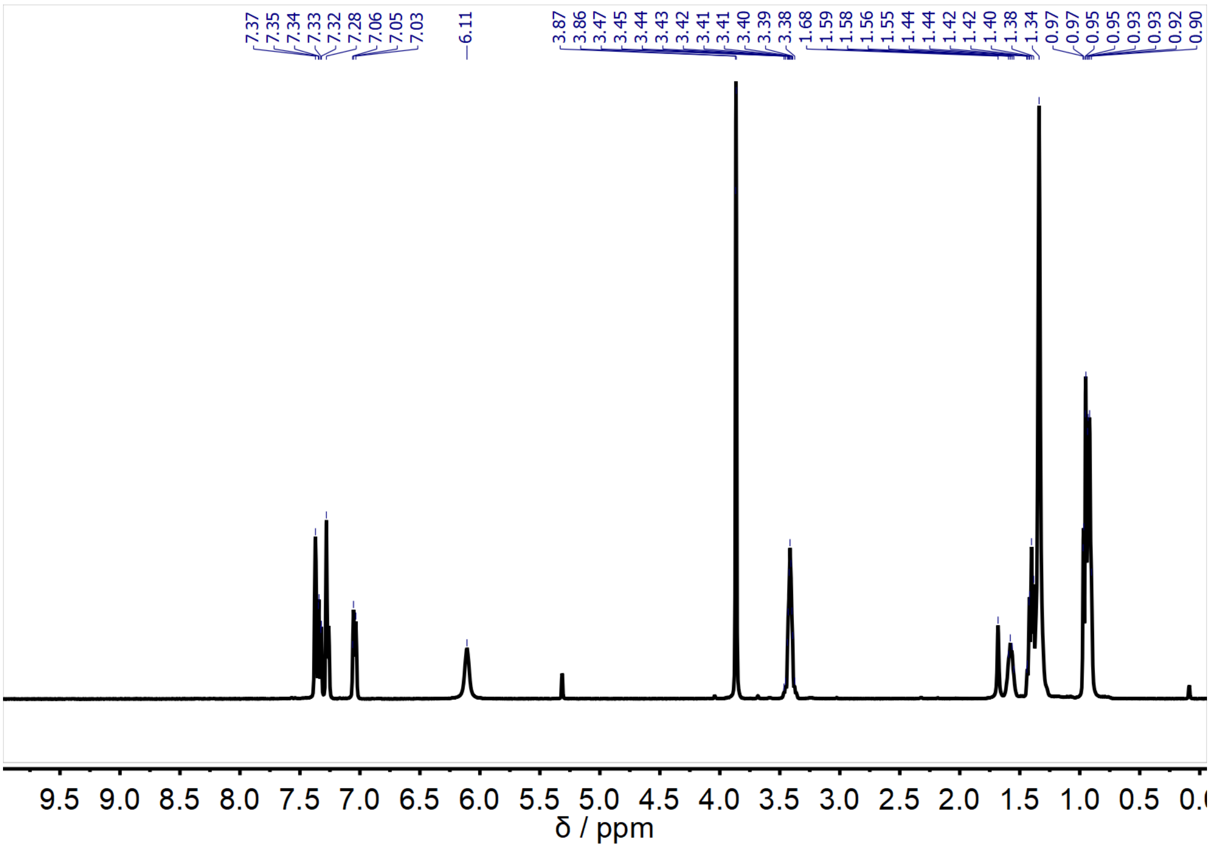

Figure S.23 – ^{1}H NMR spectrum of **12** in $CDCl_3$ (δ 0 – 10 ppm).

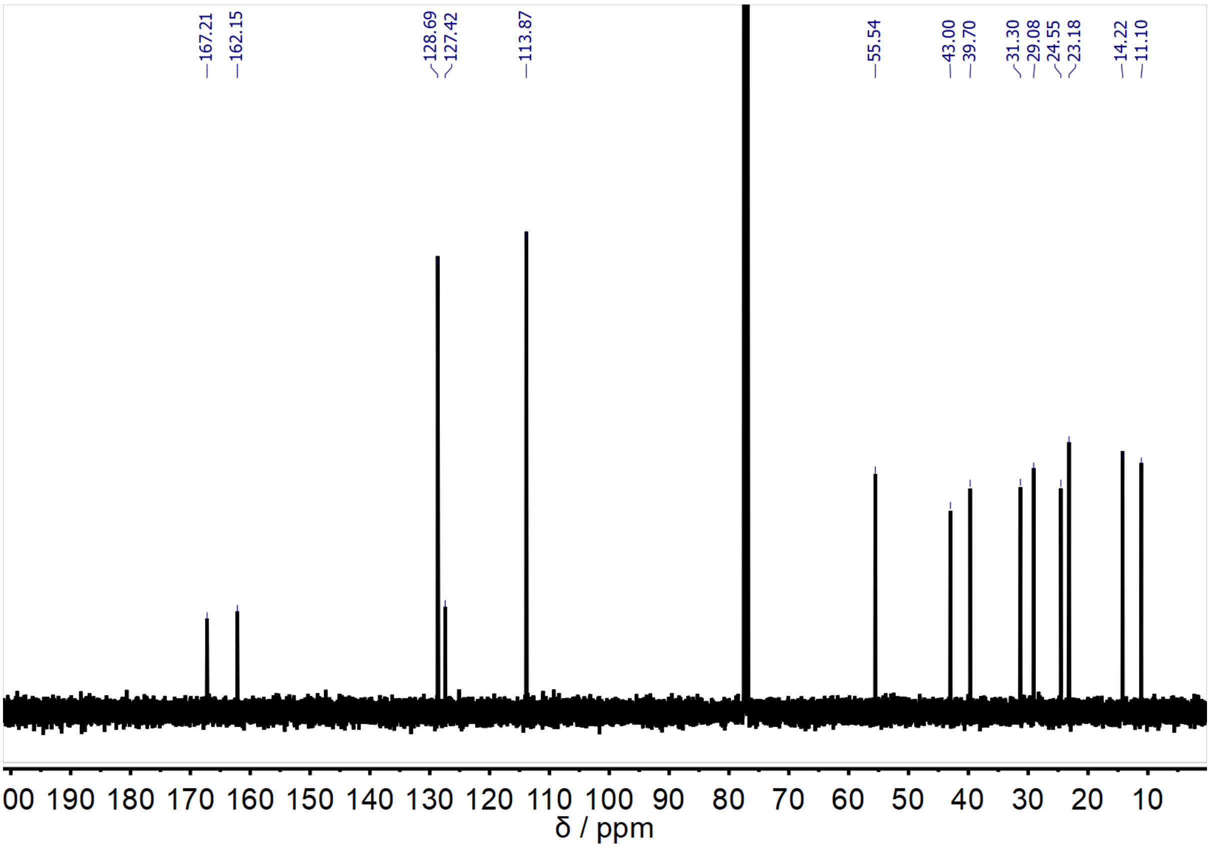

Figure S.24 – ^{13}C NMR spectrum of **12** in $CDCl_3$ (δ 0 – 200 ppm).

UV/vis absorption spectroscopy titrations

Titrations were carried out on an Agilent Cary 60 UV-Vis spectrophotometer, using standard titration protocols. First, dilution experiments were carried out by adding aliquots of a concentrated host solution in *n*-octane (3 mM) to a cuvette containing *n*-octane and recording the UV/vis absorption spectrum. The data obey a Lambert-Beer law, indicating no self-association in this concentration range (see below). A 5 mL sample of the host was prepared at a known concentration (typically between 0.050-0.110 mM) in *n*-octane. The UV/Vis spectrum of the free host (2 mL) was recorded. The guest was dissolved in 2 mL of the host solution. Aliquots of the guest solution were successively added to the cuvette, and the UV/Vis absorption spectrum was recorded after each addition. The UV/Vis absorption spectra were analysed using a purpose-built Python script to fit the changes in the absorption at fixed wavelengths to a 1:1 binding isotherm by optimizing the association constant and absorption of the free host, guest and the complex using a purpose-written Python fitting script.

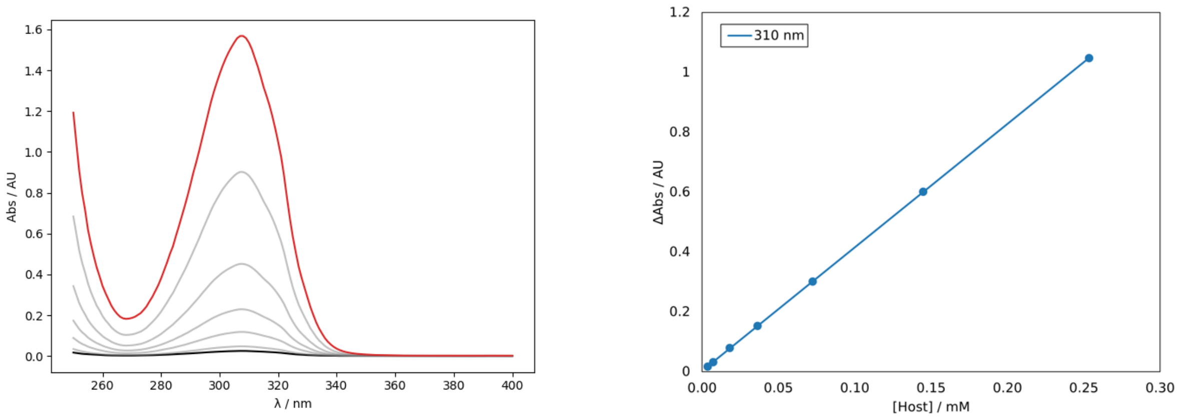

Figure S.25 - UV/Vis absorption spectra of compound **1** at in *n*-octane at 298K, and a Lambert-Beer plot of the absorption at 310 nm.

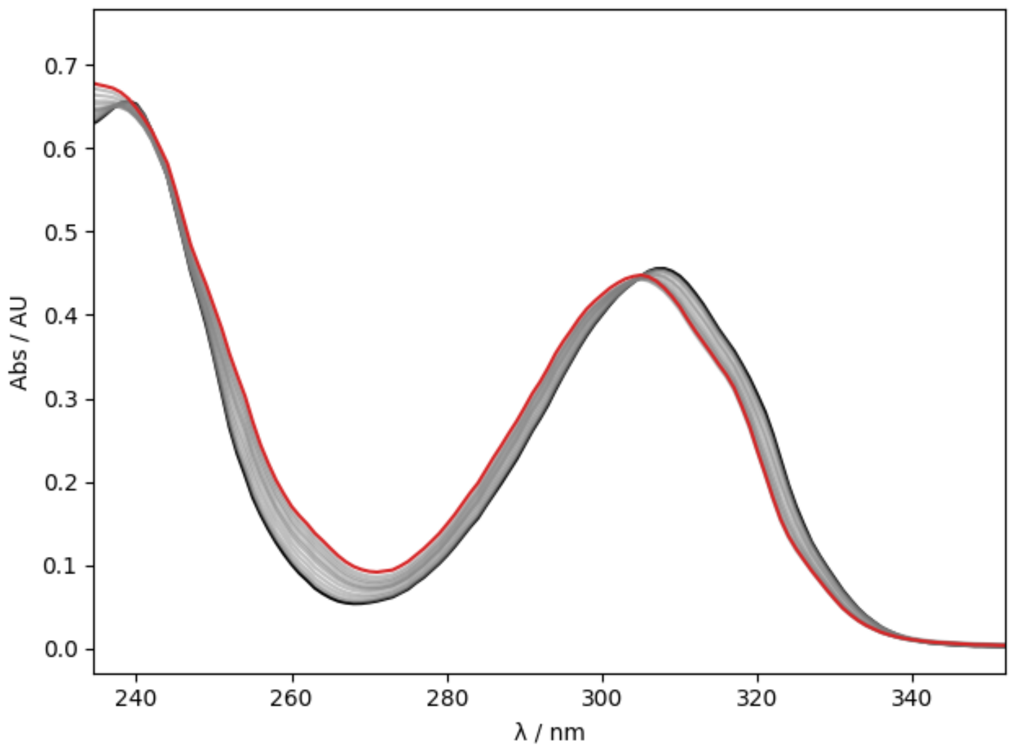

Figure S.26 - UV/Vis absorption spectra for the titration of **Bu_3PO** into **1** (in *n*-octane, at 298K). The UV/Vis spectrum of the host **1** and the final point of the titration are reported in black and in red, respectively.

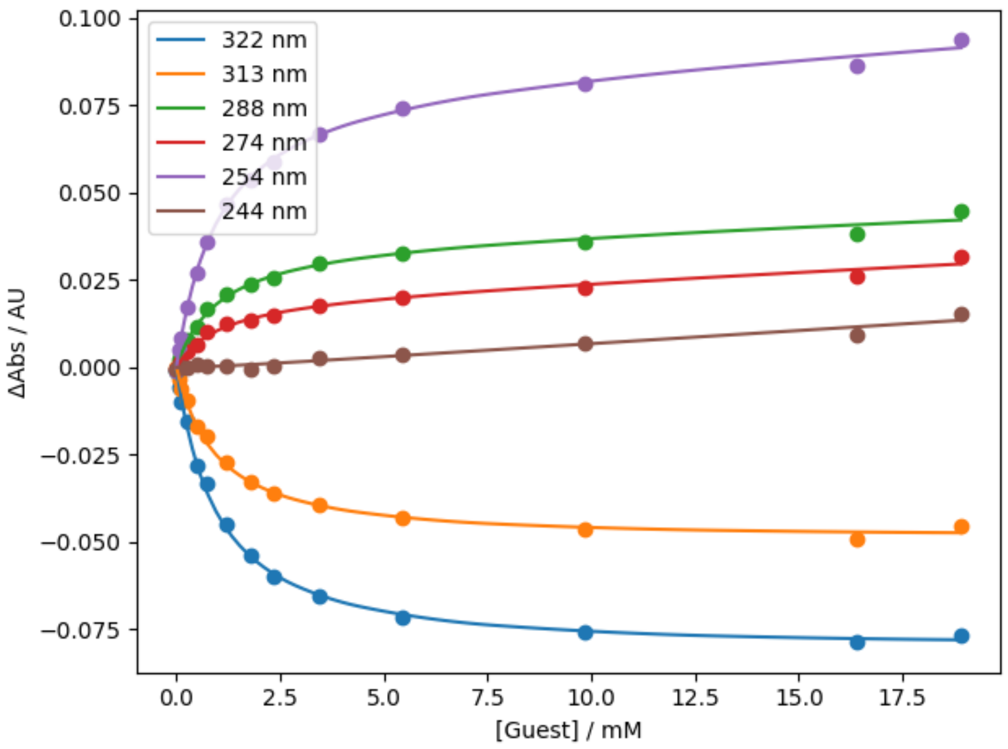

Figure S.27 -*1* Changes in UV/Vis absorbance from the titrations of **Bu_3PO** into **1** in *n*-octane (at 298 K) at selected wavelengths (points), and the fits to a 1:1 binding isotherm with guest absorption (solid lines).

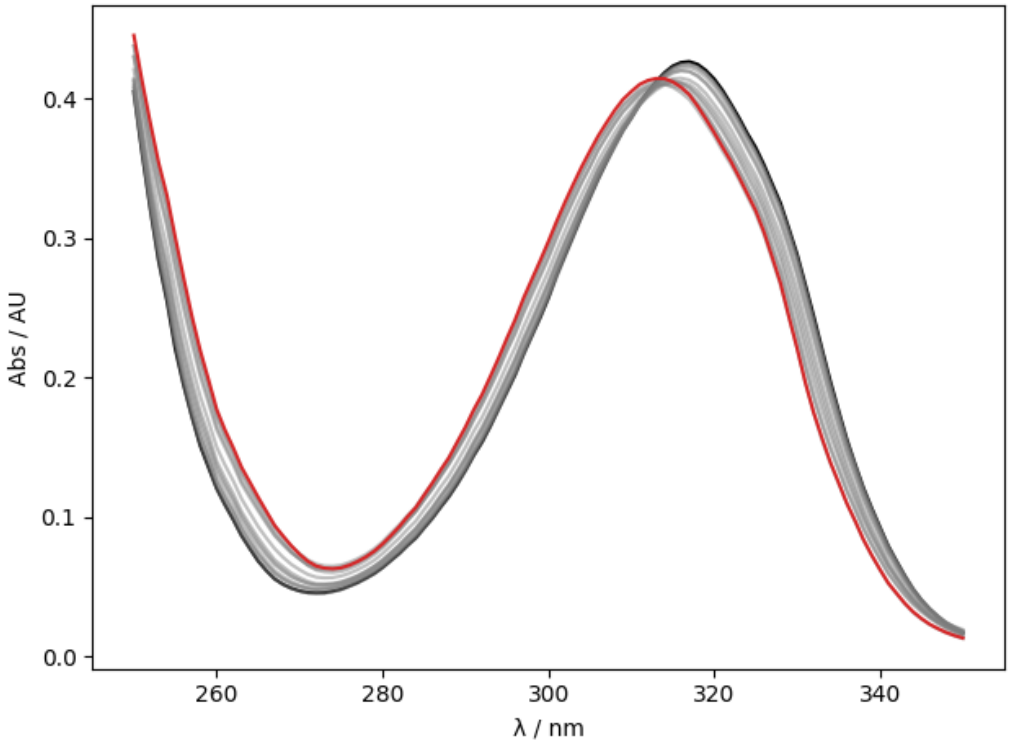

Figure S.28 - UV/Vis absorption spectra for the titration of Bu_3PO into **2** (in *n*-octane, at 298K). The UV/Vis spectrum of the host **2** and the final point of the titration are reported in black and in red, respectively.

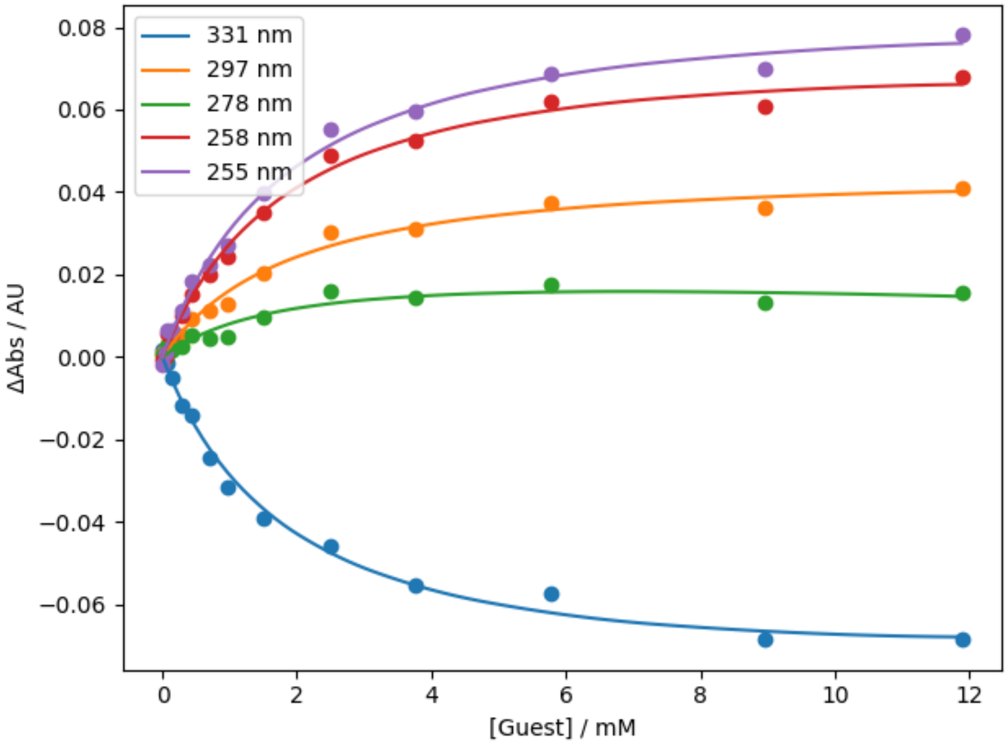

Figure S.29 -*2* Changes in UV/Vis absorbance from the titrations of Bu_3PO into **2** in *n*-octane (at 298 K) at selected wavelengths (points), and the fits to a 1:1 binding isotherm with guest absorption (solid lines).

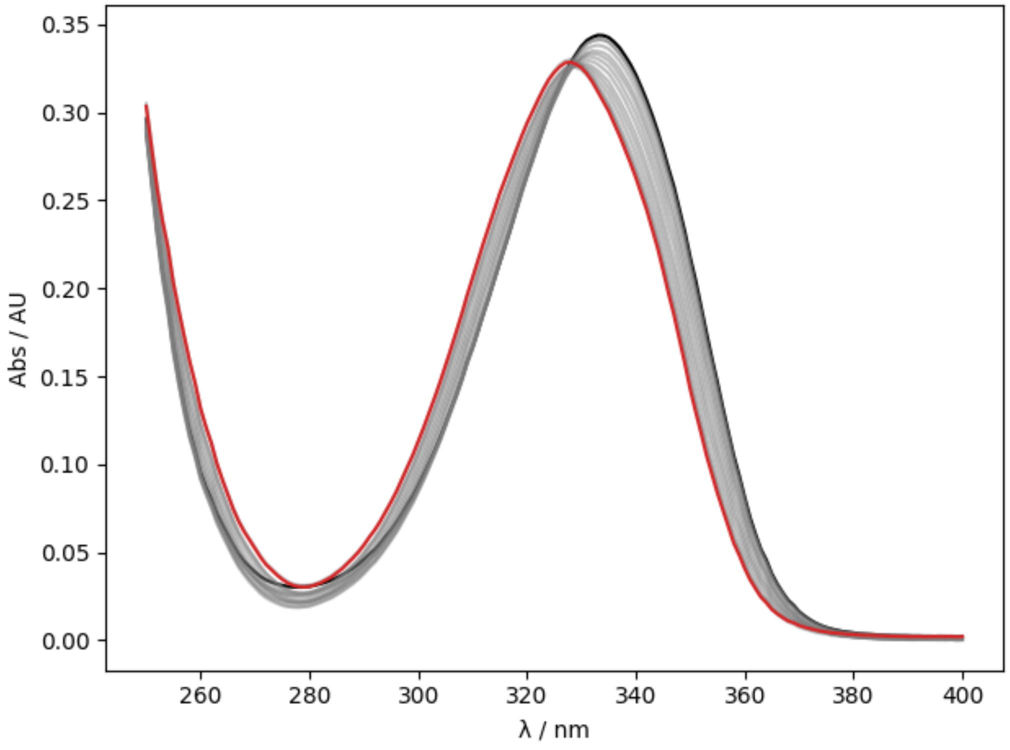

Figure S.30 - UV/Vis absorption spectra for the titration of **Bu_3PO** into **3** (in *n*-octane, at 298K). The UV/Vis spectrum of the host **3** and the final point of the titration are reported in black and in red, respectively.

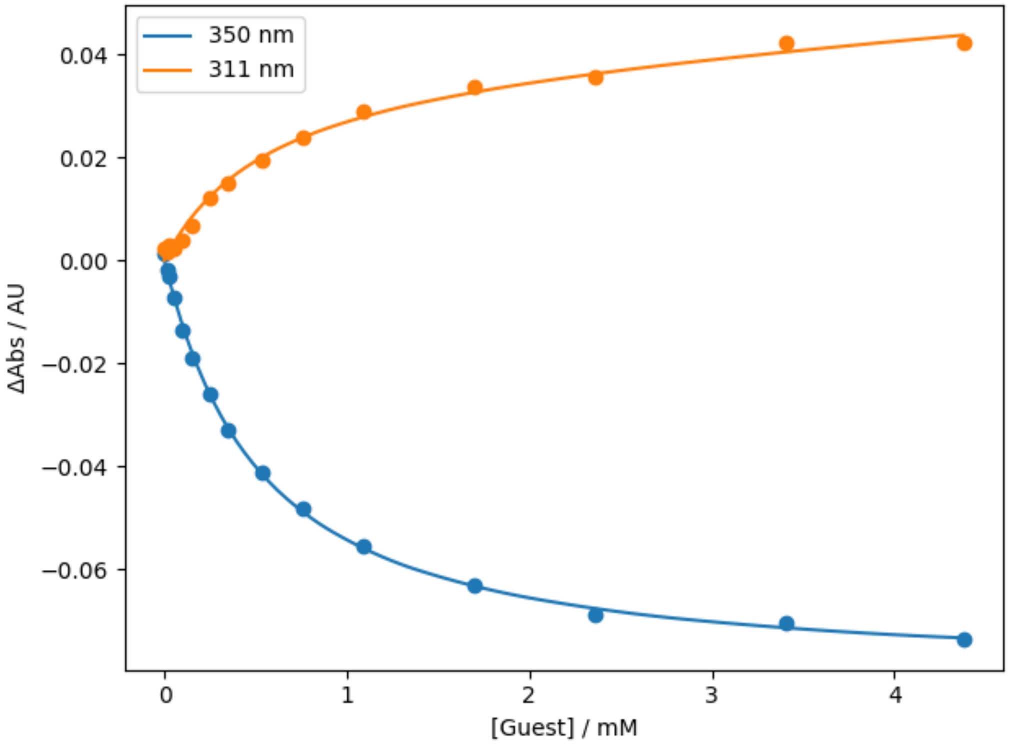

Figure S.31 -*3* Changes in UV/Vis absorbance from the titrations of **Bu_3PO** into **3** in *n*-octane (at 298 K) at selected wavelengths (points), and the fits to a 1:1 binding isotherm with guest absorption (solid lines).

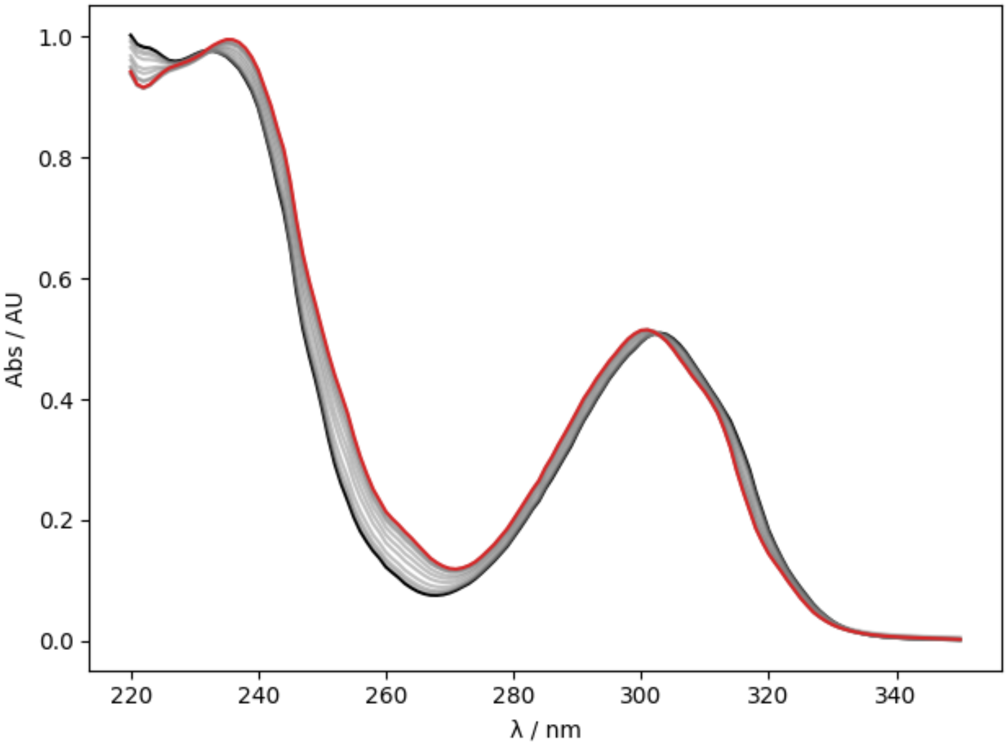

Figure S.32 - UV/Vis absorption spectra for the titration of **Bu_3PO** into **4** (in *n*-octane, at 298K). The UV/Vis spectrum of the host **4** and the final point of the titration are reported in black and in red, respectively.

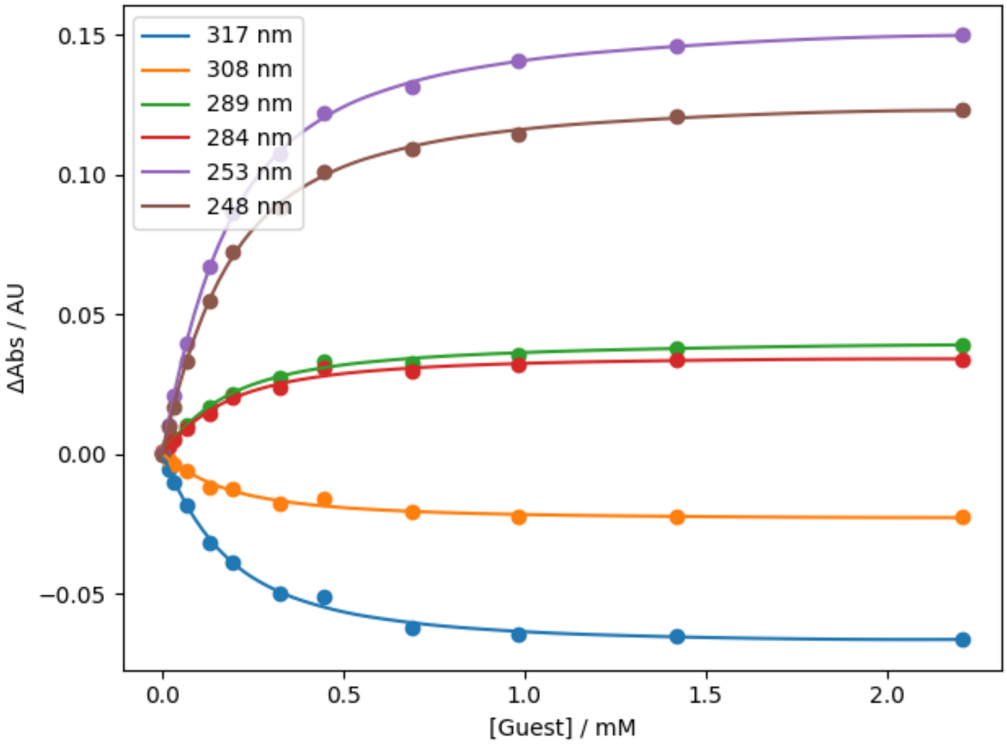

Figure S.33 -*4* Changes in UV/Vis absorbance from the titrations of **Bu_3PO** into **4** in *n*-octane (at 298 K) at selected wavelengths (points), and the fits to a 1:1 binding isotherm with guest absorption (solid lines).

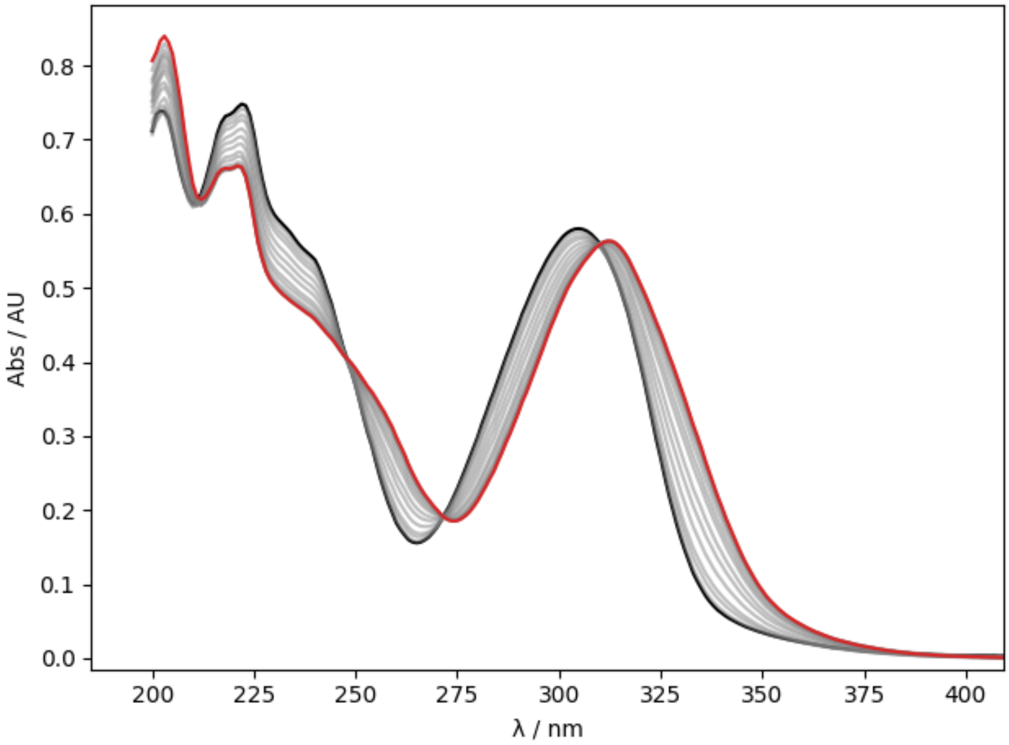

Figure S.34 - UV/Vis absorption spectra for the titration of **Bu_3PO** into **5** (in *n*-octane, at 298K). The UV/Vis spectrum of the host **5** and the final point of the titration are reported in black and in red, respectively.

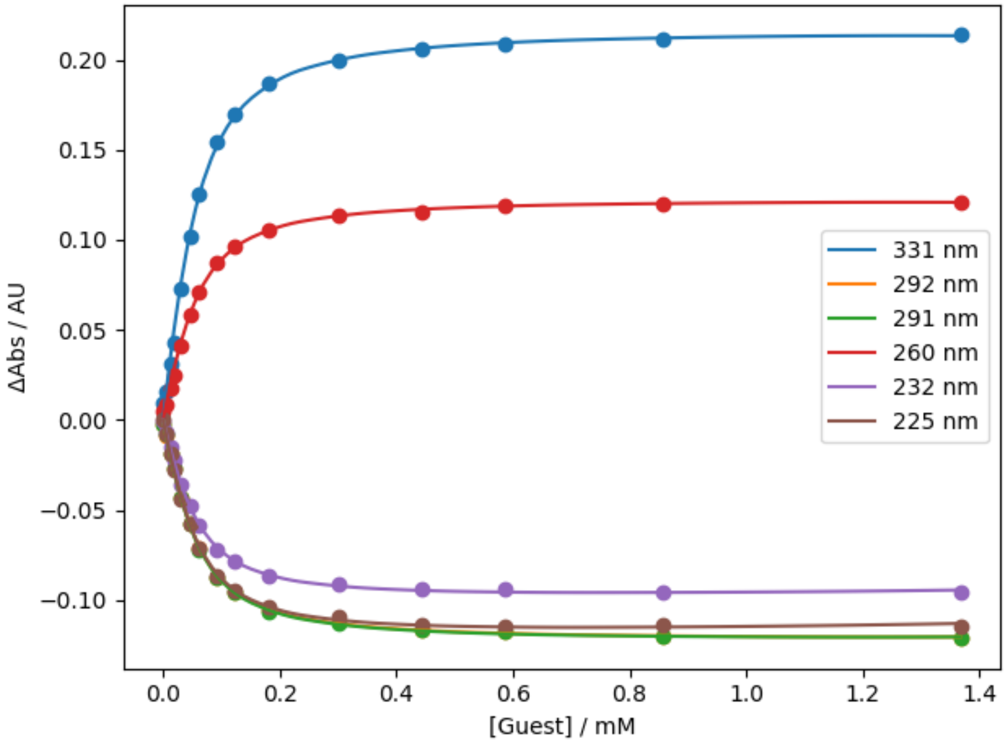

Figure S.35 -*5* Changes in UV/Vis absorbance from the titrations of **Bu_3PO** into **5** in *n*-octane (at 298 K) at selected wavelengths (points), and the fits to a 1:1 binding isotherm with guest absorption (solid lines).

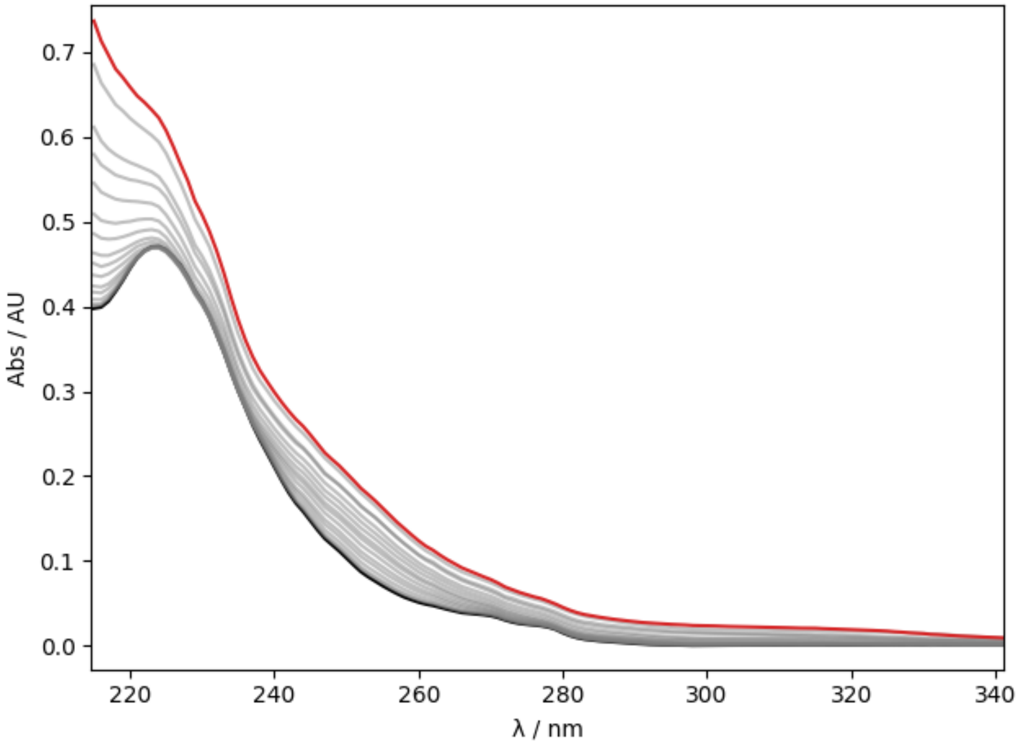

Figure S.36 - UV/Vis absorption spectra for the titration of **Bu_3PO** into **6** (in *n*-octane, at 298K). The UV/Vis spectrum of the host **6** and the final point of the titration are reported in black and in red, respectively.

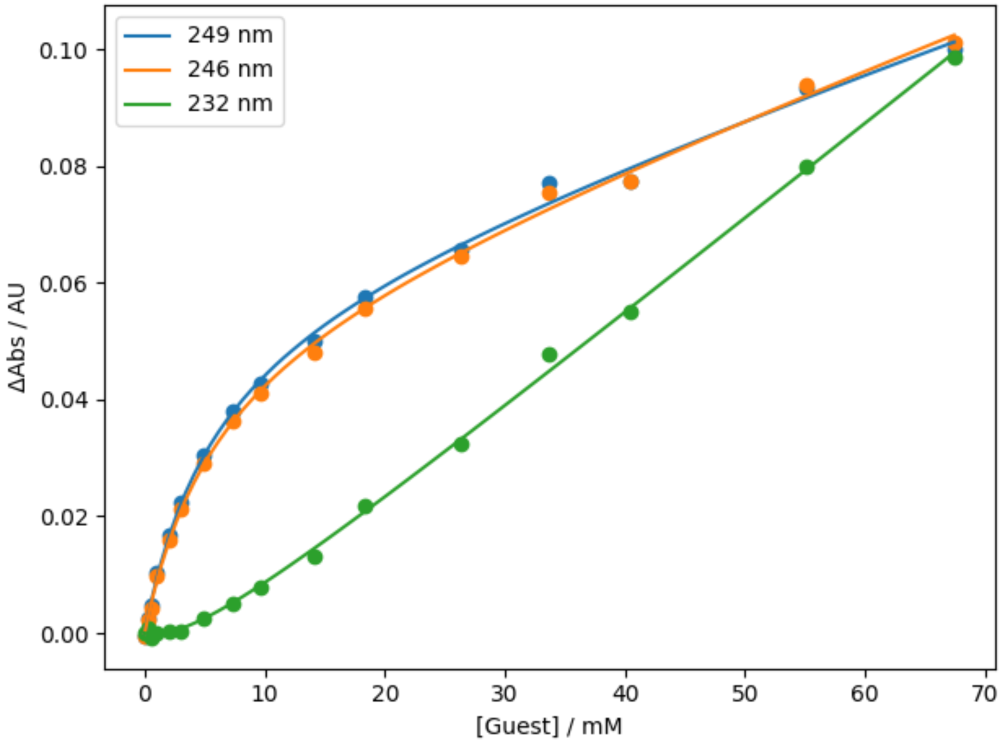

Figure S.37 -*6* Changes in UV/Vis absorbance from the titrations of **Bu_3PO** into **6** in *n*-octane (at 298 K) at selected wavelengths (points), and the fits to a 1:1 binding isotherm with guest absorption (solid lines).

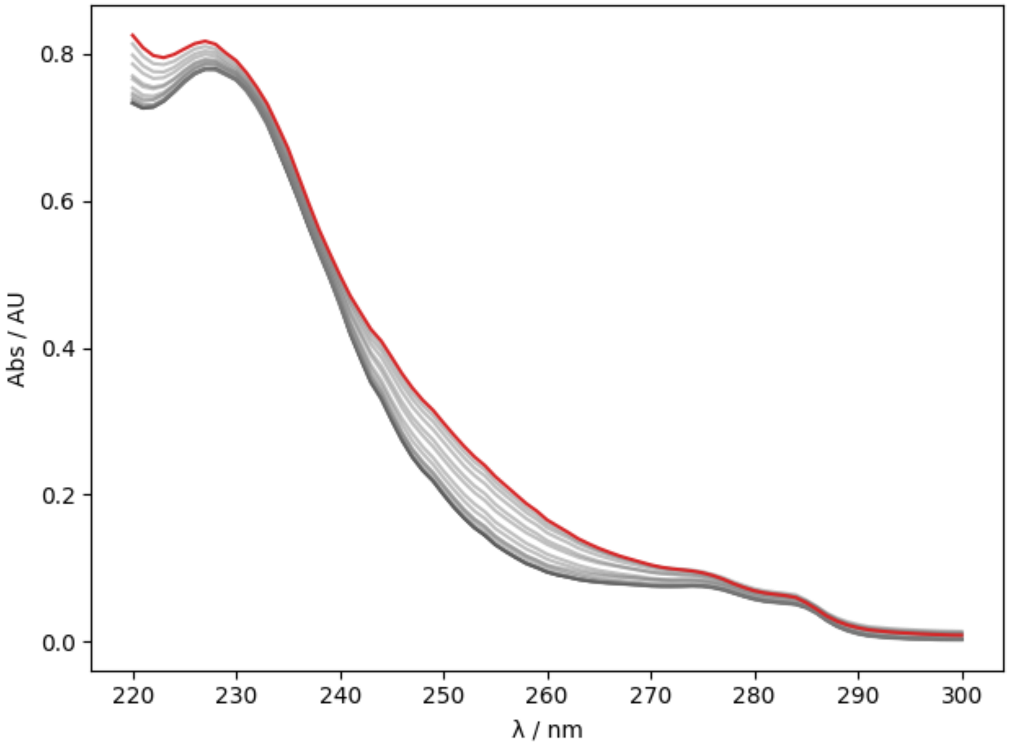

Figure S.38 - UV/Vis absorption spectra for the titration of **Bu_3PO** into **7** (in *n*-octane, at 298K). The UV/Vis spectrum of the host **7** and the final point of the titration are reported in black and in red, respectively.

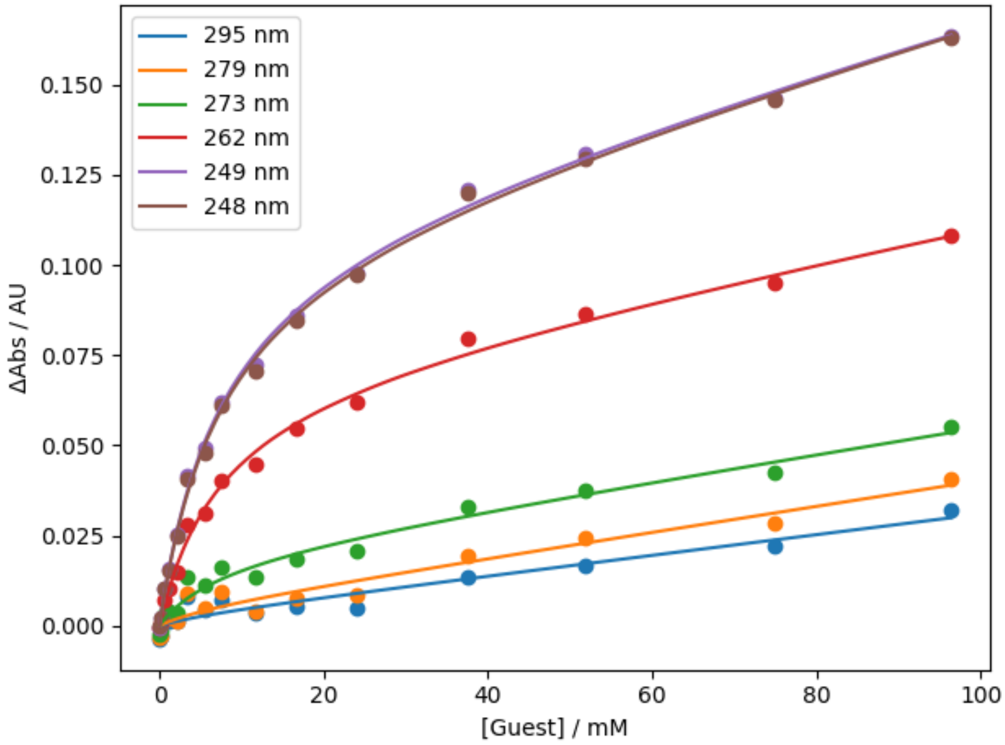

Figure S.39 -*7* Changes in UV/Vis absorbance from the titrations of **Bu_3PO** into **7** in *n*-octane (at 298 K) at selected wavelengths (points), and the fits to a 1:1 binding isotherm with guest absorption (solid lines).

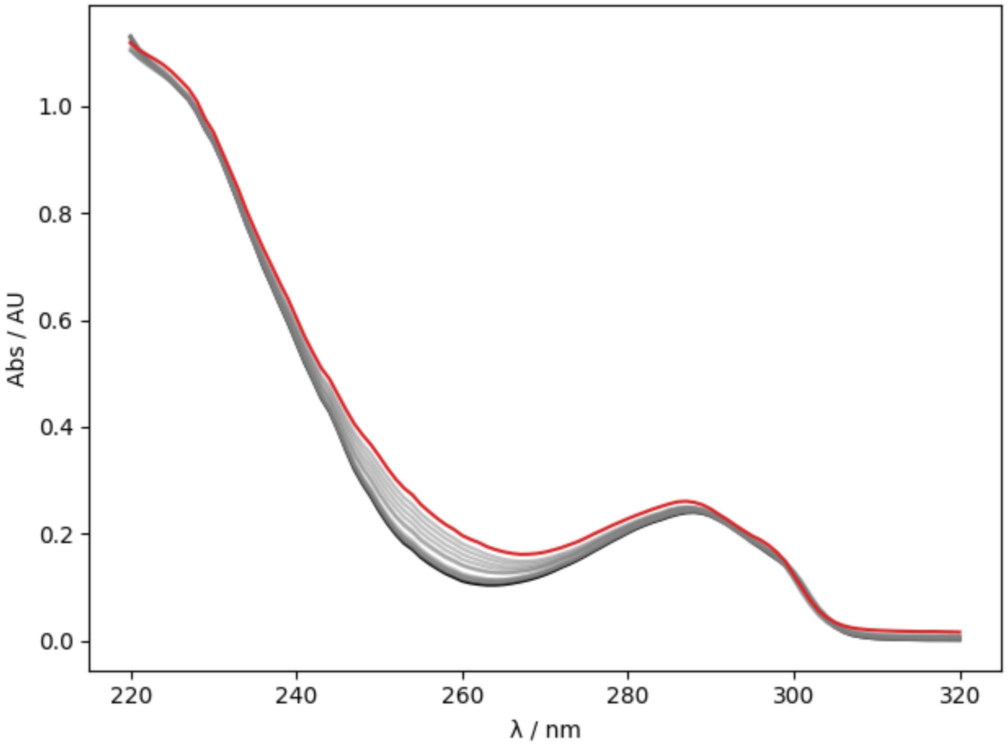

Figure S.40 - UV/Vis absorption spectra for the titration of **Bu_3PO** into **8** (in *n*-octane, at 298K). The UV/Vis spectrum of the host **8** and the final point of the titration are reported in black and in red, respectively.

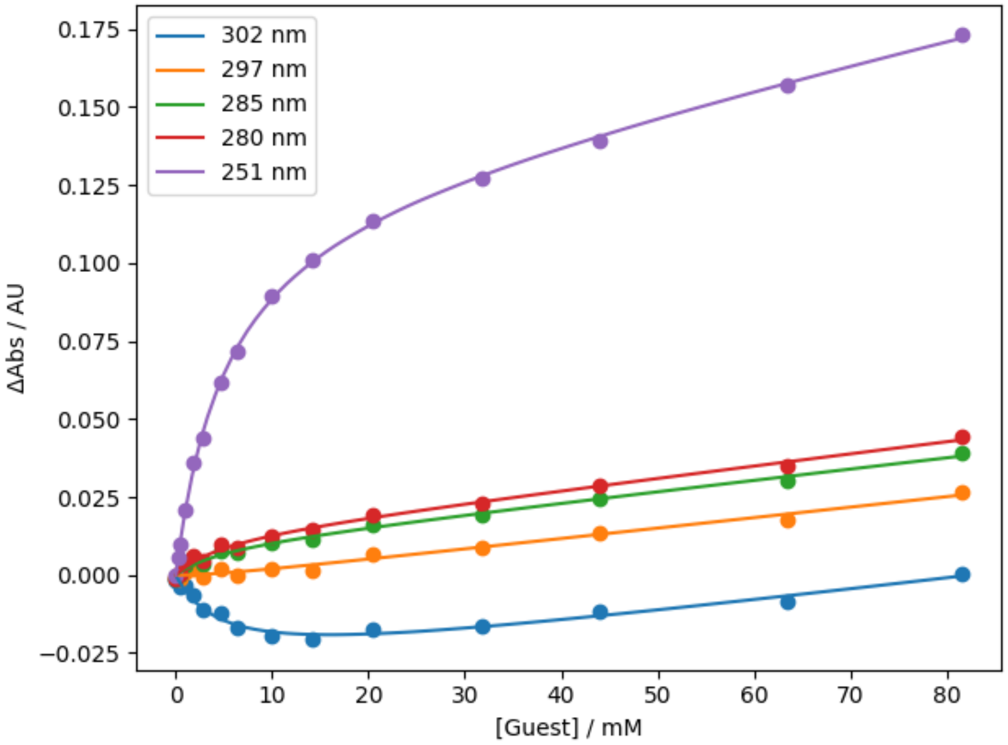

Figure S.41 -*8* Changes in UV/Vis absorbance from the titrations of **Bu_3PO** into **8** in *n*-octane (at 298 K) at selected wavelengths (points), and the fits to a 1:1 binding isotherm with guest absorption (solid lines).

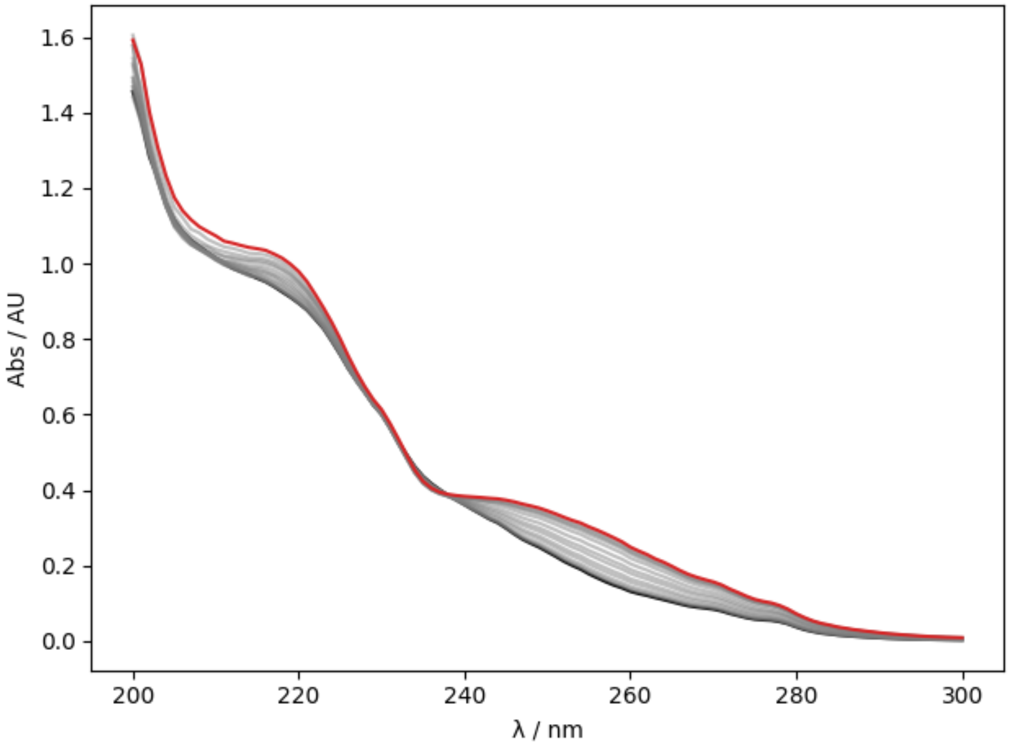

Figure S.42 - UV/Vis absorption spectra for the titration of **Bu_3PO** into **9** (in *n*-octane, at 298K). The UV/Vis spectrum of the host **9** and the final point of the titration are reported in black and in red, respectively.

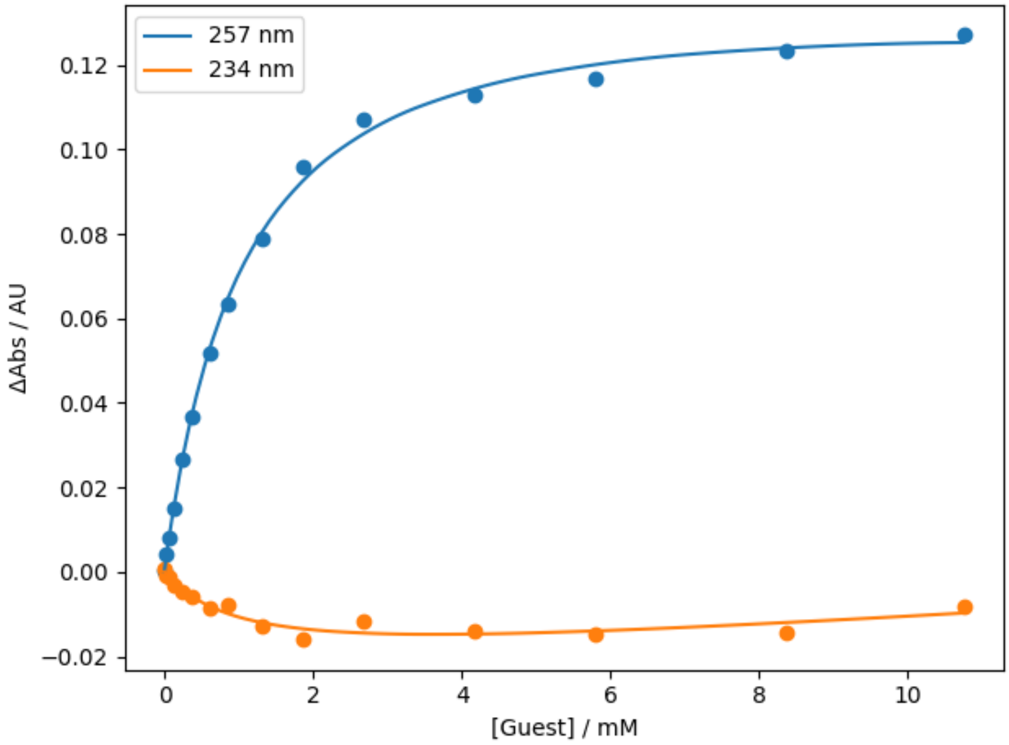

Figure S.43 -*9* Changes in UV/Vis absorbance from the titrations of **Bu_3PO** into **9** in *n*-octane (at 298 K) at selected wavelengths (points), and the fits to a 1:1 binding isotherm with guest absorption (solid lines).

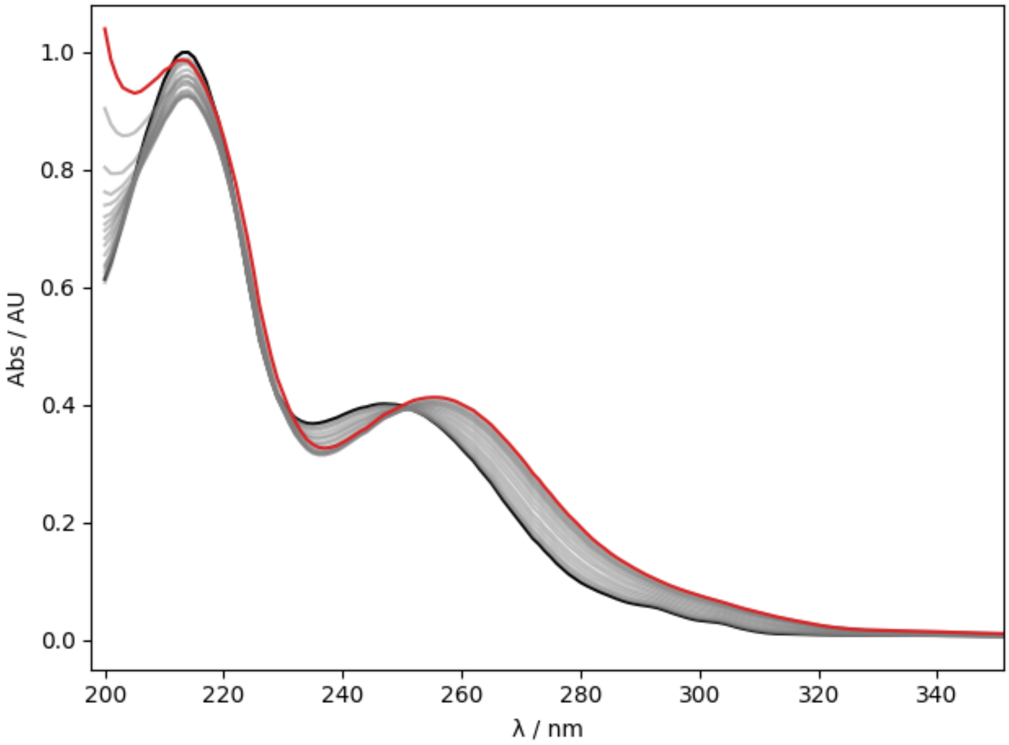

Figure S.44 - UV/Vis absorption spectra for the titration of **Bu_3PO** into **10** (in *n*-octane, at 298K). The UV/Vis spectrum of the host **10** and the final point of the titration are reported in black and in red, respectively.

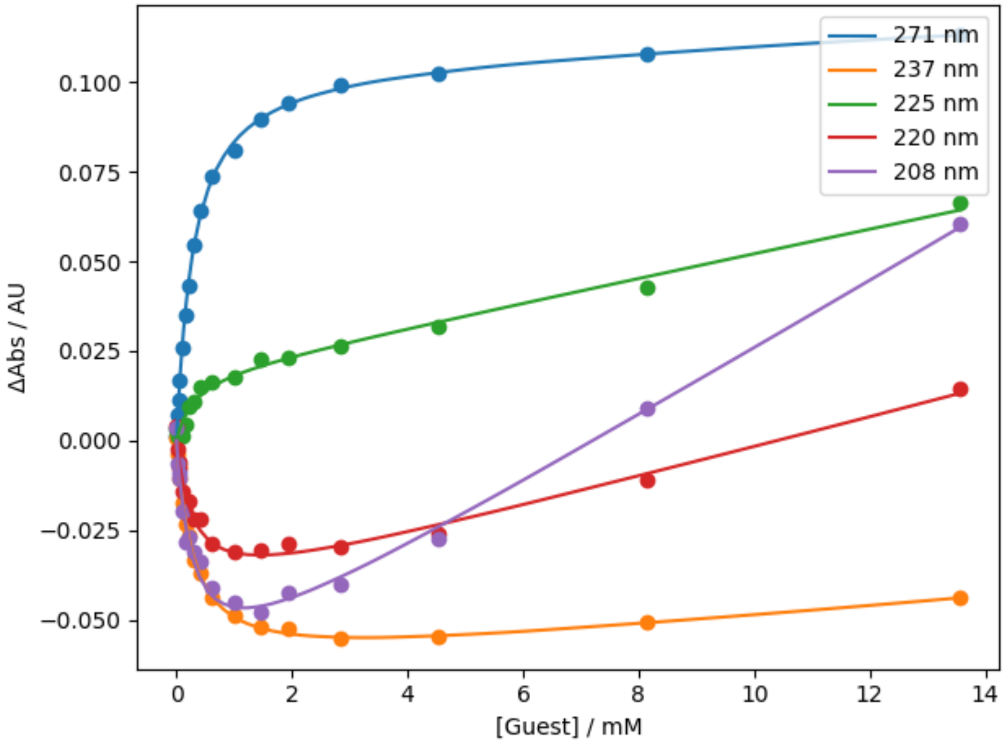

Figure S.45 -*10* Changes in UV/Vis absorbance from the titrations of **Bu_3PO** into **10** in *n*-octane (at 298 K) at selected wavelengths (points), and the fits to a 1:1 binding isotherm with guest absorption (solid lines).

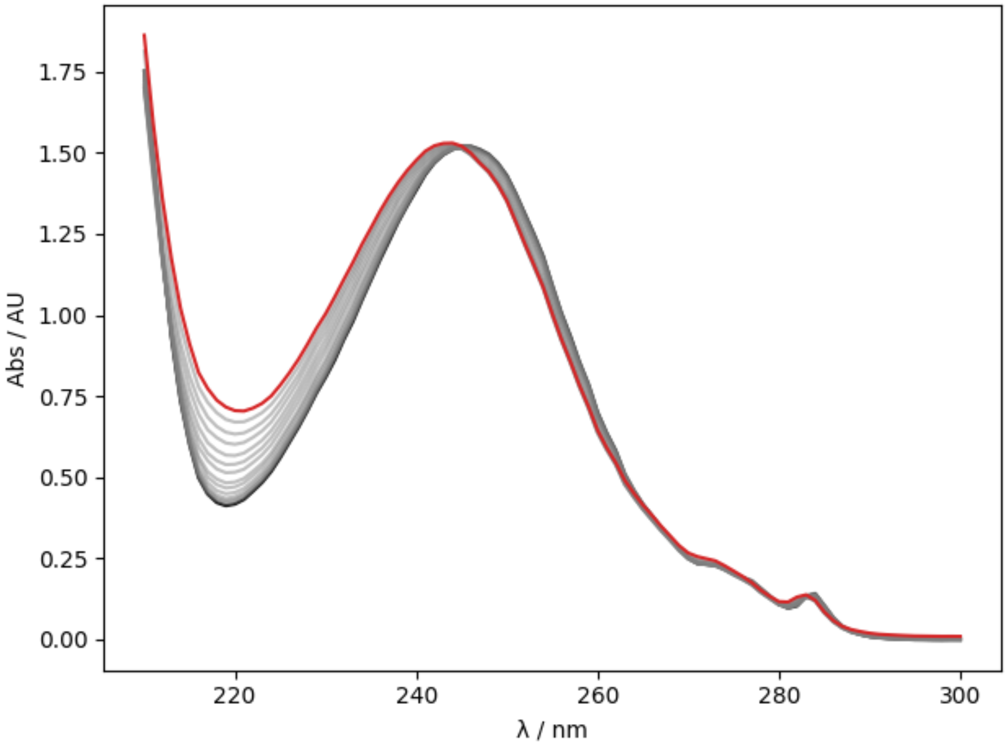

Figure S.46 - UV/Vis absorption spectra for the titration of **Bu_3PO** into **12** (in *n*-octane, at 298K). The UV/Vis spectrum of the host **12** and the final point of the titration are reported in black and in red, respectively.

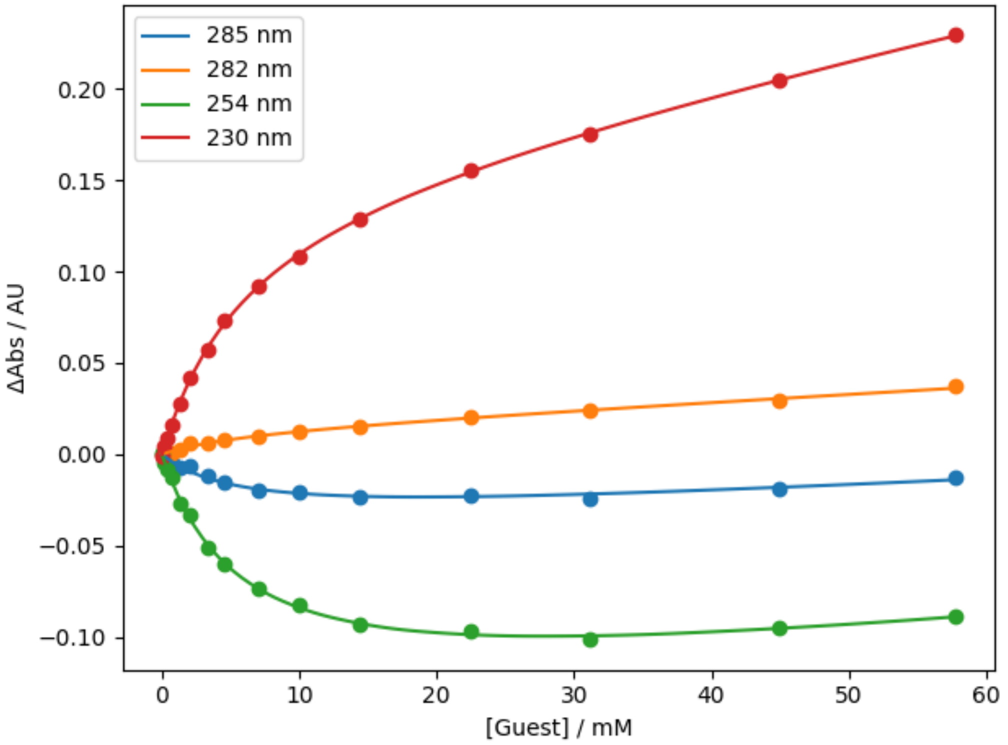

Figure S.47 *-11* Changes in UV/Vis absorbance from the titrations of **Bu_3PO** into **12** in *n*-octane (at 298 K) at selected wavelengths (points), and the fits to a 1:1 binding isotherm with guest absorption (solid lines).

1D NOE Difference Spectra

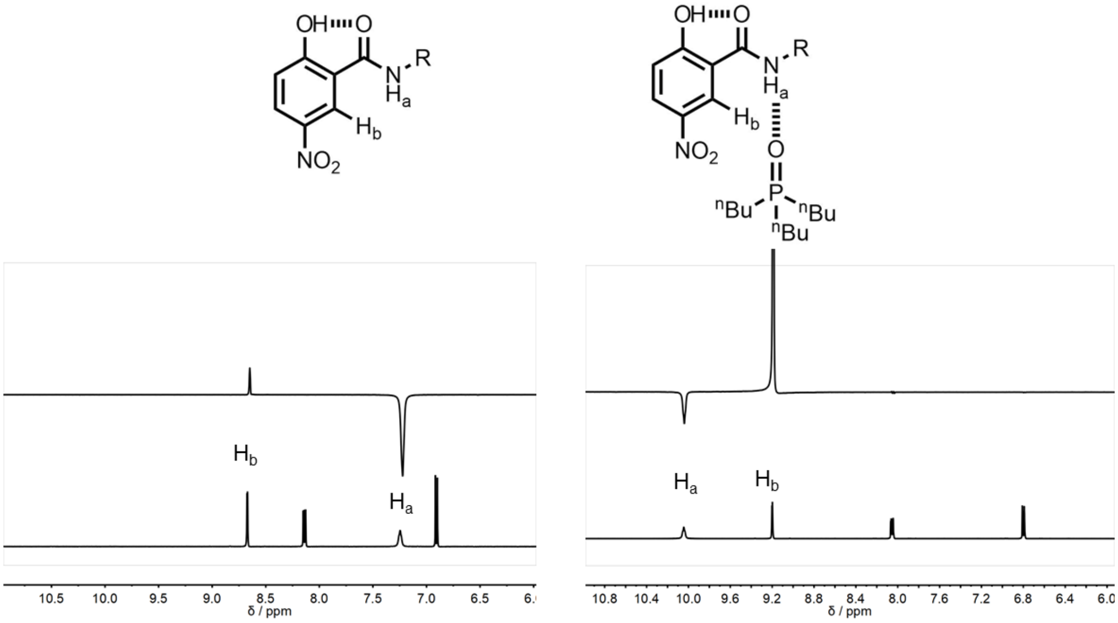

Figure S.48 – 400 MHz ^{1}H NMR NOE difference spectra for irradiation of H_a (top) and ^{1}H (bottom) spectra of free **5** (left) and the **5**•Bu_3PO (right) complex with protons H_a and H_b labelled. [**5**] = 14.0 mM in both, in the complex [Bu_3PO] = 47.5 mM , T = 298 K, in *n*-octane.

^{1}H NMR Titrations

Titrations were carried out on a Bruker 400 MHz spectrometer, using standard titration protocols. A 2 mL sample of the host was prepared at a concentration of 0.18-0.22 mM in spectroscopic grade *n*-octane. The NMR spectrum of the host solution (0.6 mL) was recorded. The guest solution was prepared at a concentration of using the host solution as solvent. Aliquots of the guest solution were successively added to the NMR sample tube containing the host solution, and the ^{1}H NMR spectrum was recorded after each addition. The NMR spectra were analysed using a purpose-built Python script to fit the changes in the chemical shifts for different protons to a 1:1 binding isotherm, by optimizing the association constant and absorption of the free and bound host using a purpose-written Python script.

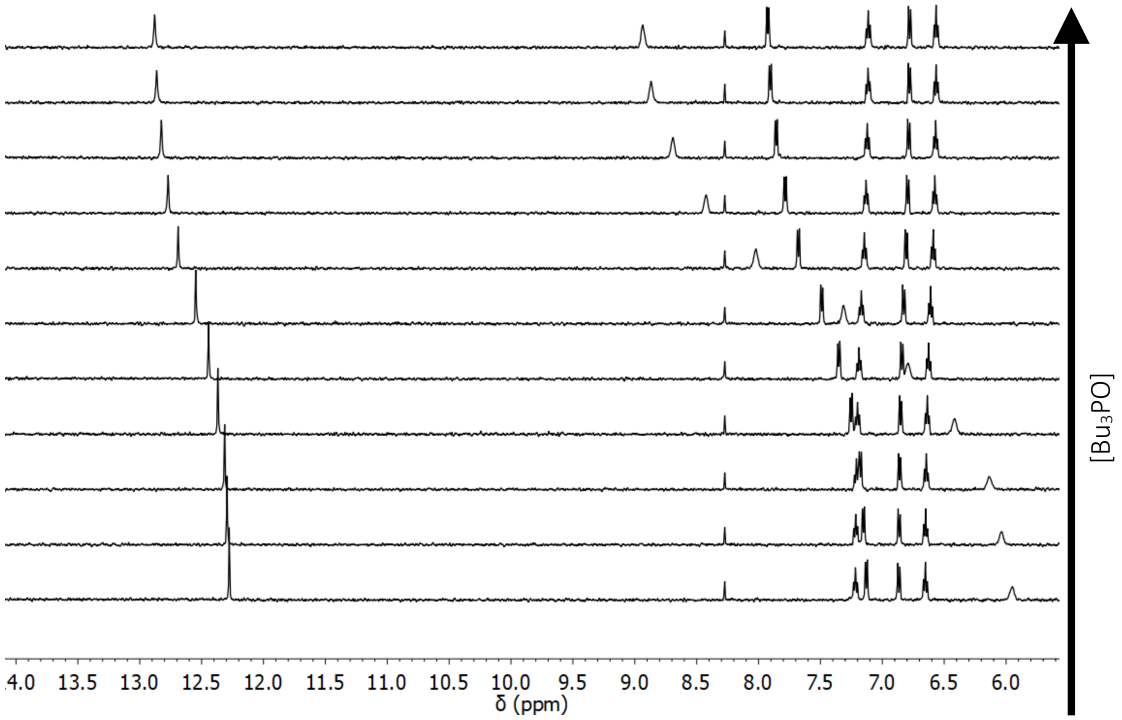

Figure S.49 - Spectra from the 400 MHz ^{1}H NMR titration of **Bu_3PO** (0–14 mM) into **1** (2 mM) in *n*-octane at 298 K.

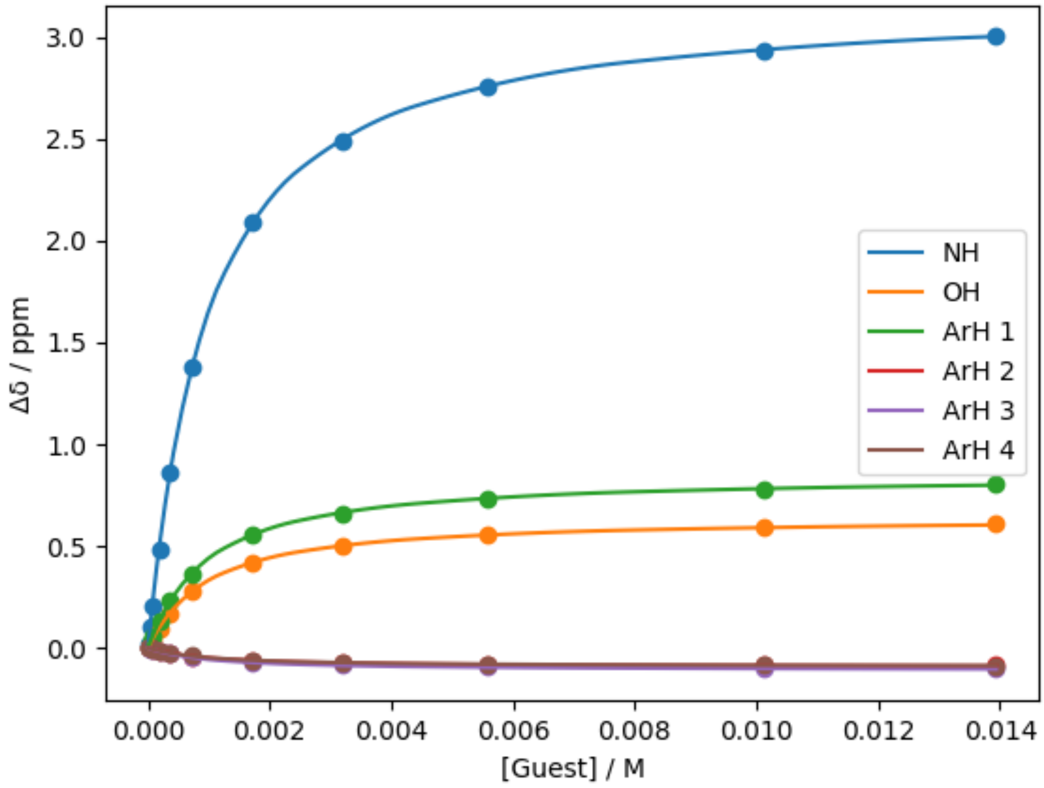

Figure S.50 - Change in chemical shift during the 400 MHz ^{1}H NMR titration of **Bu_3PO** into **1** in *n*-octane at 298 K (points), and the fits to a 1:1 binding isotherm (solid lines).

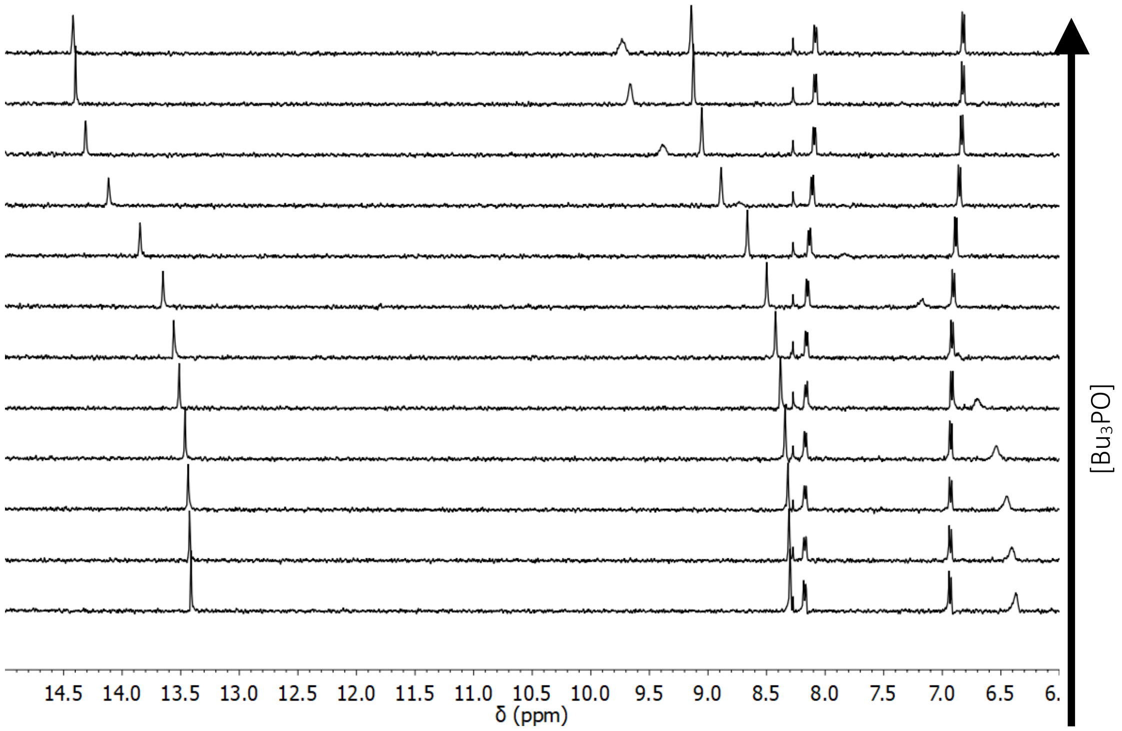

Figure S.51 - Spectra from the 400 MHz ^{1}H NMR titration of **Bu₃PO** (0–0.44 mM) into **5** (2 mM) in *n*-octane at 298 K.

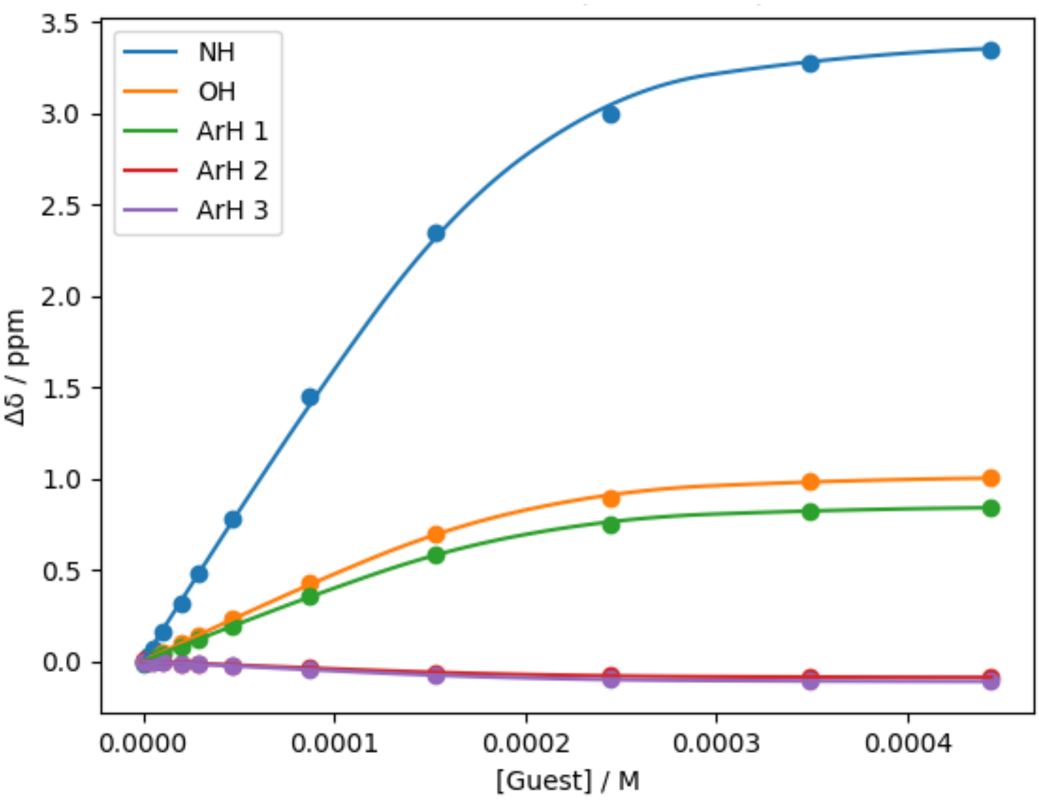

Figure S.52 - Change in chemical shift during the 400 MHz ^{1}H NMR titration of **Bu₃PO** into **5** in *n*-octane at 298 K (points), and the fits to a 1:1 binding isotherm (solid lines).

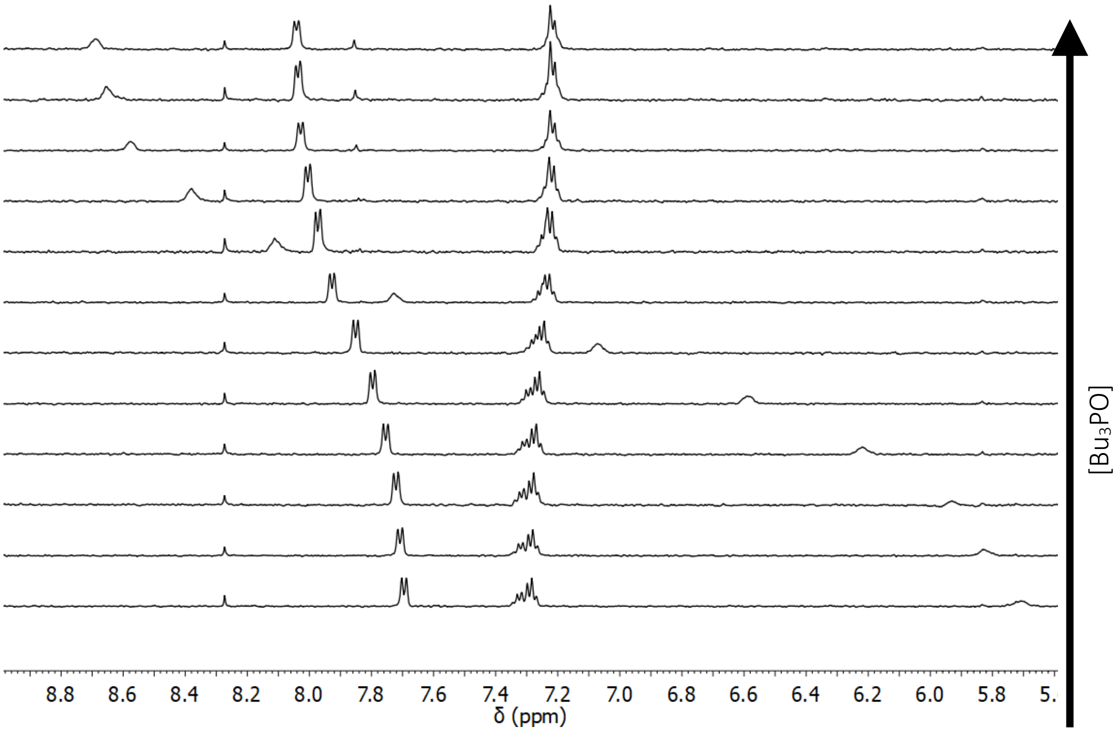

Figure S.53 - Spectra from the 400 MHz ^{1}H NMR titration of **Bu$_3$PO** (0–82 mM) into **6** (0.2 mM) in *n*-octane at 298 K.

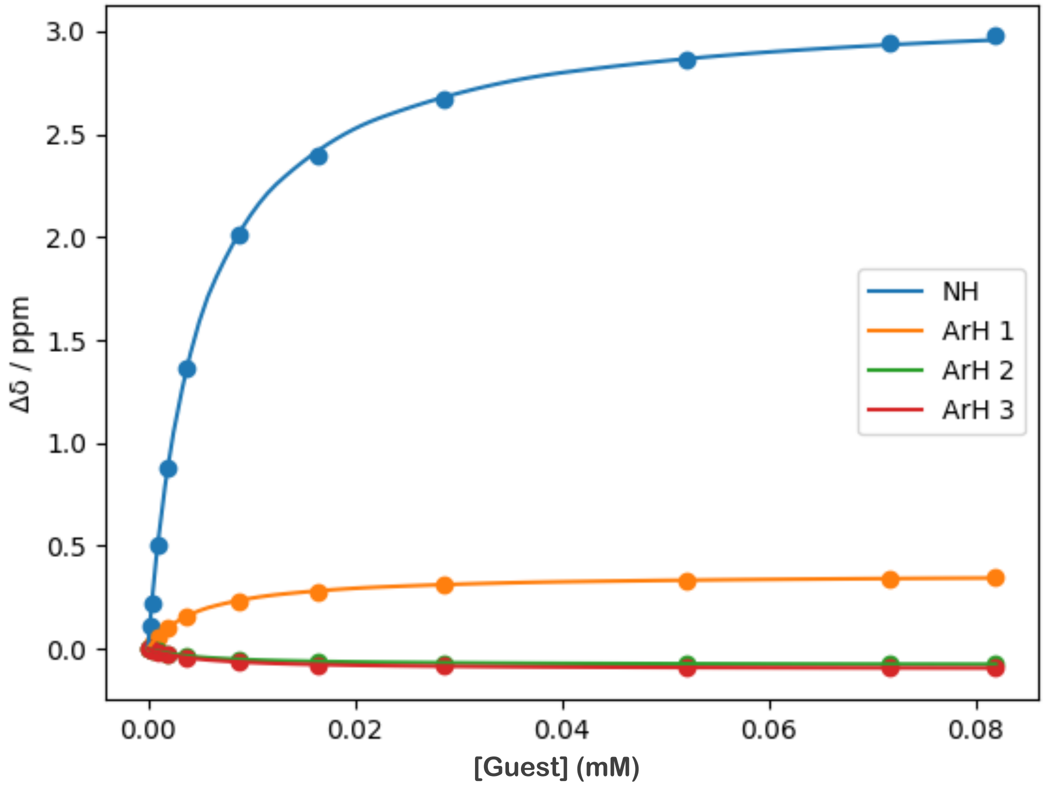

Figure S.54 - Change in chemical shift during the 400 MHz ^{1}H NMR titration of **Bu$_3$PO** into **6** in *n*-octane at 298 K (points), and the fits to a 1:1 binding isotherm (solid lines).

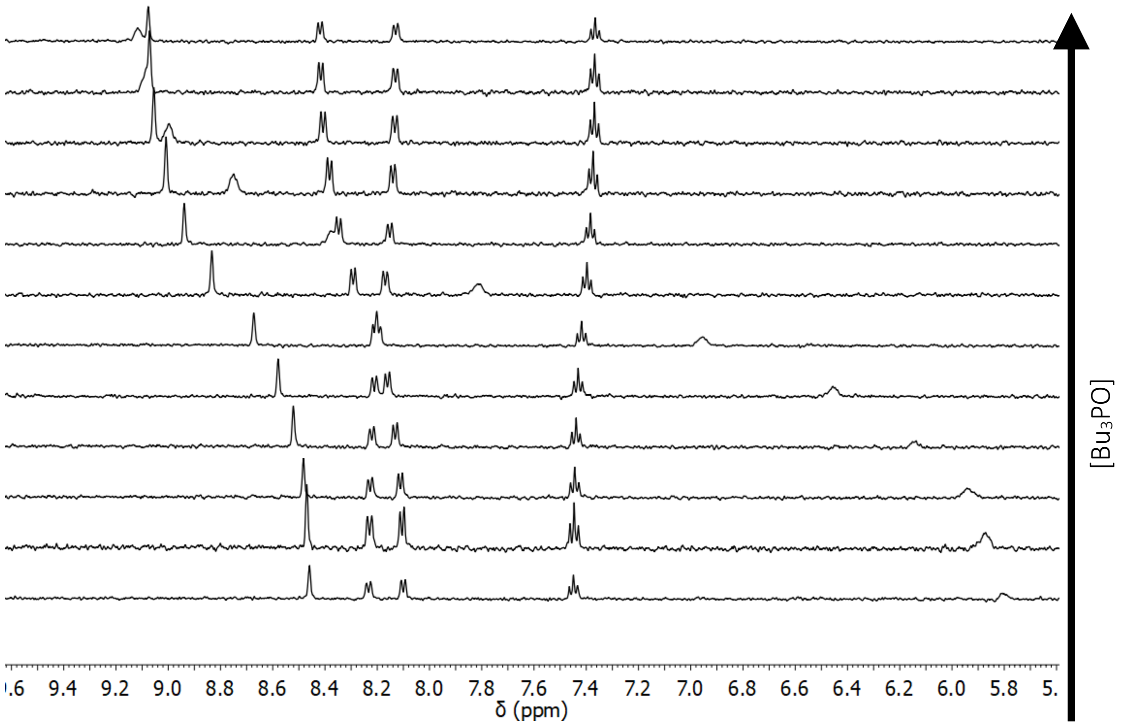

Figure S.55 - Spectra from the 400 MHz ^{1}H NMR titration of **Bu_3PO** (0–3.7 mM) into **10** (0.2 mM) in *n*-octane at 298 K.

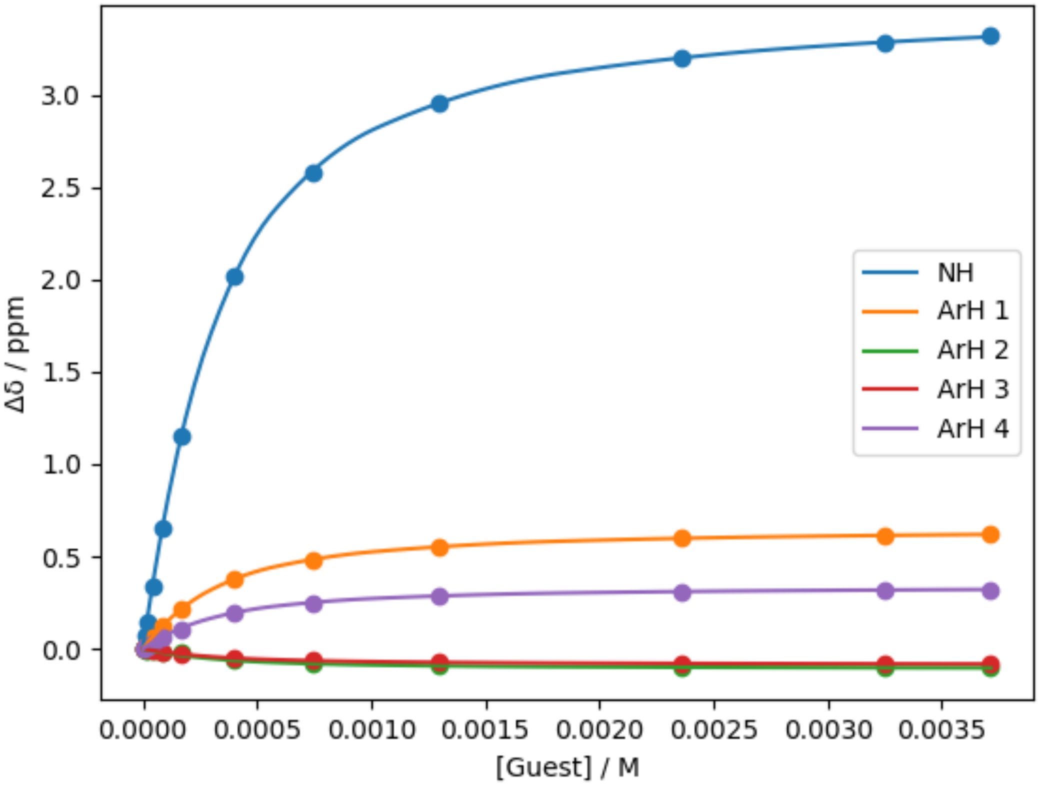

Figure S.56 - Change in chemical shift during the 400 MHz ^{1}H NMR titration of **Bu_3PO** into **10** in *n*-octane at 298 K (points), and the fits to a 1:1 binding isotherm (solid lines).

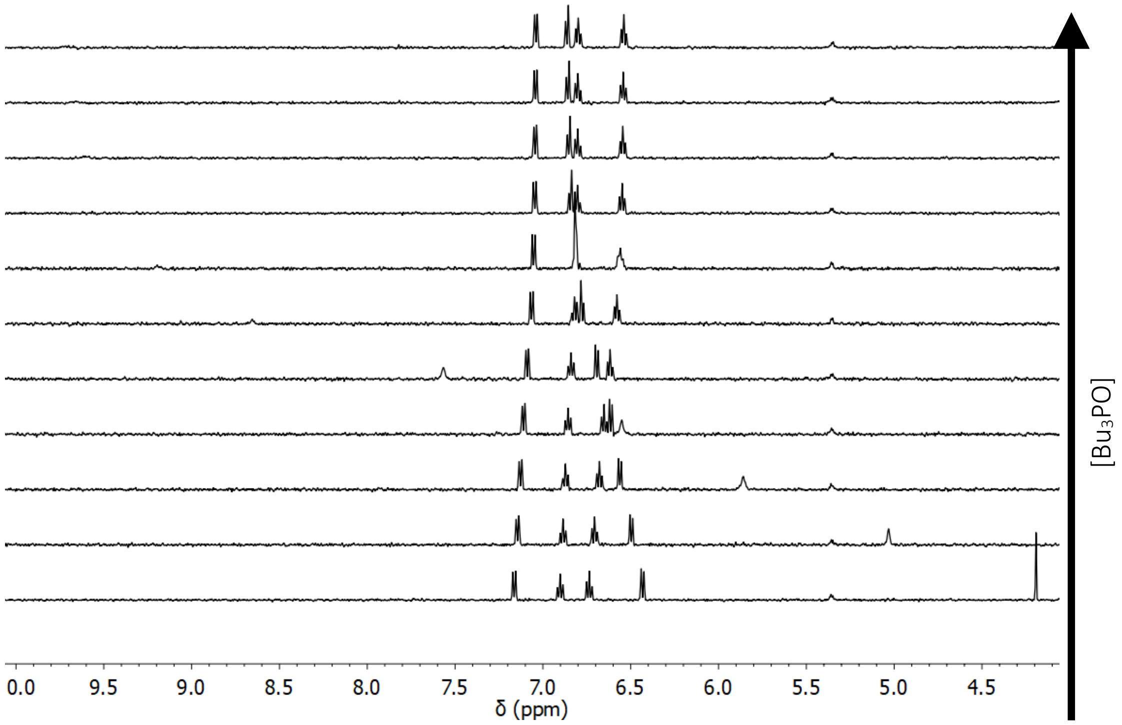

Figure S.57 - Spectra from the 400 MHz 1H NMR titration of **Bu_3PO** (0–16 mM) into **2-^{t}Bu-phenol** (0.2 mM) in *n*-octane at 298 K.

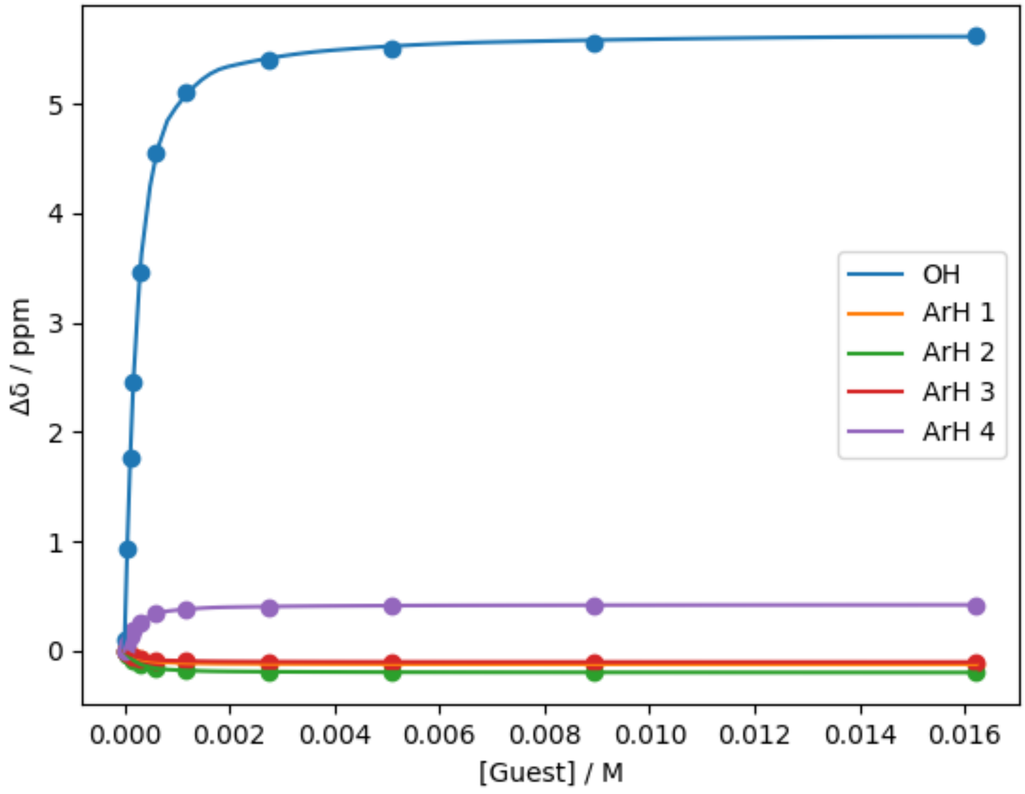

Figure S.58 - Change in chemical shift during the 400 MHz ^{1}H NMR titration of **Bu_3PO** into **2-^{t}Bu-phenol** in *n*-octane at 298 K (points), and the fits to a 1:1 binding isotherm (solid lines).

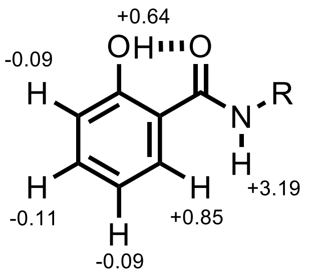

1

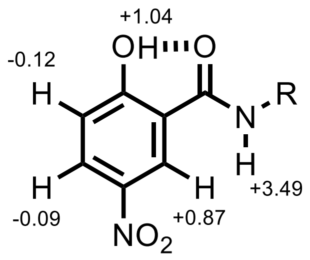

5

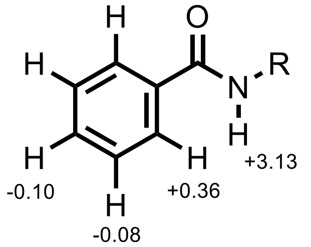

6

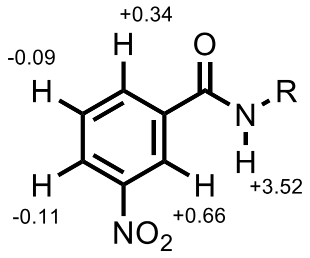

10

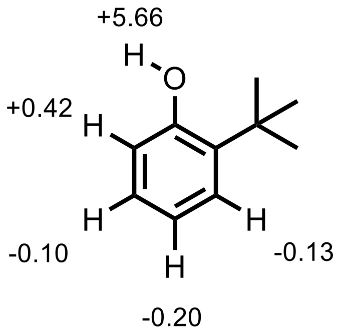

13

Figure S.59 - Limiting complexation-induced changes in ^{1}H NMR chemical shift (ppm) for formation of 1:1 complexes with tri-*n*-butyl phosphine oxide in *n*-octane at 298 K.

Computational Analysis

All molecular mechanics calculations used Schrödinger's Maestro software (2016 Edition) with $CHCl_3$ as implicit solvent and the OPLS3 forcefield.[2] First an energy minimisation was carried out (10000 iterations, convergence on gradient of 0.01). The minimisation result was then used as the basis for a conformational search (mixed torsional/low mode sampling, 10000 steps). For the secondary amides a torsion constraint to force the amide trans was used, which was defined as the H-N-C=O dihedral angle equal to 180±30 °. The lowest energy conformation was then used as a basis for a Jaguar DFT optimisation using a 6-31G** basis set[3], a hybrid B3LYP functional[4-7] and a nonrelativistic Hamiltonian. The solvation model used was a Poisson-Boltzmann Finite element model.[8] The resulting structures were subject to a purpose-built JavaScript calculation using NWChem [9] to calculate α parameters for the NH group.

Compound number	calculated α
**1**	3.35
**2**	3.15
**3**	3.32
**4**	3.93
**5**	4.23
**6**	2.94
**7**	2.64
**8**	2.78
**9**	3.30
**10**	3.69

Table 1 –Calculated α values for compounds **1-10**.

References

1. G.R. Fulmer, A.J.M. Miller, N.H. Sherden, H.E. Gottlieb, A. Nudelman, B.M. Stoltz, Bercaw, and K.I. Goldberg, *Organometallics,* **2010**, 29, 2176-2179
2. T.A. Halgren, *J. Comput. Chem.*, **1999**, 20 (7), 720-729
3. P.C. Hariharan and J.A. Pople, *Theor. Chim. Acta.*, **1973**, 28, 213-222
4. A.D. Becke*, J. Chem. Phys*., **1993**, 98, 5648-5652
5. C. Lee, W. Yang and R.G. Parr, *Phys. Rev. B*, **1988**, 37, 785-789
6. S.H. Vosko, L. Wilk and M. Nusair, *Can. J. Phys*., **1980**, 58, 1200-1211
7. P.J. Stephens, F.J. Devlin, C.F. Chabalowski and M.J. Frisch, *J. Phys. Chem*., **1994,** 98, 11623-1162
8. D.J. Tannor, B. Marten, R. Murphy, R.A. Friesner, D. Stikoff, A. Nicholls, M. Ringnalda, W.A. Goddard III and B. Honig, *J. Am. Chem. Soc.*, **1994**, 116, 11875-11882
9. M.D. Driver, M.J. Williamson, N. DeMitri, T. Nikolov and C.A. Hunter, *J. Chem. Inf. Model.* **2021**, 61, 11, 5331–5335
